# LRIT1 Modulates Adaptive Changes in Synaptic Communication of Cone Photoreceptors

**DOI:** 10.1016/j.celrep.2018.03.008

**Published:** 2018-03-27

**Authors:** Ignacio Sarria, Yan Cao, Yuchen Wang, Norianne T. Ingram, Cesare Orlandi, Naomi Kamasawa, Alexander V. Kolesnikov, Johan Pahlberg, Vladimir J. Kefalov, Alapakkam P. Sampath, Kirill A. Martemyanov

**Affiliations:** 1 Department of Neuroscience, The Scripps Research Institute, Jupiter, FL 33458, USA; 2 Department of Ophthalmology, Stein Eye Institute, UCLA School of Medicine, Los Angeles, CA 90095, USA; 3 Electron Microscopy Core Facility, Max Planck Florida Institute, Jupiter, FL 33458, USA; 4 Department of Ophthalmology and Visual Sciences, Washington University School of Medicine, St. Louis, MO 63110, USA

## Abstract

Cone photoreceptors scale dynamically the sensitivity of responses to maintain responsiveness across wide range of changes in luminance. Synaptic changes contribute to this adaptation, but how this process is coordinated at the molecular level is poorly understood. Here, we report that a cell adhesion-like molecule, LRIT1, is enriched selectively at cone photoreceptor synapses where it engages in a *trans*-synaptic interaction with mGluR6, the principal receptor in postsynaptic ON-bipolar cells. The levels of LRIT1 are regulated by the neurotransmitter release apparatus that controls photoreceptor output. Knockout of LRIT1 in mice increases the sensitivity of cone synaptic signaling while impairing its ability to adapt to background light without overtly influencing the morphology or molecular composition of photoreceptor synapses. Accordingly, mice lacking LRIT1 show visual deficits under conditions requiring temporally challenging discrimination of visual signals in steady background light. These observations reveal molecular mechanisms involved in scaling synaptic communication in the retina.

## In Brief

Sarria et al. show LRIT1 accumulates at photoreceptors synapses. It forms a complex with a key receptor involved in processing neurotransmitter output of photoreceptors. They show that this protein plays an essential role in synaptic communication of cone photoreceptors and impacts daylight vision.

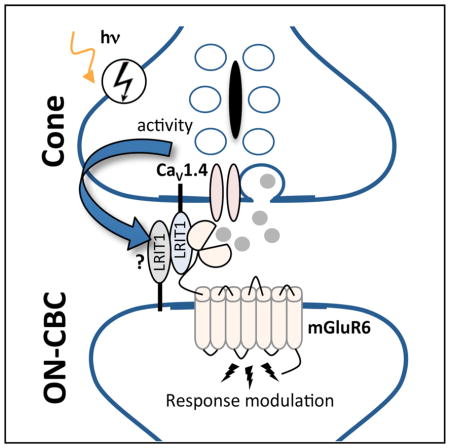

## INTRODUCTION

In the vertebrate retina, the rod and cone photoreceptors respond to incident light by modulating their membrane potential. This signal is transmitted to their bipolar cells and eventually to higher visual centers that enable our complex visual experience. Rods and cones subdivide the range of the visual system by mediating light reception in different regimes of intensity (Ingram et al., 2016; Pugh and Lamb, 2000; Yau and Hardie, 2009). Rods are exquisitely sensitive and are capable of detecting single photon absorptions, yet their responses are relatively slow and their dynamic range is limited. In contrast, cones are less sensitive, but faster, cover a wider range of light intensities, and are more resistant to saturation than rods (Burkhardt, 1994; Matthews et al., 1990; Nikonov et al., 2006). Furthermore, cones normally operate under daylight conditions where light intensities vary over a wide range, requiring them to adjust dynamically the sensitivity of their responses (Korenbrot, 2012; Soo et al., 2008; Stockman et al., 2006).

Photoreceptors use a variety of mechanisms to modulate their gain to maintain responsiveness as light intensities vary (Govardovskii et al., 2000; Morshedian and Fain, 2017; Pugh et al., 1999); one critical control point is at their synapse with bipolar cells (Wu, 1994). Light hyperpolarizes the photoreceptor membrane potential, which biases voltage-gated Ca_V_1.4 Ca^2+^ channels in their axonal terminals toward the closed state, thereby reducing Ca^2+^ influx and glutamate release (Heidelberger et al., 2005; Joiner and Lee, 2015). Modulating neurotransmitter release through a combination of intrinsic mechanisms that control the release machinery (Thoreson et al., 2004; Yang and Wu, 1997) as well as through the negative feedback from the downstream neurons (Kramer and Davenport, 2015; Vroman et al., 2013) has been established as a powerful means for adjusting the gain of the photoreceptor synaptic output. However, the molecular mechanisms underlying this process, particularly as it varies between rods and cones, remain controversial and poorly understood. Even less clear is how photoreceptors coordinate the gain of the synaptic transmission with their dedicated postsynaptic bipolar cells, which are responsible for decoding photoreceptor signals.

In postsynaptic bipolar cell dendrites, the reduction in glutamate release is detected by two classes of bipolar cells; the OFF type neurons (OFF-BCs) that predominantly contact cones in the mammalian retina and utilize ionotropic glutamate receptors to preserve the hyperpolarizing photoreceptor light response, and the ON type (ON-BCs) that use metabotropic mGluR6 receptors to generate a depolarizing response that inverts the sign of the photoreceptor response. We now appreciate that there are at least 8 subtypes of ON-BCs showing functional specialization and selectivity in establishing contacts with either rods or cones (Euler et al., 2014; Hoon et al., 2014).

The core of the postsynaptic mGluR6 pathway that activates ON-BCs includes the heterotrimeric G protein Gα_o_β3γ13, which in turn gates the effector ion channel TRPM1 (Martemyanov and Sampath, 2017; Morgans et al., 2010; Vardi et al., 2002). This signaling cascade is coordinated additionally by a host of proteins with critical roles in enabling synaptic transmission including regulator of G protein signaling (RGS) proteins, the orphan receptor GPR179, and leucine-rich repeat (LRR) proteins NYX and LRIT3, scaffolded together in a macromolecular complex (Gregg et al., 2014; Martemyanov and Sampath, 2017; Zeitz et al., 2015).

Recent findings suggest that the postsynaptic cascade of ON-BCs is further engaged in contacts with the photoreceptor presynaptic release apparatus (Cao et al., 2015; Tummala et al., 2016; Wang et al., 2017). For example, the mGluR6 is directly recruited by the rod-specific molecule ELFN1 to the Ca_V_1.4 channel complex, an interaction that is crucial for the physical assembly of rod synapses and the transmission of rod signals to rod ON-BCs (Cao et al., 2015; Wang et al., 2017). No analogous interactions have yet been reported for the cone synapses. In addition, how these interactions influence the functional properties of synapses is not understood at any metabotropic synapse. Here, we report the identification of a leucine-rich repeat (LRR) protein, LRIT1, at photoreceptor synapses that binds *trans*-synaptically to mGluR6 and facilitates synaptic adaptations of cone photoreceptors upon changes in luminance.

## RESULTS

### Identification of LRIT1 As a Component of mGluR6 Complex

**Figure 1:**
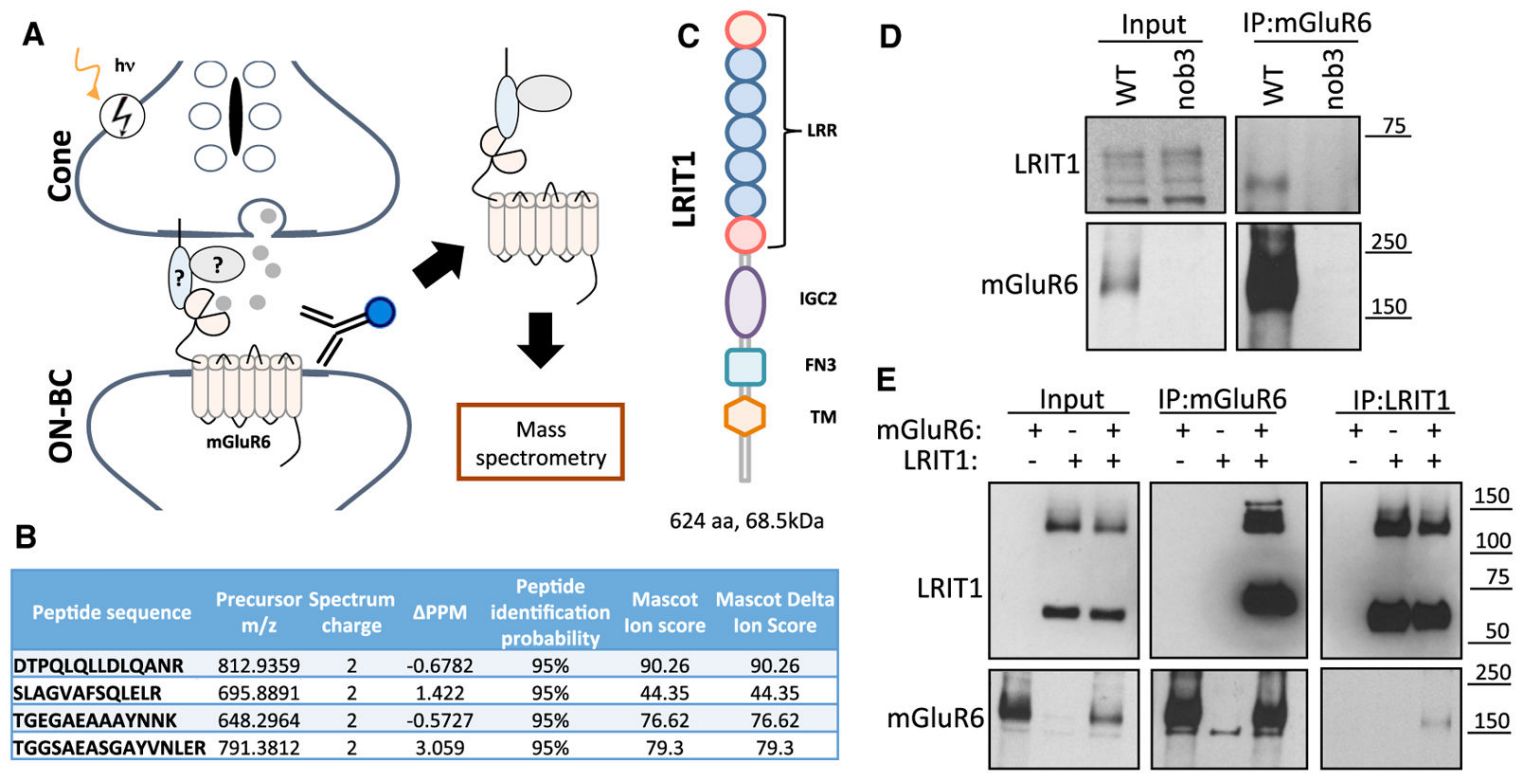
Identification of LRIT1 as mGluR6 Binding Partner (A) Schematic of the affinity purification strategy for the identification of the mGluR6 binding partners of mGluR6 at photoreceptor synapses. Specific anti-mGluR6 antibodies were used for the immunoprecipitation from membrane fractions of the retina and the eluates were subjected to mass-spectrometry. (B) Peptides matching to LRIT1 sequences identified in the mass-spectrometric experiments. Characteristics and parameters used for defining the sequences are shown. (C) Domain organization of LRIT1. LRR, leucine reach repeat; IGC2, type 2 IgG-like domain; FN3, fibronectin type 3 domain; TM, transmembrane segment. (D) Verification of mGluR6 interaction with LRIT1 by co-immunoprecipitation from retina lysates. Anti-mGluR6 antibodies were used for the immunoprecipitation (IP) and the presence of LRTI1 and mGluR6 in the IP eluates was detected by western blotting. Retinas lacking mGluR6 (nob3) were used as a specificity control. (E) Characterization of mGluR6-LRIT1 interaction in transfected HEK293T cells. Both forward and reverse immunoprecipitation experiments using anti-LRIT1 and mGluR6 antibodies were conducted following expression of the indicated constructs and the proteins were detected by western blotting.

We have previously reported a screen for the mGluR6 binding partners by immunoprecipitation with specific anti-mGluR6 antibodies followed by mass-spectrometric identification of co-purified proteins present in the eluates. In this study, we focused on candidate cell-surface molecules with potential roles in cone synaptic function (Figure 1A). In this screen, we found 4 peptides with high identification confidence that map to the sequence of the transmembrane protein, LRIT1 (Figure 1B). This protein features multiple extracellular modules including leucine-rich repeats (LRR) and IgG-like and fibronectin type III domains (Figure 1C) and belongs to the extended family of cell-adhesion like proteins (de Wit and Ghosh, 2016). To validate the specificity of the interaction, we conducted mGluR6 immunoprecipitation from wild-type mouse retinas while in parallel using retinas from nob3 mice, lacking mGluR6. When probing blots with our anti-LRIT1 antibodies, we found a single band corresponding to the predicted size of LRIT1 protein in the eluates of wild-type but not *nob3* retinas, confirming the specificity of LRIT1-mGluR6 interaction in native retinas (Figure 1D). We then probed the binding in the reconstituted system. For this, HEK293 cells were co-transfected with various combinations of mGluR6 and LRIT1 followed by reciprocal immunoprecipitation experiments. Again, we detected the robust and specific pull down of LRIT1 when mGluR6 was immunoprecipitated and reciprocally mGluR6 upon LRIT1 immunoprecipitation (Figure 1E). Together, these findings establish LRIT1 as a binding partner of mGluR6.

### LRIT1 Is a Synaptic Protein Expressed in Both Photoreceptors and ON-BCs

**Figure 2:**
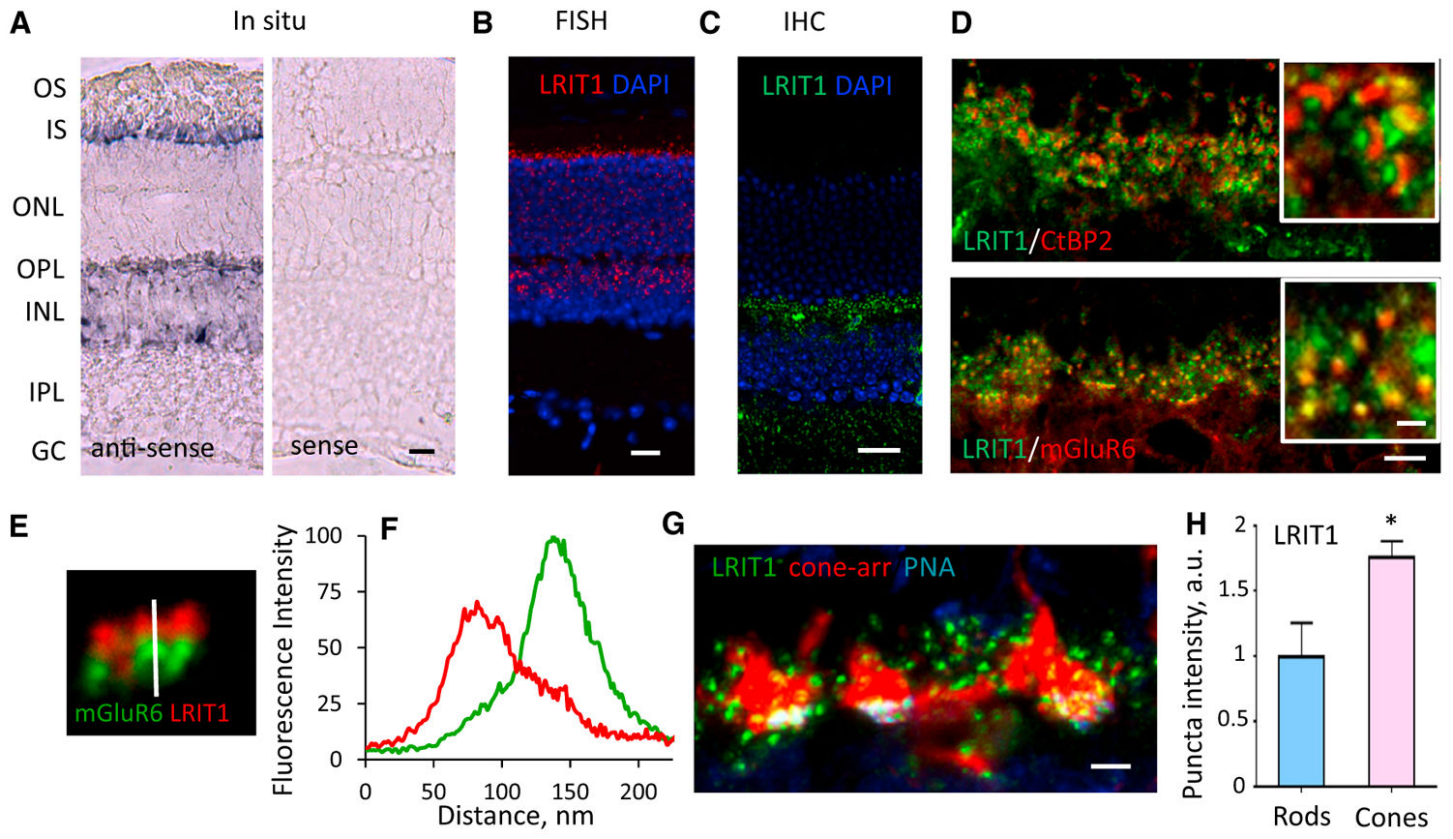
Characterization of LRIT1 Expression and Localization in the Retina (A) Traditional *in situ* hybridization for LRIT1 detection. Anti-sense or sense (negative control) probes derived from the LRIT1 sequence were used to probe retina cross-sections. Specific signal is revealed in both photoreceptor and bipolar cell layers. Scale bar, 25 μm. (B) High-resolution fluorescence *in situ* hybridization for *Lrit1* expression. Specific signal is detected in individual photoreceptors and bipolar cells. Scale bar, 20 μm. (C) Immunohistochemical detection of LRIT1 protein expression by staining retina cross-sections with anti-LRIT1 antibodies. The signal is confined to the outer plexiform layer (OPL). Scale bar, 20 μm. (D) Localization of LRIT1 at photoreceptor synapses revealed by co-immunostaining with pre-synaptic marker CtBP2 and postsynaptic marker mGluR6. Scale bar, 5 μm. Insets show higher magnification, scale bar, 1 μm. (E) High magnification for LRIT1 localization in synaptic puncta relative to mGluR6 by co-immunostaining of retina cross-section with the indicated antibodies. Vertical bar shows the scan line. (F) Quantification of LRIT1 distribution across synaptic puncta relative to mGluR6 determined by scanning fluorescence intensity along the line in (E). (G) Localization of LRIT1 in synapses of rod and cone photoreceptors. Cone pedicles were labeled by cone arrestin staining (red) along with the cone-specific active zone marker PNA used to determine selective localization of LRIT1 in cone synapses. Puncta outside of PNA/b-arrestin mask were considered to be rod synapses. Scale bar, 2.5 μm. (H) Quantification of LRIT1 content in rod and cone synapses by determining fluorescence intensities in respective puncta identified as in (G). Two sections from each retina, two retinas per genotype; *p < 0.05; t test.

We studied further LRIT1 expression and localization in the mouse retina. First, we performed *in situ* hybridization with anti-sense probes complementary to *Lrit1* mRNA and detected signals in layers occupied by photoreceptors and bipolar cells (Figure 2A). The signal was absent when the sense probe was used, demonstrating the specificity of hybridization. This result was confirmed by using a higher resolution and sensitivity *in situ* hybridization approach with fluorescence probes, where again we found *Lrit1* mRNA to be present in both photoreceptors and bipolar cells (Figure 2B). Next, we probed the localization of LRIT1 in retinal cross-sections by immunohistochemistry using anti-LRIT1 antibodies. These studies revealed the nearly exclusive presence of LRIT1 in the outer plexiform layer (OPL) that contains photoreceptor-to-bipolar cell synapses (Figure 2C). Detailed examination of the OPL showed that LRIT1 immunoreactivity is confined to characteristic puncta in close apposition to both the photoreceptor synaptic ribbons, as judged by co-staining with CtBP2 (Ribeye), and dendritic tips of ON-BCs, identified by co-staining with mGluR6 (Figure 2D). Higher power analysis followed by fluorescence line-scan intensity showed only partial overlap of LRIT1 with mGluR6, consistent with the presence of the LRIT1 in the synaptic cleft (Figures 2E and 2F). We further examined the cell-type specificity of LRIT1 expression and found it to be present at synapses of both rod and cone photoreceptors (Figure 2G). Quantitative analysis revealed its enrichment in the active zones of cone axonal terminals compared to rods suggesting that it might play a more prominent role in cone synaptic connectivity and/or function (Figure 2H). Overall, these data indicate that LRIT1 is produced by both rod and cone photoreceptors and ON-bipolar cells and is transported to the synapse where it is prominently present in the cone synaptic cleft.

### Synaptic LRIT1 Content Is Modulated by Changes in Photoreceptor Activity

**Figure 3:**
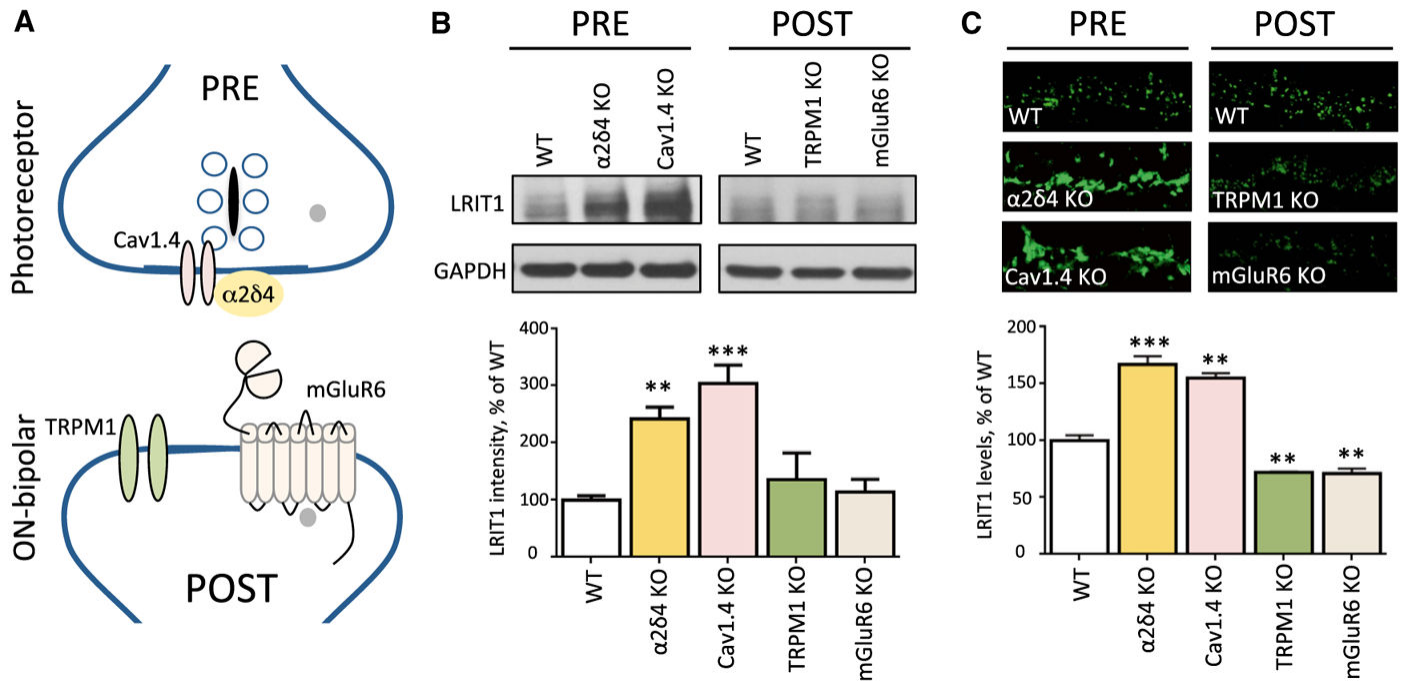
LRIT1 Synaptic Content Is Regulated by Changes in Presynaptic Release Apparatus (A) Scheme of the molecular organization of the photoreceptor synapse. Knockout mice lacking pre- and post-synaptic players depicted on the scheme were analyzed in the experiments. (B) Analysis of LRIT1 protein expression by western blotting in total lysates prepared from retinas of the respective mouse strains. Retinas from 3–5 mice for each genotype were used for the quantification of the LRIT1 band intensities and the values were normalized to WT. **p < 0.01, ***p < 0.001; t test. (C) Analysis of LRIT1 synaptic targeting by immunohistochemical staining of retina cross-sections of knockout mouse retinas as indicated. OPL regions are shown. Scale bar, 10 μm. The intensity of LRIT1 signal in the OPL was quantified and normalized to WT values. Two sections from each retina, two retinas per genotype; **p < 0.01, ***p < 0.001; t test.

To assess the contribution of pre- and post-synaptic compartments to LRIT1 expression and localization, we examined several mouse models with deletions in key components of the photoreceptor pre-synaptic release machinery or the post-synaptic signaling complex in ON-BCs (Figure 3A). Analysis of LRIT1 expression in the retinas by western blotting revealed that the elimination of either the neurotransmitter receptor mGluR6, or the ON-BC effector channel TRPM1 has no effect on LRIT1 expression. In contrast, the knockout of Ca_V_1.4 or α2δ4, which mediates the coupling of light-induced changes in membrane potential to glutamate release and synapse morphogenesis, respectively, results in a dramatic elevation of LRIT1 expression (Figure 3B). We further examined LRIT1 modulation at synapses by immunohistochemical staining of retinal cross-sections with anti-LRIT1 antibodies (Figure 3C). The results revealed a massive induction of LRIT1 content that occurs specifically at synapses where it is accumulated following deletion of pre-synaptic components: Ca_V_1.4 and α2δ4. In contrast, the deletion of the postsynaptic mGluR6 or TRPM1 resulted only in a minor downregulation of LRIT1 in the OPL. These findings suggest that LRIT1 expression and synaptic accumulation is inversely dependent on the neurotransmitter release orchestrated by the Ca_V_1.4 complex.

### Elimination of LRIT1 Does Not Affect Structural or Molecular Architecture of Photoreceptor Synapses

**Figure 4:**
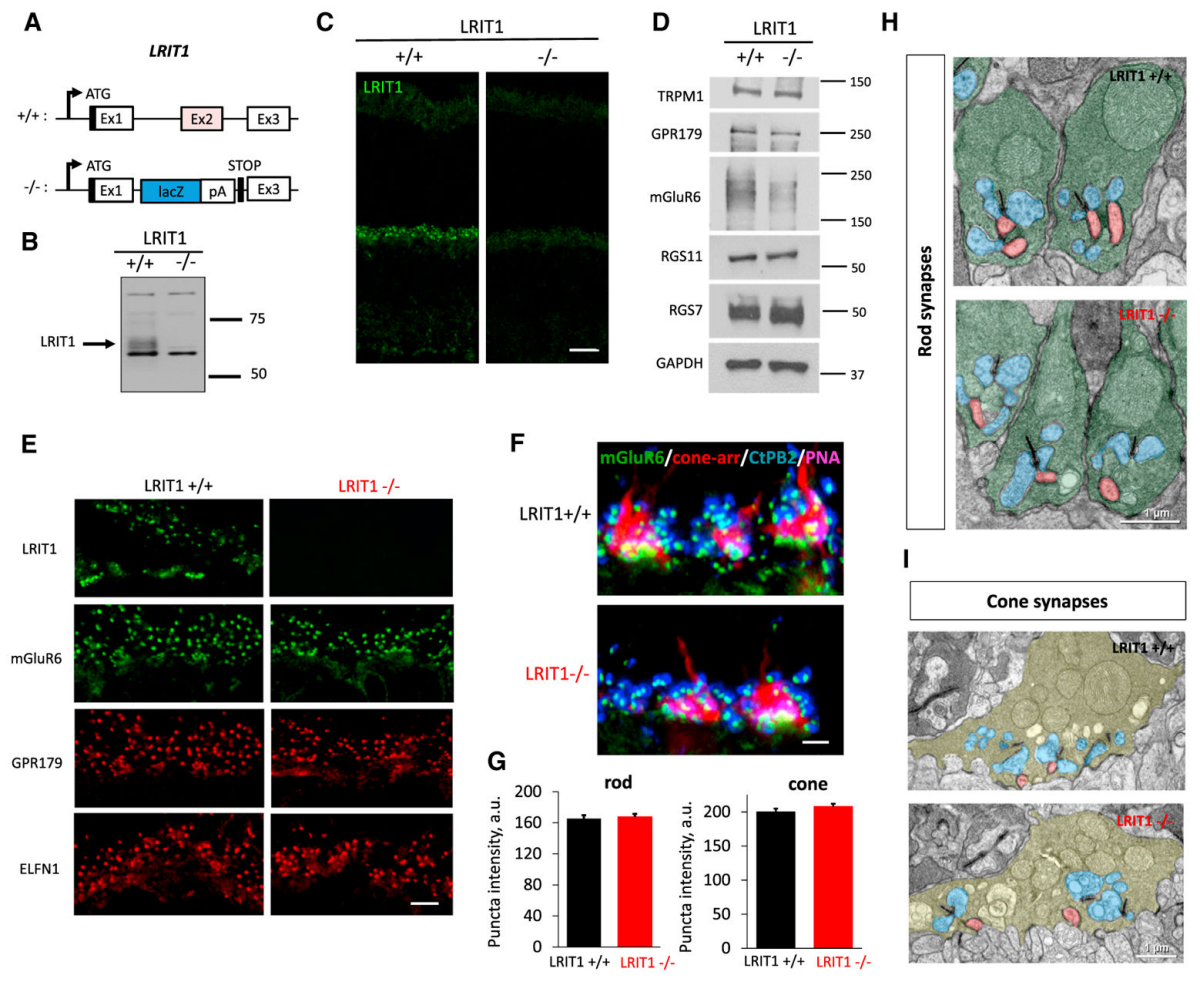
Generation and Characterization of Lrit1 Knockout Mice (A) Scheme for targeting *Lrit1* gene. The deletion strategy included elimination of the critical coding exon 2 and introduction of the premature stop-codon preceding exon 3. (B) Analysis of LRIT1 expression in wild-type and *Lrit1* knockout (−/−) mouse retinas by western blotting. (C) Analysis of LRIT1 localization in wild-type and *Lrit1* knockout (−/−) mouse retinas by immunohistochemical staining of retina cross-sections, scale bar, 20 μm. (D) Analysis of expression of proteins present in photoreceptor synapses by western blotting comparing wild-type and *Lrit1* knockout (−/−) mouse retinas. (E) Analysis of distribution of proteins present in photoreceptor synapses by immunohistochemical staining of retina cross-sections of wild-type and *Lrit1* knockout (−/−) mouse retinas. OPL regions are shown, scale bar, 5 μm. (F) Analysis of mGluR6 content in rod and cone synapses by immunohistochemistry. Staining with cone arrestin was used to define cone terminals and with PNA to identify active zones in the cone axons. Scale bar, 2.5 μm. (G) Quantification of changes in mGluR6 staining in rod and cone synapses in the retinas of wild-type and *Lrit1* knockout (−/−) mice. (H) Analysis of rod synapse morphology by electron microscopy. Rod terminals are labeled in green, horizontal cell processes in blue and ON-bipolar dendrites in red. (I) Analysis of cone synapse morphology by electron microscopy. Cone terminals are labeled in pale green, horizontal cell processes in blue and bipolar dendrites in red.

To determine the role of LRIT1 in the retina, we obtained *Lrit1* knockout mice (*Lrit1*^−/−^). In this line, the *Lrit1* allele is disrupted by placing a LacZ-Stop trap cassette immediately downstream of the first coding exon, thereby preventing translation of most of the *Lrit1* sequence (Figure 4A). Indeed, western blotting of whole retina lysates showed elimination of a specific band corresponding to LRIT1, indicating a complete ablation of LRIT1 protein (Figure 4B). Consistent with its transmembrane nature, the LRIT1 band was eliminated in knockout retinas and was concentrated in the membrane fraction while not present in the cytosol. In contrast, the major contaminating band was extracted in the soluble fraction, further confirming the specificity of assigning LRIT1 immunoreactivity (Figure S3). Immunohistochemical analysis showed elimination of immunostaining in the outer plexiform layer (OPL), additionally confirming the specificity of the antibodies (Figure 4C).

We found that deletion of LRIT1 did not affect overall morphology of the retina, its viability, or its laminar organization up to 3 months of age (Figures 4C and S1). Western blotting of *Lrit1*^−/−^ retina lysates revealed no significant changes in the expression of key components of synaptic signaling between photoreceptors and ON-BCs. Furthermore, we found no changes in synaptic targeting of molecules involved in synaptic transmission (mGluR6, GPR179, Ca_V_1.4, α2δ4) or the formation/maintenance of rod and cone synapses (ELFN1, LRIT3, connexin 36) (Figures 4E and S4). Detailed quantitative examination showed no changes in levels of mGluR6 in apposition to active zones in rod or cone synaptic terminals (Figures 4F and 4G).

We further studied the fine synaptic morphology by transmission electron microscopy and found no obvious evidence for structural abnormalities. Rod spherules in *Lrit1*^−/−^ retinas displayed normal shape and contained the expected elements, including the synaptic ribbon and the invaginating processes of horizontal cells and rod ON-BCs in direct apposition to synaptic ribbon (Figure 4H). Similarly, cone pedicles contained multiple ribbons and displayed clearly identifiable contacts with both horizontal cells and ON-BCs (Figure 4I). We thus conclude that deletion of LRIT1 had no major effect on the structural or molecular architecture of photoreceptor synapses.

### Ablation of LRIT1 Causes Selective Deficits In Background Adaptation Of Cone Synaptic Signaling

**Figure 5:**
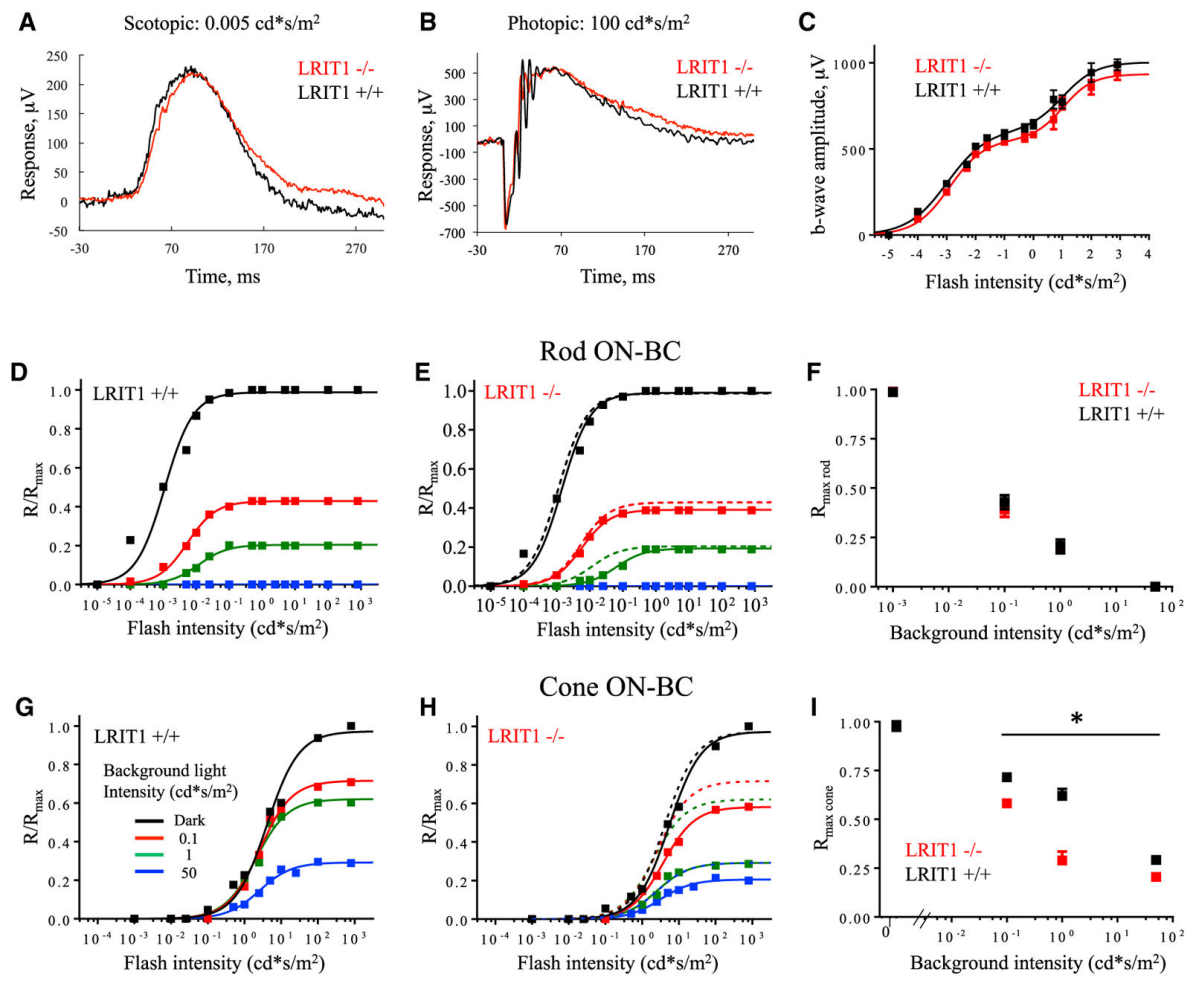
Analysis of LRIT1 Knockout by Electroretinography (A) Representative electroretinography (ERG) waveform recorded from dark-adapted mice in response to scotopic flash of light. (B) Representative ERG waveform recorded from dark-adapted mice in response to photopic flash of light. (C) Light-dependence profile of b-wave amplitudes. 4–6 mice were used for each genotype. (D) Rod-driven component of the b-wave across light intensities recorded in wild-type mice under dark-adapted conditions and various levels of background light. The first phase of the response at scotopic light intensities in (C) is shown. (E) Rod-driven component of the b-wave across light intensities recorded in *Lrit1* knockout mice under dark-adapted conditions and various levels of background light. Dashed lines represent superimposed fits from WT in (D). (F) Normalized changes in maximal amplitude of the rod-driven b-wave as a function of background light intensity recorded in both genotypes. (G) Cone-driven component of the b-wave across light intensities recorded in wild-type mice under dark-adapted conditions and various levels of background light. The second phase of the response at photopic light intensities in (C) is shown. (H) Cone-driven component of the b-wave across light intensities recorded in *Lrit1* knockout mice under dark-adapted conditions and various levels of background light. Dashed lines represent superimposed fits from wild-type mice in (G). (I) Normalized changes in maximal amplitude of the cone-driven b-wave as a function of background light intensity recorded in both genotypes.

We sought to determine a functional role for LRIT1 in light reception. Probing light-evoked responses of dark-adapted mice by electroretinography (ERG) revealed that *Lrit1*^−/−^ mice displayed a normal ERG waveform; a-wave and b-wave components were indistinguishable from wild-type littermates under both scotopic (Figure 5A) and photopic (Figure 5B) light regimes that activate rods and cones, respectively. Quantitative analysis revealed no changes in the maximal ERG b-wave amplitude in either the rod or cone-driven components (Figure 5C), indicating no gross abnormality in synaptic transmission to ON-BCs in dark-adapted mice. Single-cell recordings from rod ON-BCs and cone photoreceptor corroborated this observation. Voltage clamp (V_m_ = −60 mV) recordings from *Lrit1*^−/−^ and wild-type rod ON-BCs confirm the normal sensitivity of rod phototransduction and synaptic processing (Figure S2; Table S1). Furthermore, voltage clamp (V_m_ = −40 mV) recordings directly from cone photoreceptors revealed a robust maximum photocurrent with similar sensitivities and time courses in *Lrit1*^−/−^ and *Lrit1*^+/+^ retinas (Figure S2). We also observed no significant differences in the waveform or amplitudes of oscillatory potentials between the genotypes across the range of photopic light flashes, suggesting that LRIT1 ablation does not grossly affect processing of the visual signal by the inner retinal circuitry (Figure S5).

Next, we evaluated the role of LRIT1 in modulating the sensitivity of photoreceptor to ON-BC signaling during light adaptation. Consistent with previous reports, we found that increasing background light intensity reduced b-wave amplitudes elicited by both scotopic and photopic flashes. In the scotopic light intensity range, we observed no differences between genotypes over the range of background intensities (Figures 5D–5F), thought to drive solely rod-mediated responses. In contrast, when a rod-suppressing background was delivered, the cone-mediated b-waves displayed reduced amplitudes in *Lrit1*^−/−^ mice compared to their *Lrit1*^+/+^ littermates (Figures 5G–5I). Together, these observations suggest that elimination of LRIT1 impairs light adaptation for cone-driven signals in ON-BCs, resulting in a more pronounced suppression of the ON-BC response amplitude by background light.

### LRIT1 Controls the Sensitivity of Synaptic Transmission to Cone Bipolar Cells

**Figure 6:**
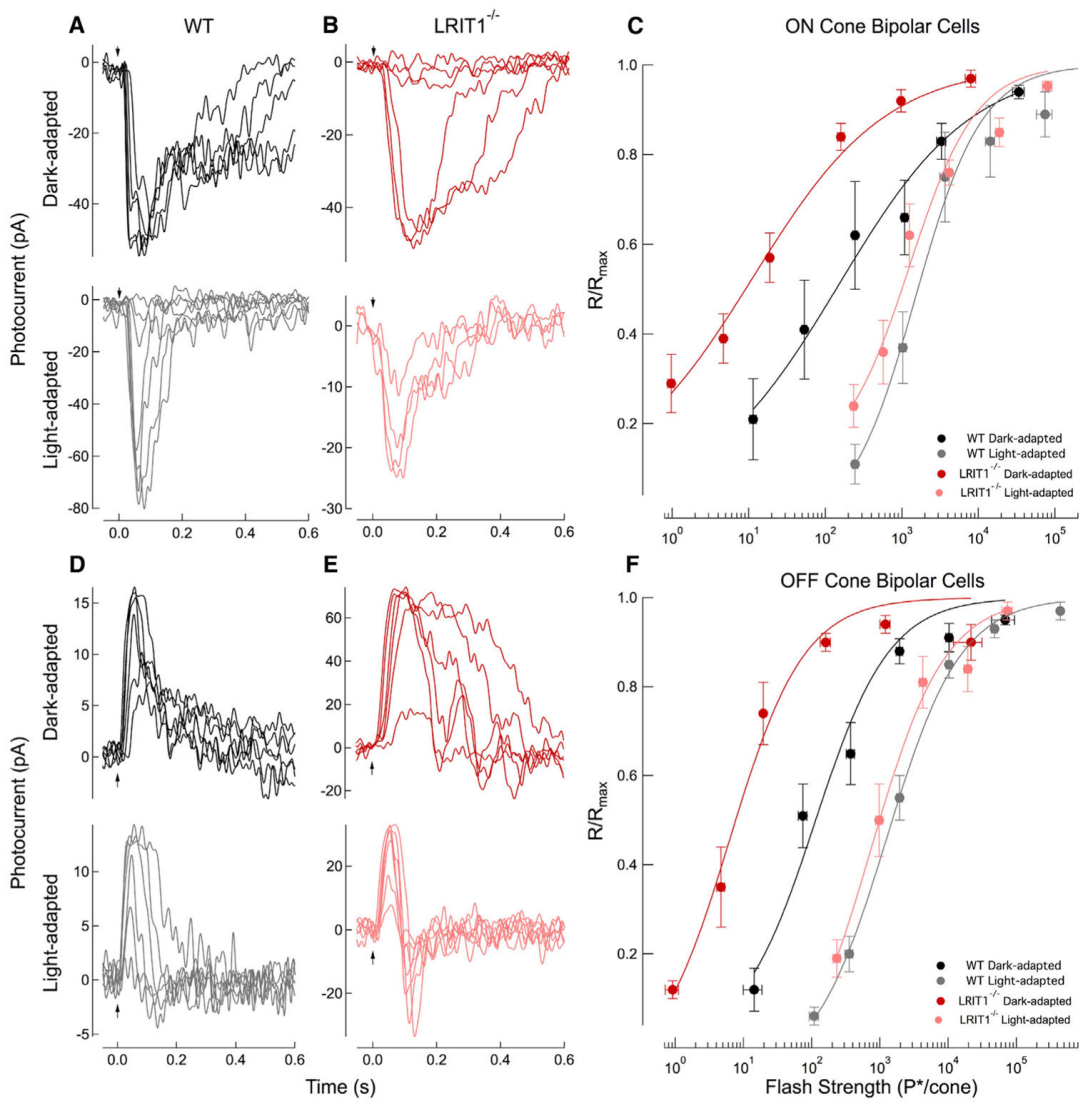
Analysis of Cone-to-BC Synaptic Transmission (A) Whole-cell voltage clamp recordings (V_m_ = −60 mV) were made from wild-type ON-BCs in retinal slices. Flash families were collected in the dark-adapted state and following the presentation of a background light that generated 2,100 P*/cone/s, where P* is an estimated number of activated cone pigments. The dark-adapted flash family was collected during the presentation of 10-ms flashes delivering 38, 69, 160, 560, and 2,000 P*/cone, whereas the family collected during the presentation of background light delivered 160, 560, 2,000, 7,700, 33,000, 160,000, and 560,000 P*/cone. Top and bottom panels are recordings made in sequence from the same cell. In light adaptation experiments, retinas were exposed to light for 2 min before recording. (B) Whole-cell voltage clamp recordings (V_m_ = −60 mV) from *Lrit1*^−/−^ ON-BCs. Flash families again were collected in the dark-adapted state and following the presentation of a background light that generated 2,100 P*/cone/s. The dark-adapted flash family was collected for 10-ms flashes delivering 0.7, 2.5, 9.5, 38, 130, and 560 P*/flash, whereas families collected during the presentation of background light generated 270, 1,100, 2,000, and 3,000 P*/flash. Top and bottom panels are recordings made in sequence from the same cell. (C) Response-intensity relationships for the dark-adapted and light-adapted WT and *Lrit1*^−/−^ ON-BC flash families. Population data were averaged across flash strengths and fit with a Hill Curve with an exponent of 0.5 for the dark-adapted families and 1.0 for the light-adapted families. The half-maximal flash strength (I_1/2_) for the fit was 150 P*/cone and 1,500 P*/cone for WT ON-BCs in the dark- and light-adapted states, respectively, a 10-fold shift. The half-maximal flash strength (I_1/2_) for the fit was 11 P*/cone and 1,300 P*/cone for *Lrit1*^−/−^ ON-BCs in the dark- and light-adapted states, respectively, a 120-fold shift. (D) Whole-cell voltage clamp recordings (V_m_ = −60 mV) were made from WT OFF-BCs in retinal slices. Flash families were collected in the dark-adapted state and following the presentation of a background light that generated 2,100 P*/cone/s. The dark-adapted flash family was collected during the presentation of 10-ms flashes delivering 37, 69, 560, 2,000, 7,700, and 33,000 P*/cone, whereas the family collected during the presentation of background light delivered 160, 560, 2,000, 33,000, 160,000, and 560,000 P*/cone. Top and bottom panels are recordings made in sequence from the same cell. (E) Whole-cell voltage clamp recordings (V_m_ = −60 mV) from *Lrit1*^−/−^ OFF-BCs. Flash families again were collected in the dark-adapted state and following the presentation of a background light that generated 2,100 P*/cone/s. The dark-adapted flash family was collected for 10-ms flashes delivering 7.0, 24, 40, 78, 640, and 2,300 P*/flash, whereas families collected during the presentation of background light generated 310, 640, 1,200, 2,300, 5,400, 12,000, and 55,000 P*/flash. Top and bottom panels are recordings made in sequence from the same cell. (F) Response-intensity relationships for the dark-adapted and light-adapted WT and *Lrit1*^−/−^ OFF-BC flash families. Population data were averaged across flash strengths and fit with a Hill Curve with an exponent of 0.8 for the dark-adapted and light-adapted families. The half-maximal flash strength (I_1/2_) for the fit was 110 P*/cone and 1,300 P*/cone for WT OFF-BCs in the dark- and light-adapted states, respectively, a 12-fold shift. The half-maximal flash strength (I_1/2_) for the fit was 6.3 P*/cone and 740 P*/cone for *Lrit1*^−/−^ OFF-BCs in the dark- and light-adapted states, respectively, a 120-fold shift.

To determine the mechanistic basis for the selectively impaired photopic ERG b-wave in background light, we measured the light-evoked responses of cone BCs in retinal slices. Recordings were made without a consideration of cone bipolar cell subtype, but appeared consistent across subtypes. Surprisingly, dark-adapted responses from *Lrit1*^−/−^ cone ON-BCs were ~10-fold more sensitive than their wild-type (WT) counterparts (Figures 6A and 6B), as determined based on the flash strength that yields a half-maximal response. Interestingly, ~10-fold increased sensitivity was also observed in dark-adapted responses from *Lrit1*^−/−^ cone OFF-BCs (Figures 6D and 6E), suggesting a common presynaptic origin of LRIT1 influence. Because the light intensities used to generate the flash families for *Lrit1*^−/−^ BCs activate rods only, but rod ON-BC responses remain unchanged compared to WT cells (Figure S2; Table S1), these data suggest that the absence of LRIT1 influences specifically synaptic communication between cones and cone BCs.

We probed further the mechanism underlying observed reduction of ERG b-wave amplitude by background light that suggests reduced capacity of *Lrit1*^−/−^ retinas for light adaptation. In the presence of a rod-suppressing background light delivering 2,100 P*/cone/s, flash families for cone ON- and OFF-BCs of both genotypes displayed a similar half-maximal flash strength (Figures 6A–6C; Table S1). Thus, the background light desensitized the *Lrit1*^−/−^ cone BCs to a much greater extent than wild-type cone BCs, effectively diminishing their difference in sensitivity. It should be stressed that response families recorded in background light were from the same cone BCs that the dark-adapted data were collected from, permitting formal analysis of the changes in sensitivity induced by background. When maximum response amplitude in this background light was considered, we additionally found that *Lrit1*^−/−^ ON-BCs displayed reduced amplitudes compared to their wild-type counterparts (Figures 6A and 6B; Table S1).

Thus, in addition to confirming deficits in light adaptation seen by ERG analysis, single-cell recordings further revealed an additional phenotype: increased dark-adapted sensitivity of ON an OFF cone BC light responses. This effect was not evident from the *en masse* analysis of neuronal responses to light by ERG likely due to large contributions by rod ON-BC activity, which effectively mask cone CB differences.

### LRIT1 Is Needed for Achieving High Temporal Resolution of Visual Discrimination

**Figure 7:**
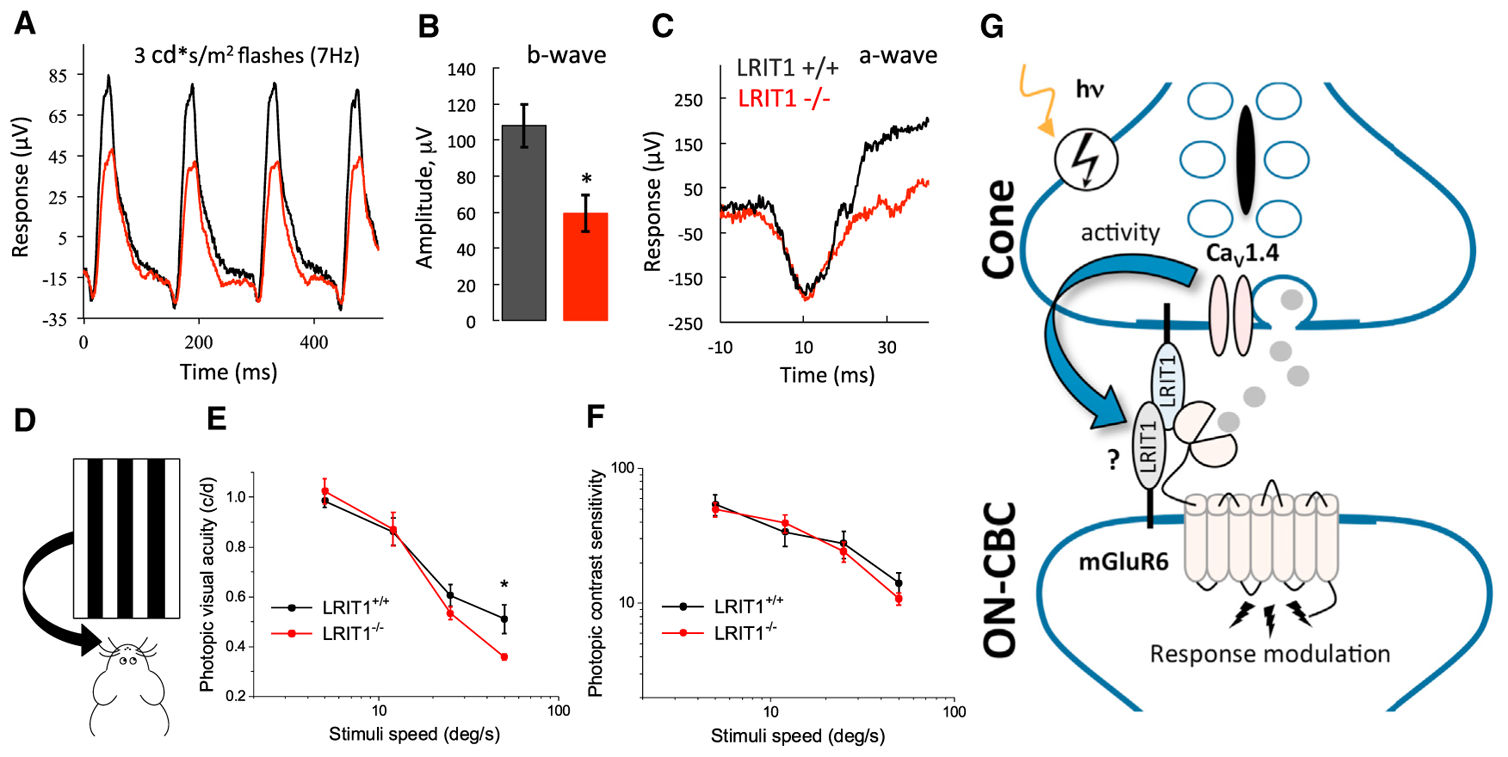
Visual Deficits in Mice Lacking LRIT1 (A) Representative flicker ERG traces in response to trains of stimulation delivered at 7 Hz frequency. (B) Quantification of b-wave amplitude changes recorded in flicker ERG protocol. *p < 0.05, t test, 4–6 mice per genotype. (C) Analysis of the a-wave recorded under flicker ERG protocol. (D) Scheme of the optokinetic reflex testing principle to assess visual function in mice. The ability of mice to track virtually moving grids with varying contrast, spatial, and temporal properties is recorded. (E) Dependence of photopic visual acuity on stimulation speed in the ORK test under 100% contrast of gratings. Deficits in photopic (light background of 1.1 cd s/m^2^) contrast sensitivity of *Lrit1* knockout mice are apparent only at highest stimuli speed of 50°/s (error bars are SEM; t test: *p < 0.05, n = 5–6). (F) Dependence of photopic contrast sensitivity on stimulation speed in the ORK test under constant light background of 1.1 cd/m^2^ (error bars are SEM; t test: *p < 0.05, n = 5–6). (G) Schematic representation of proposed role of LRIT1 in synaptic communication of cones. LRIT1 is expressed predominantly in cones and could also be present in cone BC with a possibility of forming *trans*-synaptic dimers given its capacity for heteromerization. It further interacts with postsynaptic mGluR6 receptor and presynaptic release apparatus containing Ca_V_1.4 complex to adjust neurotransmitter signaling at the synapse in response to light adaptation scaling synaptic transmission of cones. Changes in photoreceptor synaptic activity modulate LRIT1 levels further contributing to adaptation.

To further assess the functional role of diminished light adaptation in *Lrit1*^−/−^ retinas, we measured the capacity for the b-wave to track flickering light stimuli in the photopic light regime. Given the diminished cone-driven responses under background illumination, we tested processing of cone-derived signals in a flicker ERG paradigm that assesses mostly cone-mediated responses upon repeated stimulation, yet not fully eliminating rod contributions (Figure 7A). We found that under the conditions of this continuing light challenge, the *Lrit1* knockouts exhibited substantially reduced b-wave amplitudes consistent with deficits in background adaptation (Figures 7A and 7B). No genotype differences in the a-wave components of the flicker ERG were found, indicating normal rod and cone function (Figure 7C) and suggesting that the reduction in the b-wave is associated with changes in synaptic transmission to ON-BCs.

To understand the contribution of these observed adaptation deficits to vision, we evaluated behavior sensitivity in the optokinetic reflex (OKR) task on a steady light background (Figure 7D). Consistent with the reduction in the b-wave at the high-frequency stimulation, *Lrit1*^−/−^ mice showed reduced visual acuity (i.e., spatial resolution of stimuli presented at high temporal frequency) (Figure 7E). However, we found no significant differences in the ability of *Lrit1*^−/−^ mice to discriminate changes in contrast at any of the speed settings as compared to their wild-type littermates (Figure 7F). These observations suggest that loss of LRIT1 selectively compromises photopic visual acuity in a temporally challenging environment.

## DISCUSSION

The diversity of the neuronal cell types is thought to underlie the unique properties of individual circuits that collectively specialize to perform a vast range of computations enabling complex behaviors (Lodato and Arlotta, 2015; Zeng and Sanes, 2017). Such design requires not only specificity in wiring between distinct cell classes, but also that emergent properties are matched for the demands of the circuit (Bargmann and Marder, 2013; de Wit and Ghosh, 2016). Here, we provide support for the functional specialization of synaptic contacts for cone photoreceptor, but not rods. We propose that cones selectively rely on an adhesion molecule, LRIT1, for controlling scaling of their synaptic output (Figure 7G). Specifically, we found that LRIT1 is recruited to the active zones of both rod and cone photoreceptors where it is found in complexes involving the postsynaptic neurotransmitter receptor, mGluR6, on ON-BC dendrites. The deletion of LRIT1 did not affect the physical synapse assembly or molecular composition of synapses, yet it fundamentally changed their properties. Although LRIT1 is expressed in both rods and cones, we only detected effects at cone synapses. Remarkably, we found that knockout of LRIT1 increased the gain of synaptic transmission to ON and OFF bipolar cells, converting the sensitivity of cone ON-BCs to the range of rod ON-BCs. At the behavioral level, loss of LRIT1 compromised temporal aspects of photopic vision, although the exact mechanisms by which LRIT1 contributes to visual acuity remain to be established.

Despite the well documented role played by LRR proteins in synaptic structure (de Wit et al., 2011; Yogev and Shen, 2014), their influence on the functional aspects of signaling at metabotropic synapses have not been observed previously. We found that the augmentation of absolute sensitivity in cone BCs came at a price of reducing the ability of this synapse to adapt to continuous light exposure, thus limiting the operating range for transmitting signals. A key property of the cone system is the ability to operate over a wide range of background light, which they achieve by scaling their responsiveness with an increase in stimulation. Our findings demonstrate that LRIT1 is one of the molecular factors operating in cones that allow such adaptation at the level of controlling synaptic gain.

How can LRIT1 influence light sensitivity and synaptic scaling? Mechanistically, we think LRIT1 may exert its effects by several mechanisms, which are not necessarily mutually exclusive but remain to be established. We believe that the similar effects on the sensitivities of both ON and OFF CBs suggest that LRIT1 may act in photoreceptors to influence the presynaptic machinery that sets the rate of glutamate release. In line with this idea, we documented that the expression of LRIT1 is inversely dependent on the expression of the Ca_V_1.4 channel complex. Interestingly, we found that neither targeting nor accumulation levels of Ca_V_1.4 and α2δ4 was affected by LRIT1 elimination (Figure S4), suggesting that LRIT1 may influence glutamate release through fine-tuning the activity of the pre-synaptic calcium channel or other components of the release apparatus. Although we did not detect by EM any overt morphological changes precipitated by LRIT1 loss, some effects may also involve subtle structural changes in the synaptic cleft, including those related to positioning of active zone elements in relation to glutamate receptors that influence the efficiency of synaptic transmission.

Furthermore, physical interactions of LRIT1 with the principal neurotransmitter receptor mGluR6 may change the receptor signaling properties, perhaps by influencing the range of its responsiveness to glutamate. Such changes might affect the magnitude of the postsynaptic depolarization mediated by the downstream TRPM1 channel. These mechanisms may be further integrated together via *trans*-synaptic LRIT1 homomerization, suggested by the ability of LRIT1 to form dimers (Gomi et al., 2000). Although our *in situ* hybridization data indicate that LRIT1 messenger RNA is indeed present in both photoreceptors and bipolar neurons, the postsynaptic accumulation of LRIT1 in the dendritic tips of bipolar neurons is less certain at the protein level. Further experimentation will be required to establish the relevance of this dimerization model. LRIT1 can also have an additional role in regulating rod-cone coupling. Although we showed no significant change in connexin 36 content induced by LRIT1 loss, it is still possible that LRIT1 regulates cone ON-BCs sensitivity by suppressing rod-to-cone coupling through changes in gap junction efficiency rather than the connexin36 expression. Ultimately, a combination of these mechanisms is likely shaping LRIT1’s function and establishing their relative contributions will be an exciting future direction.

The discovery of LRIT1 as a key player in synaptic function of cones adds to a growing list of cell adhesion-like molecules that shape photoreceptor synapses. Interestingly, the expression of LRIT1 in photoreceptors was noted earlier (Gomi et al., 2000); however, it was reported to be localized to photoreceptor outer segments. We show clearly that LRIT1 is a synapse-specific protein located at both pre- and post-synaptic side of photoreceptor synapses using antibodies that we have validated against the *Lrit1*^−/−^ retina. The closest homolog of LRIT1 is LRIT3, another photoreceptor synaptic protein expressed by ON-bipolar neurons (Zeitz et al., 2013). LRIT3 is indispensable for the synaptic transmission for photoreceptor signals, as its inactivation in mice and humans leads to complete disruption of both rod and cone synaptic signaling (Neuillé et al., 2014, 2015; Qian et al., 2015). Additionally, LRIT3 appears to play a role in the morphogenesis of cone synaptic contacts with ON-BCs (Neuillé et al., 2015). The relationship between LRIT1 and LRIT3 is unclear but based on their similarity in domain composition, ~40% sequence identity and propensity of LRIT1 to dimerize, it seems possible that both molecules may work together to orchestrate molecularly similar processes.

In addition, two other leucine-rich repeat proteins with similar organization are present at the photoreceptor synapses. Rods specifically express the cell adhesion molecule ELFN1, which like LRIT1 forms complexes with mGluR6 and plays an essential role in physical assembly of the rod to rod ON-BC connections (Cao et al., 2015). Both rods and cones also rely on nyctalopin (NYX), which does not appear to be involved in synapse assembly but rather in photoreceptor synaptic signaling. NYX is expressed in both rod and cone ON-BCs where it was shown to play a role for the synaptic localization of the effector channel, TRPM1 (Cao et al., 2011; Pearring et al., 2011). Thus, it appears that photoreceptor synapses utilize a host of LRR molecules, possibly interwoven together, to coordinate synaptic assembly with synaptic function. Intriguingly, the cell adhesion molecules in this organization are further integrated with the components of the GPCR signaling cascade, pointing to higher level scaffolding and integration of morphogenic factors with the synaptic transmission machinery. Deciphering the molecular logic of this synaptic code at photoreceptor synapses will inform more generally how metabotropic synapses are specified and regulated.

## EXPERIMENTAL PROCEDURES

Detailed methods including reagents, mice strains, antibodies, procedures for western blotting, *in situ* hybridization, electroretinography (ERG), single-cell recordings, optokinetic testing (OKR) of vision in mice, immunoprecipitation, cell culture, appear in the Supplemental Experimental Procedures. When appropriate, statistical analyses were performed by employing Student’s t test with sample size of more than 3 independent biological replicates (mice), and the data are reported together with SEM values.

### Mice

Embryonic stem cell line with the *Lrit1*-targeted allele (Lrit1tm1a(EUCOMM) Hmgu) was obtained from EUCOMM (project 115689) and intended modifications described in the Results section were verified by sequencing and long range PCR. All studies involving mice were carried out in accordance with the NIH guidelines and were granted formal approval by the Institutional Animal Care and Use Committees.

### Antibodies and Western Blotting

The generation of the most antibodies was described previously. Rabbit anti-LRIT1 antibodies were generated against mouse recombinant LRIT1 (aa 549–624). Rabbit anti-LRIT3 CT antibody was generated against human recombinant LRIT3 (aa 604–679).

Whole retinas were removed from mice and lysed by sonication in ice-cold PBS supplemented with 150 mM NaCl, 1% Triton X-100, and Complete protease inhibitor tablets (Roche). Following sonication, lysates were cleared by centrifugation, subjected to 12.5% SDS/PAGE. Protein bands were transferred onto PVDF membranes and probed with antibodies.

### Cell Culture and Transfection

HEK293T cells were obtained from Clontech and cultured at 37°C and 5% CO_2_ in DMEM supplemented with antibiotics, 10% FBS. HEK293T cells were transfected at ~70% confluency using Lipofectamine LTX (Invitrogen) according to the protocol of the manufacturer. The cells were harvested processed for co-immunoprecipitation.

### Fluorescence In Situ Hybridization

The mRNA expression was evaluated with ViewRNATM 2-plex *In Situ* Hybridization Assay (Panomics, Santa Clara, CA) using the following probes: *Lrit1* (NM_146245.2; Cat# VB1-17470). 12-μm sections were post-fixed in 4% paraformaldehyde for 10 min, washed, and incubated with the probes.

### Immunohistochemistry

Dissected eyecups were fixed for 15 min in 4% paraformaldehyde, cryoprotected with 30% sucrose in PBS for 2 hr at room temperature, and embedded in optimal cutting temperature medium. 12-μm frozen sections were obtained and blocked in PT1 (PBS with 0.1% Triton X-100 and 10% donkey serum) for 1 hr then incubated with primary antibody in PT2 (PBS with 0.1% Triton X-100 and 2% donkey serum) for at least 1 hr. After four washes with PBS with 0.1% Triton, sections were incubated with fluorophore-conjugated secondary antibodies in PT2 for 1 hr. After four washes, sections were mounted in Fluoromount (Sigma).

### Electroretinography

Electroretinograms were recorded by using the UTA system and a Big-Shot Ganzfeld (LKC Technologies). Mice (~4–8 weeks old) were dark-adapted (≥6 hr) and prepared for recordings using a red dim light. ERG signals were sampled at 1 kHz and recorded with 0.3-Hz low-frequency and 300-Hz high-frequency cut-offs.

### Single-Cell Recordings

Light-evoked responses from photoreceptors and bipolar cells were recorded retinal slices using methods described previously (Okawa et al., 2010). Briefly, mice were dark-adapted overnight and euthanized according to protocols approved by the University of California, Los Angeles Animal Research Committee (Protocol 14-005-11). Slices were superfused with bicarbonate-buffered Ames media (equilibrated with 5% CO_2_/95% O_2_) heated to 35°C–37°C, visualized under infrared illumination, and stimulated with a blue light-emitting diode (λ_max_ ~405 nm). Light-evoked responses were measured using patch electrodes in voltage-clamp mode.

### Evaluation of Mouse Vision by Optokinetic Reflex Test

Photopic contrast sensitivity of mice was evaluated from optomotor responses using a two-alternative forced-choice protocol, as previously described (Kolesnikov et al., 2011; Umino et al., 2008).

## Supplementary Material

1

2

## Highlights

- LRIT1 is identified as a binding partner of mGluR6 complex in the retina
- LRIT1 accumulates at photoreceptor synapses in an activity-dependent manner
- Deletion of LRIT1 in mice increases the sensitivity of cone synaptic transmission
- Loss of LRIT1 impairs light adaptation of cone synapses and daylight vision
