# Supplementary material for: LRIT1 Modulates Adaptive Changes in Synaptic Communication of Cone Photoreceptors
Source: Cell Rep. Author manuscript; Available in PMC 2018 Apr 16. (PMC5902029; doi:10.1016/j.celrep.2018.03.008)
Supplement: 1 [file NIHMS957798-supplement-1.pdf]

Cell Reports, Volume *22*

Supplemental Information

LRIT1 Modulates Adaptive Changes in Synaptic Communication of Cone Photoreceptors

Ignacio Sarria, Yan Cao, Yuchen Wang, Norianne T. Ingram, Cesare Orlandi, Naomi Kamasawa, Alexander V. Kolesnikov, Johan Pahlberg, Vladimir J. Kefalov, Alapakkam P. Sampath, and Kirill A. Martemyanov

SUPPLEMENTAL INFORMATION

Supplemental Figures and Tables

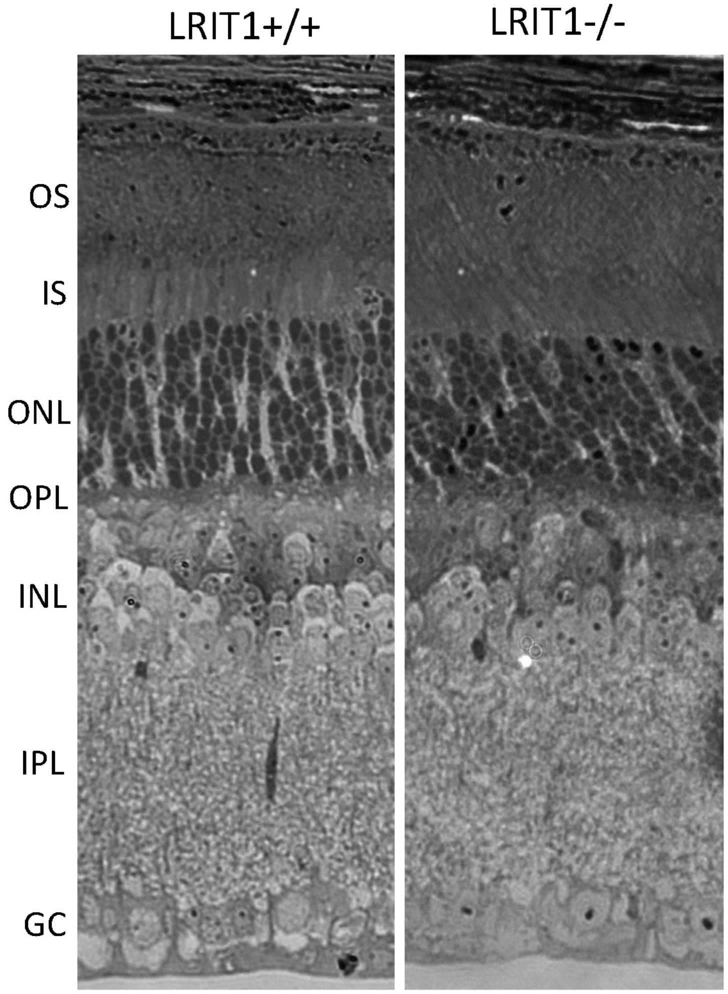

Figure S1. Related to Figure 4. Normal retina morphology in *Lrit1*$^{-/-}$ mice.
Analysis of the retina morphology by toluidine blue staining of ultra-thin (0.2 μm) retina cross-sections

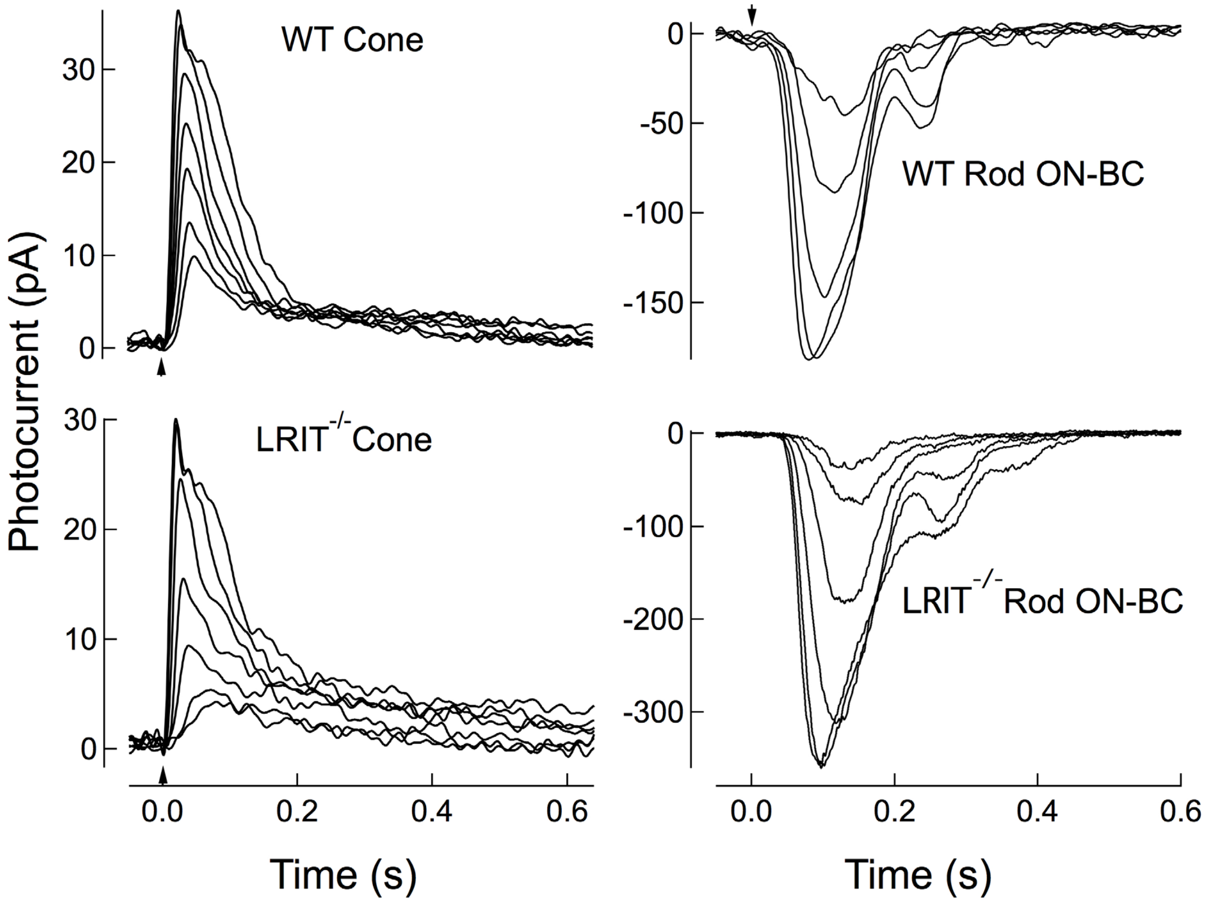

Figure S2. Related to Figure 6. Normal cone photoreceptor and rod ON-BC responses in *Lrit1*$^{-/-}$ mice.

Voltage-clamp (V_m = -40 mV) recordings from WT and *Lrit1*$^{-/-}$ cones. WT responses were evoked by 10ms flashes generating 350, 720, 1,400, 2,600, 4,500, 14,000, and 31,000 P*. *Lrit1*$^{-/-}$ responses were evoked by 10 ms flashes generating 70, 160, 560, 2,000, 7,700, 33,000, and 76,000 P*. Voltage-clamp (V_m = -60 mV) recordings from WT and *Lrit1*$^{-/-}$ rod ON-BCs. WT response were evoked by 10 ms flashes generating 1.5, 2.8, 6.6, 11, and 23 activated rhodopsins (R*). *Lrit1*$^{-/-}$ response were evoked by 10 ms flashes generating 1.2, 3.5, 5.9, 8.3, 13, and 20 R*.

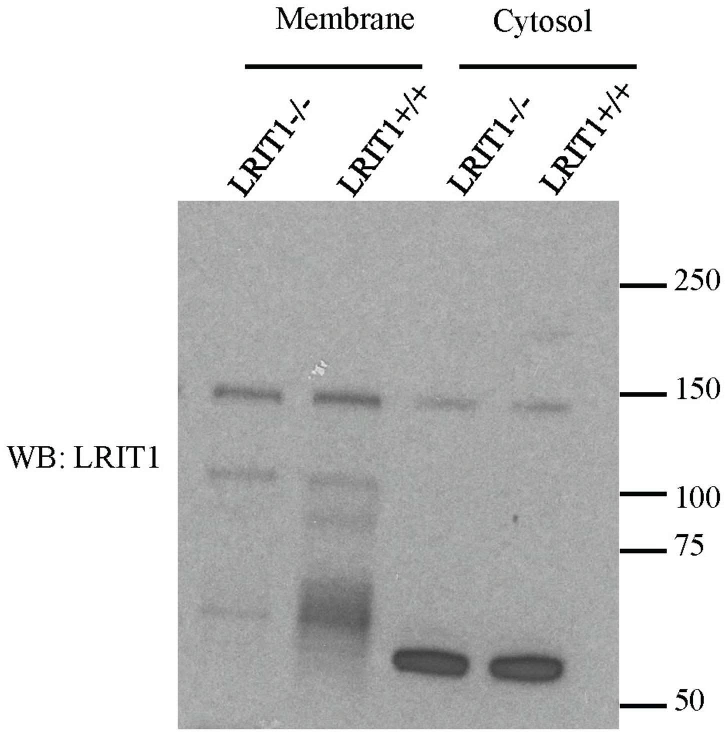

Figure S3. Related to Figure 4. Specific immunoreactive band for LRIT1 is associated with membranes

Western blotting analysis of LRIT1 using whole retina lysate from both wild-type (*Lrit1*$^{+/+}$) and *Lrit1*$^{-/-}$ mice after membrane fractionation. Equal amount of total protein from both cytosolic and membrane portion of each genotype was loaded and analyzed by western blot using specific LRIT1 antibody. Note that the intense band around 60kD detected by this antibody showed in cytosolic portion but not in membrane portion in both genotypes confirming its non-specificity.

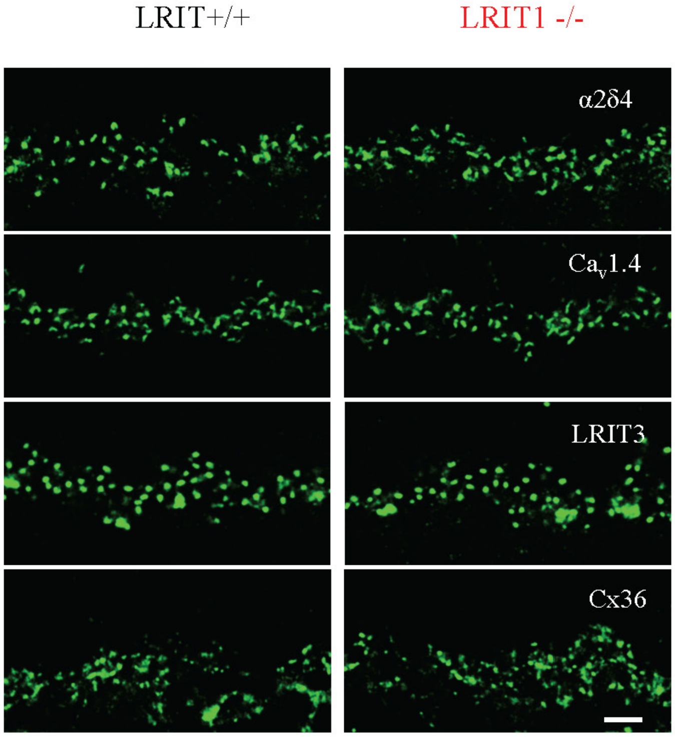

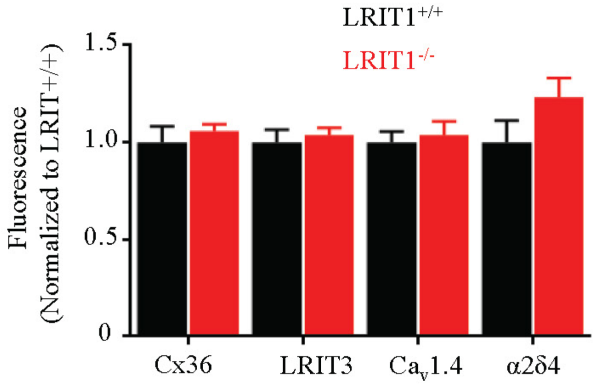

Figure S4. Related to Figure 4. The effect of *Lrit1* knockout on content of key synaptic proteins.

A, Representative confocal pictures of retina cross-sections from wild type (*Lrit1*$^{+/+}$) and LRIT1 knockout (*Lrit1*$^{-/-}$) mice stained with specific antibodies against different synaptic molecules as indicated. Scale bar: 20μm). OPL regions are shown. **B,** Quantification of the immunofluorescence intensities of synaptic molecules examined in panel A. Mean values were normalized to *Lrit1*$^{+/+}$ controls and plotted with corresponding SEMs.

A

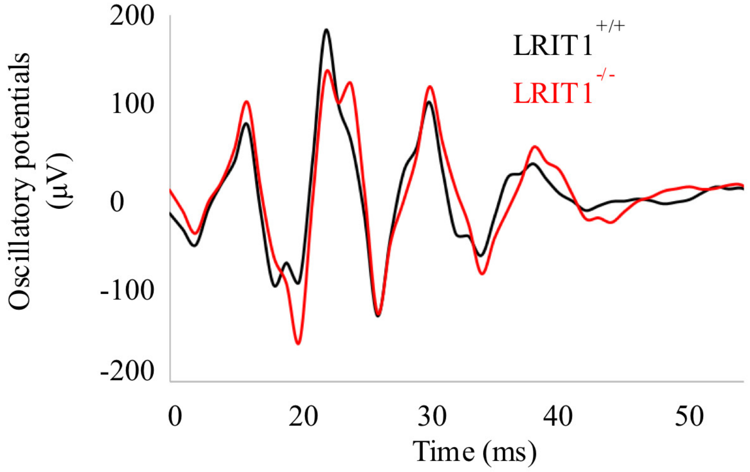

B

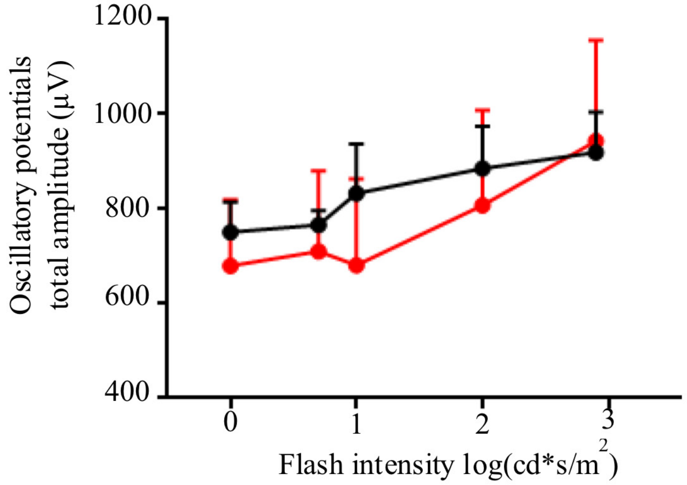

Figure S5. Related to Figure 5. Analysis of the oscillatory potentials of ERG recording.
A, Representative ERG traces of oscillatory potential components measured from dark-adapted mice stimulated with 100 cd/m^2 light. **B,** Statistical analysis of the oscillatory potential amplitudes at different intensities of light stimuli in *Lrit1*$^{+/+}$ and *Lrit1*$^{-/-}$ mice (Mean values and SEM were shown, multiple t-test, N=3 for *Lrit1*$^{+/+}$ and N=5 for *Lrit1*$^{-/-}$).

Table S1: Related to Figure 6. Response Characteristics of Photoreceptors and Bipolar Cells.

Cell Type		V_m (mV)	$I_{1/2}$ (P*/cone)	R_{max} (pA)
Cone Photoreceptor	WT	-37 ± 2.1 (13)	1800 ± 270 (20)	22 ± 1.9 (20)
	Lrit1$^{-/-}$	-33 ± 1.5 (7)	2600 ± 340 (8)	29 ± 1.4 (8)
Rod ON-BC** Dark-adapted	WT	-	4.8 ± 2.7 (2)	220 ± 41 (2)
	Lrit1$^{-/-}$	-	5.0 + 0.3 (5)	220 ± 51 (5)
Cone ON-BC Dark-adapted	WT	-	480 ± 92 (7)	70 ± 7.3 (7)
	Lrit1$^{-/-}$	-	28 ± 2.0 (22)	31 ± 1.0 (22)
Cone ON-BC 210 P*/cone/sec	WT	-	4700 ± 1600 (5)	48 ± 4.5 (5)*
	Lrit1$^{-/-}$	-	5000 ± 530 (14)	14 ± 0.88 (14)*
Cone OFF-BC Dark-adapted	WT	-	220 ± 110 (7)	34 ± 11 (7)
	Lrit1$^{-/-}$	-	9.5 ± 3.0 (7)	45 ± 10 (7)
Cone OFF-BC 210 P*/cone/sec	WT	-	1100 ± 320 (7)	26 ± 5.7 (7)
	Lrit1$^{-/-}$	-	1500 ± 560 (7)	27 ± 6 (7)

Data from (n) individual cells were first fit, then averaged. Response characteristics are documented as mean ± SEM (n)

* Denotes $p<0.05$ based on a paired student's t-test

** Note that rod ON-BC values for sensitivity are calculated in R*/rod

Supplemental Experimental Procedures

Mice

ES cell line with the *Lrit1* targeted allele (Lrit1tm1a(EUCOMM)Hmgu was obtained from EUCOMM (project 115689) and intended modifications described in the Results section were verified by sequencing and long range PCR. The ES cell line was used to generate chimeric mice by blastocyst injection at the Mouse Genetics core at the Scripps Research Institute. Resulting progeny was crossed with C57BL6 strain to establish germline transmission and the F1 mice were further crossed with the germline Cre-expressor strain B6.FVB-TgN(EIIa-Cre)C5379Lmgd (Jackson) to achieve elimination of exon 2 by LoxP recombination. The resulting constitutive *Lrit1* heterozygous knockout mice were inbred to produce -/- and +/+ littermates used in the study. Mice of both sexes were used in the experiments during daytime. Mice used in the study were 1–3 months old, and were maintained on a diurnal 12 h light/dark cycle. Procedures involving mice strictly followed NIH guidelines and were approved by the Institutional Animal Care and Use Committees at Scripps Florida, Washington University and the University of California, Los Angeles.

DNA constructs

Full-length cDNAs encoding human mGluR6 was purchased from Missouri S&T cDNA Resource Center (Cat# GRM6000000). Full-length cDNA encoding mouse *Lrit1* was purchased from Open Biosystems (Clone ID: 5401567). The C-terminal c-myc tagged mouse full length *Lrit1*, NT-LRIT1 (aa 1-527) were amplified from mouse *Lrit1* cDNA clone then sub-cloned into a pcDNA3.1/V5-His-TOPO (Invitrogen) expression vector according to manufacturer's specifications.

Antibodies and Western Blotting

The generation of the following antibodies was described previously: sheep anti-RGS11 (Cao et al., 2008), sheep anti-TRPM1 (Cao et al., 2011). Rabbit anti-RGS7 (7RC1), was a generous gift from William Simonds (NINDDK/NIH), and the guinea pig anti-mGluR6 antibody was a gift from Dr. Takahisa Furukawa (Osaka University). Rabbit anti-Cav1.4 antibody was a generous gift from Dr. Amy Lee (University of Iowa). Rabbit anti-LRIT1 antibodies were generated against mouse recombinant LRIT1 (aa 549-624). Rabbit anti-LRIT3 CT antibody was generated against human recombinant LRIT3 (aa 604-679). Rabbit anti-ELFN1 (NTR) and rabbit anti-ELFN1 (CTR) antibodies were generated against synthetic peptides of mouse ELFN1 (aa 305-320 and aa 530-547, respectively). Mouse anti-PKCα (ab11723; Abcam), mouse anti-CtBP2 (612044; BD Biosciences), mouse anti-Connexin36 (Clone 8F6.2, Millipore Bioscience Research Reagents, MAB3045), rabbit anti-α2δ4 (Aviva, OAAF04451) and mouse anti-GAPDH (Millipore; MAB374) were purchased.

Whole retinas were removed from mice and lysed by sonication in ice-cold PBS supplemented with 150 mM NaCl, 1% Triton X-100, and Complete protease inhibitor tablets (Roche). Lysates were cleared by centrifugation at 20,800 × *g* for 15 min at 4 °C. Total protein concentration in the supernatant was measured by using BCA Protein Assay Kit (Pierce). Supernatants were added with SDS sample buffer (pH 6.8) containing 8 M urea and were subjected to 12.5% SDS/PAGE. Protein bands were transferred onto PVDF membranes, subjected to Western blot analysis by using HRP-conjugated secondary antibodies, and detected by using ECL West Pico system (Pierce). Signals were captured on film and scanned by densitometer. For quantification, band intensities were determined by using NIH ImageJ software. Integrated intensity of GAPDH was used for data normalization.

Membrane fractionation was done as described in "Preparative immunoprecipitation of mGluR6 complexes" section.

Preparative immunoprecipitation of mGluR6 complexes from mouse retina and mass-spectrometry

Retinas were removed from mice and lysed by sonication in ice-cold PBS supplemented with 150 mM NaCl and Complete protease inhibitor tablets (Roche). After 30-minute centrifugation at 100,000 x g, 4 °C, the pellet was resuspended in ice-cold PBS IP buffer supplemented with 150 mM NaCl, 1% Triton X-100, and Complete protease inhibitor tablets (Roche). The membrane fraction was obtained by 30-minute incubation at 4 °C, and cleared by 30-minute centrifugation at 100,000 x g, 4 ° C, then subjected to immunoprecipitation as previously described (Cao et al., 2015). The beads were washed three times with ice-cold IP buffer. Proteins were eluted with 50 μL SDS sample buffer (62 mM Tris, 10% glycerol, 2% SDS, and 5% β-mercaptoethanol), entered SDS-PAGE by applying ~150 mV for 15-20 minutes. Gels were fixed with using 5% acetic acid in 50% methanol, stained by NOVEX colloidal blue (Invitrogen). Stained areas were cut out, digested with trypsin (Promega), and alkylated as described previously (Shevchenko et al., 2006). The resulting peptide mixtures were desalted, resolved by high-pressure liquid chromatography, and analyzed using LTQ-Orbitrap XL mass spectrometer, as described previously (Posokhova et al., 2011).

Cell culture and transfection

HEK293T cells were obtained from Clontech and cultured at 37°C and 5% CO_2 in DMEM supplemented with antibiotics, 10% FBS. HEK293T cells were transfected at ~70%

confluency using Lipofectamine LTX (Invitrogen) according to the protocol of the manufacturer. The cells were harvested and preceded to co-immunoprecipitation.

Immunoprecipitation

Cells or retina were lysed in ice-cold PBS IP buffer by sonication followed by centrifugation at 14,000 x g for 15 minutes. The supernatant was incubated with 20 µl of 50% protein G slurry (GE Healthcare) and 5 µg antibodies on a rocker at 4°C for 1 hour. After three washes with IP buffer, proteins were eluted from beads with 50 µl of SDS sample buffer. Proteins retained by the beads were analyzed with SDS-PAGE, followed by Western blotting using HRP conjugated secondary antibodies and an ECL West Pico (Thermo Scientific) detection system. Signals were captured on film and scanned by densitometer.

In situ hybridization

Eyeballs were dissected out and put into 4 % paraformaldehyde for 15 minutes. After incubated in 30% sucrose overnight, 12-µm retina sections were moved to OCT and cut using a Leica CM3050 S cryostat, rinsed in PBS and incubated for 90 minutes in hybridization solution (50% deionized formamide, 5X SSC, 5X Denhardt's solution, 500 µg/ml yeast tRNA, 500µg/ml sonicated salmon sperm DNA) at 50 °C. Each section was incubated overnight with hybridization solution at 55 °C containing the Dig-labeled riboprobes. On the second day, each section was washed three times with 0.5X SSC and 30% formamide at 55 °C for 10 minutes, followed by additional three-time washes with PBS at room temperature. The sections were incubated with blocking buffer (10% goat serum, 0.15M NaCl, 0.1M Tris-Cl pH7.5) for 1 hour at room temperature, followed by incubation with anti-dig-AP conjugate (1:500 in blocking buffer)

overnight at 4 °C. After three 10-minute washes with washing buffer (0.15M NaCl, 0.1M Tris-Cl pH7.5), the endogenous peroxidase was inactivated with buffer Developer Buffer (0.5 mg/mL levamisole, 100mM Tris-HCl pH 10, 50mM MgCl2, 100mM NaCl) for 5 minutes. The color was detected by incubation with Developer Buffer with 0.45 μl/mL NBT and 3.5 μl/mL BCIP for 30 minutes. Then the reaction was terminated by TE (pH8). The sections were mounted and images were acquired using an optical microscope (Leica DM IL LED).

Fluorescence *in situ* hybridization

The mRNA expression was evaluated with ViewRNATM 2-plex In Situ Hybridization Assay (Panomics, Santa Clara, CA) using the following probes: *Lrit1* (NM_146245.2; Cat# VB1-17470). The whole eye bulb was extracted, embedded in OCT and flash frozen in liquid nitrogen. 12 μm sections were cut using a Leica CM3050 S cryostat, rapidly post-fixed in 4% paraformaldehyde for 10 minutes, washed twice in phosphate buffered saline (PBS) and incubated for 2h in pre-hybridization mix (50% deionized formamide, 5X SSC, 5X Denhardt's solution, 250 μg/ml yeast tRNA, 500μg/ml sonicated salmon sperm DNA) at room temperature. Each section was incubated overnight with Panomics hybridization solution (using an incubator set to 40ºC, no CO_2 and humidity higher than 85%) containing the QuantiGene ViewRNA probe set diluted 1:50 in Probe Set Diluent QT. On the second day, the retina sections were processed according to manufacturer's instructions provided with the ViewRNA ISH Tissue Assay Kit (QVT0012). Briefly, sections were successively incubated with PreAmplifier Mix QT, Amplifier Mix QT, Label Probe 1-AP (1:1000), AP-Enhancer Solution and Fast Red Substrate. Finally, the nuclei were counterstained with DAPI and mounted using Fluoromont-G (SouthernBiotech). Confocal images were generated at The Light Microscopy Facility, the Max Planck Florida

Institute, using a LSM 780 Zeiss confocal microscope. Image acquisition and processing were accomplished using ZEN 2011 (64 bit) software (Carl Zeiss) with only minor manipulations of the images setting the fluorescence intensity in non-saturating conditions and maintaining similar parameters for each acquired image

Immunohistochemistry

Dissected eyecups were fixed for 15 min in 4% paraformaldehyde, cryoprotected with 30% sucrose in PBS for 2 h at room temperature, and embedded in optimal cutting temperature medium. Twelve-micrometer frozen sections were obtained and blocked in PT1 (PBS with 0.1% Triton X-100 and 10% donkey serum) for 1 h, then incubated with primary antibody in PT2 (PBS with 0.1% Triton X-100 and 2% donkey serum) for at least 1 h. After four washes with PBS with 0.1% Triton, sections were incubated with fluorophore-conjugated secondary antibodies in PT2 for 1 h. After four washes, sections were mounted in Fluoromount (Sigma).

For LRIT3 and α2δ4 staining, antigen retrieval was done by incubating slides in basic antigen retrieval reagent (R&D system) preheated to ~80 degree for 5 min before blocking. Images were taken with a Leica SP800 or Zeiss LSM 880 confocal microscope. Quantitative analysis of LRIT1, mGluR6, GPR179, and ELFN1 immunofluorescence from confocal images was performed using Leica software or Zen Blue 2 analysis software. Sections were double stained with for marker protein mGluR6, which localizes at the synaptic puncta of ON-BC and was used as a mask to define synapses. The fluorescence intensity within synaptic puncta was analyzed using constant puncta-encircling area, which tightly surrounded the contours of each puncta. A line of 1-1.5 μm (white bar) was drawn through the center of the distinct mGluR6-positive synapses and the distribution of the fluorescence intensity along this line was scanned to generate

the traces. Mean fluorescent intensity (measured in pixels) was averaged from ~10-20 individual and randomly selected mGluR6-positive puncta per imaged section. For LRIT1 immunofluorescence in different animal models and different synaptic molecules staining the entire OPL was first selected using hand drawn tool in Zen Blue and the mean immunofluorescent intensity within OPL was calculated by the software. The mean fluorescent intensity of two to three sections per retina, and two to three retinas per genotype were used for final quantification and comparison. Imaging parameters were the same for all sections and retinas.

Electroretinography (ERG)

Electroretinograms were recorded by using the UTA system and a Big-Shot Ganzfeld (LKC Technologies). Mice (~ 4-8 weeks old) were dark-adapted (≥6 h) and prepared for recordings using a red dim light. Mice were anesthetized with an i.p. injection of ketamine and xylazine mixture containing 100 and 10 mg/kg, respectively. All procedures were approved by the Institutional Animal Care and Use committee at the Scripps Florida Research Institute. Recordings were obtained from the right eye only, and the pupil was dilated with 2.5% phenylephrine hydrochloride (Bausch & Lomb), followed by the application of 0.5% methylcellulose. Recordings were performed with a gold loop electrode supplemented with contact lenses to keep the eyes immersed in solution. The reference electrode was a stainless steel needle electrode placed subcutaneously in the neck area. The mouse body temperature was maintained at 37 °C by using a heating pad controlled by ATC 1000 temperature controller (World Precision Instruments). ERG signals were sampled at 1 kHz and recorded with 0.3-Hz low-frequency and 300-Hz high-frequency cut-offs.

Full field white flashes were produced by a set of LEDs (duration < 5 ms) for flash strengths ≤ 2.5 cd*s/m^2 or by a Xenon light source for flashes > 2.5 cd*s/m^2 (flash duration < 5 ms). ERG responses were elicited by a series of flashes ranging from $1\times 10^{-5}$to 800 cd*s/m^2 in 10-fold increments. Ten trials were averaged for responses evoked by flashes up to 0.1 cd*s/m^2, and three trials were averaged for responses evoked by 0.5 and 1 cd*s/m^2 flashes. Single flash responses were recorded for brighter stimuli. To allow for recovery, interval times between single flashes were as follows: 5 s for 1×10^{-5} to 0.1 cd*s/m^2, 30 s for 0.5 and 1 cd*s/m^2, 60 s for 5 and 10 cd*s/m^2, and 180 s for 100 and 800 cd*s/m^2 flashes. Light backgrounds of 50, 1, and 0.1 cd/m^2 were administered for 5 minutes for recording partially saturated rod- and cone-only ERGs. At rod saturating (cone-only) backgrounds, ten trials were averaged at an interval recovery time of 1 second between flashes.

ERG traces were analyzed using the EM LKC Technologies software and Microsoft Excel. The b-wave amplitude was calculated from the bottom of the a-wave response to the peak of the b-wave. The data points from the b-wave stimulus–response curves were fitted by Equation 1 using the least-square fitting method in GraphPad Prism6.

(1) $$\mathbf{R=R_{max,r}*I/(I + I_{0.5,\,r}) + R_{max,c}*I/(I + I_{0.5,c})}$$

The first term of this equation describes rod-mediated responses (r), and the second term accounts primarily for responses that were cone mediated (usually at flash intensities ≥1 cd*s/m^2 for dark-adapted mice; index c). $R_{max,r}$ and $R_{max,c}$ are maximal response amplitudes, and $I_{0.5,r}$ and $I_{0.5,c}$ are the half-maximal flash intensities. Stimulus responses of retina cells increase in proportion to stimulus strength and then saturate, this is appropriately described by the hyperbolic curves of this function.

The oscillatory potential traces were generated by transforming the original ERG traces using the built-in Oscillatory Potential Analysis function in EM LKC Technologies software which also calculated the amplitude of oscillatory potential.

For the flicker ERG response test, 10 trials were averaged from 3cd*s/m^2 flashes at a delivery rate of 7 Hz.

Electron Microscopy

Eyes were enucleated, cleaned of extra-ocular tissue, and pre-fixed for 15 min in cacodylate-buffered half-Karnovsky's fixative containing 2mM calcium chloride. Then the eyecups were hemisected along the vertical meridian and fixed overnight in the same fixative. The specimens were rinsed with cacodylate buffer and postfixed in 2% osmium tetroxide in buffer for 1 hour, then gradually dehydrated in an increasing ethanol and acetone series (30–100%), and embedded in Durcupan ACM resin (Electron Microscopy Sciences, PA). Blocks were cut with 70-nm-thickness, and were stained with 3% lead citrate. Sections were examined in a Tecnai G2 spirit BioTwin (FEI) transmission electron microscope at 80 or 100 kV accelerating voltage. Images were captured with a Veleta CCD camera (Olympus) operated by TIA software (FEI).

Single cell recordings from cones and bipolar cells, and light calibrations

Light-evoked responses from photoreceptors and bipolar cells were recorded retinal slices using methods described previously (Okawa et al., 2010). Briefly, mice were dark-adapted overnight and euthanized according to protocols approved by the University of California, Los Angeles Animal Research Committee (Protocol 14-005-11). Eyes were enucleated under infrared

light, retinas were isolated, and 200-μm thick slices were cut with a vibrating microtome. Slices were superfused with bicarbonate-buffered Ames' media (equilibrated with 5% CO_2/95% O_2) heated to 35-37°C, visualized under infrared illumination, and were stimulated with a blue light-emitting diode (λ_{max} ~ 405nm).

Light-evoked responses were measured using patch electrodes in voltage-clamp mode (V_m = -40 mV for photoreceptor cells, V_m = -60 mV for bipolar cells), using an electrode internal solution consisting of (in mM): 125 K-aspartate, 10 KCl, 10 HEPES, 5 N-methyl-glucamine/HEDTA, 0.5 CaCl2, 1 ATP-Mg, and 0.2 GTP-Mg; pH was adjusted to 7.3 with N-methyl-glucamine hydroxide, and osmolarity was adjusted to 280 mOsm. Patch clamp recordings from cones additionally included 1 mM NADPH in the internal solution, which prevented response rundown. Light-evoked responses were sampled 10 kHz and filtered at 300 Hz with an 8-pole Bessel filter (Frequency Devices, Ottawa, IL). Data was further decimated and filtered offline at 50 Hz in Matlab (Mathworks, Natick, MA).

Recordings were made during experiments on WT and *Lrit1*$^{-/-}$ from cone photoreceptors, rod ON-BCs, and cone ON-BCs in retinal slices. The experimenter was blinded to the genotype of the animal until the recorded data was analyzed. We distinguished between bipolar cell types based on the polarity and time course of the response in conjunction with the cell's morphology. Recorded cells were visualized following their patch dialysis with a fluorophore (Alexa-750; Life Technologies) added to the electrode internal solution, allowing visualization in the far red without significant visual pigment bleaching. The responses of cones, and rod and cone ON-BCs were recorded from the same slices, and were typically adjacent to one another.

Light stimulation consisted of 10ms flashes of light that varied in strength from those yielding a just discernable response to those that generate a maximal response. Flash strengths are reported in activated cone pigment molecules (or P*/cone) at 405 nm, near the isosbestic point for S-cone and M-cone spectral sensitivities. To derive P*/cone, collecting areas were calculated from quantal responses at 405 nm recorded from control rods to derive the R*/rod. To calculate P*/cone, we adjusted this value for the difference in the volume of the cone vs. rod outer segment (cone/rod: $14\mu m^3/38\mu m^3$), which were then used to scale for the cone collecting area.

Evaluation of mouse vision by optokinetic reflex (OKR) test

Photopic contrast sensitivity of mice was evaluated from optomotor responses using a two-alternative forced-choice protocol, as previously described (Kolesnikov et al., 2011; Umino et al., 2008). Briefly, a mouse was placed on a pedestal surrounded by four computer monitors and observed from above using a camera. Mice responded to visual stimuli (sine-wave vertical gratings presented on the computer monitors using staircase paradigm and invisible to the experimenter), by reflexively rotating their head in either clockwise or counterclockwise direction. By looking at the very tip of animal's nose on a zoomed video view (140%), the observer registered the direction of optomotor responses and the computer determined the correctness of the choice (Prusky et al., 2004). In contrast to previous work (Kolesnikov et al., 2011; Umino et al., 2008), the duration of each trial was not strictly limited to 5 s, and the trial started only when the mouse was in a stable position on the pedestal, which could take up to several minutes. Photopic visual acuity was estimated as the threshold for spatial frequency of the stimuli at 100% contrast. Photopic contrast sensitivity was defined as the inverse of contrast threshold values which were obtained at fixed background luminance of monitors (1.1 cd m^{-2} at

the mouse eye level, as attenuated by neutral density film filters that formed a cylinder around the animal), over a range of various stimuli speeds (Sp), from 5 to 50 deg/s. Spatial frequency (Fs) of stimuli was kept constant at its optimal value of 0.128 cyc/deg, for a range of corresponding temporal frequencies (Ft = Sp*Fs) from 0.64 to 6.4 Hz (Umino et al., 2008). All data were analyzed using independent two-tailed Student *t*-test, with accepted significance level of $p < 0.05$.

Supplemental References

Cao, Y., Posokhova, E., and Martemyanov, K.A. (2011). TRPM1 forms complexes with nyctalopin in vivo and accumulates in postsynaptic compartment of ON-bipolar neurons in mGluR6-dependent manner. J Neurosci *31*, 11521-11526.

Cao, Y., Sarria, I., Fehlhaber, K.E., Kamasawa, N., Orlandi, C., James, K.N., Hazen, J.L., Gardner, M.R., Farzan, M., Lee, A., *et al.* (2015). Mechanism for Selective Synaptic Wiring of Rod Photoreceptors into the Retinal Circuitry and Its Role in Vision. Neuron *87*, 1248-1260.

Cao, Y., Song, H., Okawa, H., Sampath, A.P., Sokolov, M., and Martemyanov, K.A. (2008). Targeting of RGS7/Gbeta5 to the dendritic tips of ON-bipolar cells is independent of its association with membrane anchor R7BP. J Neurosci *28*, 10443-10449.

Kolesnikov, A.V., Rikimaru, L., Hennig, A.K., Lukasiewicz, P.D., Fliesler, S.J., Govardovskii, V.I., Kefalov, V.J., and Kisselev, O.G. (2011). G-protein betagamma-complex is crucial for efficient signal amplification in vision. J Neurosci *31*, 8067-8077.

Okawa, H., Miyagishima, K.J., Arman, A.C., Hurley, J.B., Field, G.D., and Sampath, A.P. (2010). Optimal processing of photoreceptor signals is required to maximize behavioural sensitivity. J Physiol *588*, 1947-1960.

Posokhova, E., Song, H., Belcastro, M., Higgins, L., Bigley, L.R., Michaud, N.A., Martemyanov, K.A., and Sokolov, M. (2011). Disruption of the Chaperonin containing TCP-1 function affects protein networks essential for rod outer segment morphogenesis and survival. Mol Cell Proteomics *10*, M110 000570.

Prusky, G.T., Alam, N.M., Beekman, S., and Douglas, R.M. (2004). Rapid quantification of adult and developing mouse spatial vision using a virtual optomotor system. Invest Ophthalmol Vis Sci *45*, 4611-4616.

Shevchenko, A., Tomas, H., Havlis, J., Olsen, J.V., and Mann, M. (2006). In-gel digestion for mass spectrometric characterization of proteins and proteomes. Nature protocols *1*, 2856-2860.

Umino, Y., Solessio, E., and Barlow, R.B. (2008). Speed, spatial, and temporal tuning of rod and cone vision in mouse. J Neurosci *28*, 189-198.
