# Supplementary material for: LRIT1 Modulates Adaptive Changes in Synaptic Communication of Cone Photoreceptors
Source: Cell Rep. Author manuscript; Available in PMC 2018 Apr 16. (PMC5902029; doi:10.1016/j.celrep.2018.03.008)
Supplement: 2 [file NIHMS957798-supplement-2.pdf]

Cell Reports

Article

LRIT1 Modulates Adaptive Changes in Synaptic Communication of Cone Photoreceptors

Graphical Abstract

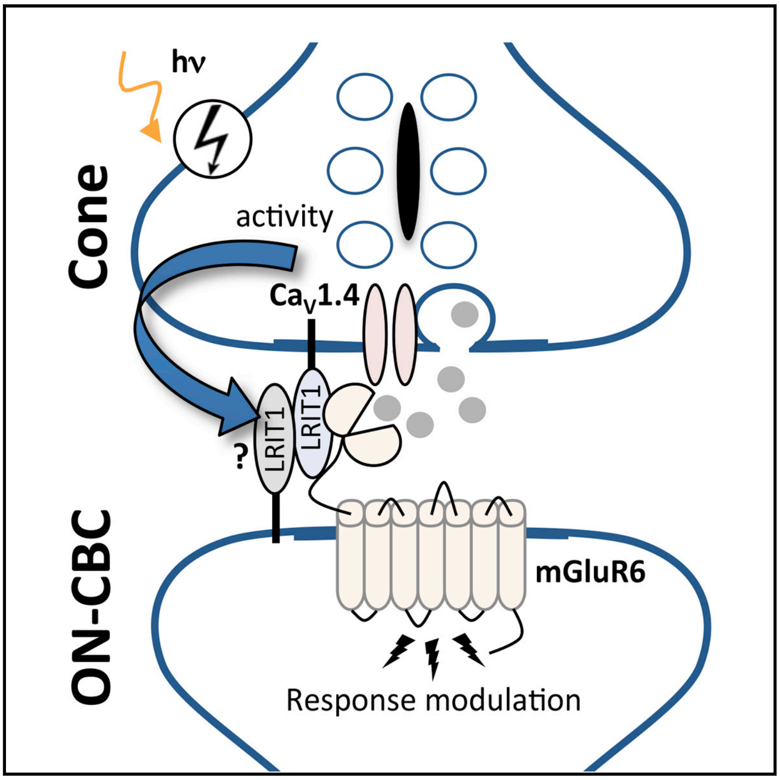

Highlights

- LRIT1 is identified as a binding partner of mGluR6 complex in the retina
- LRIT1 accumulates at photoreceptor synapses in an activity-dependent manner
- Deletion of LRIT1 in mice increases the sensitivity of cone synaptic transmission
- Loss of LRIT1 impairs light adaptation of cone synapses and daylight vision

Authors

Ignacio Sarria, Yan Cao, Yuchen Wang, ..., Vladimir J. Kefalov, Alapakkam P. Sampath, Kirill A. Martemyanov

Correspondence

kirill@scripps.edu

In Brief

Sarria et al. show LRIT1 accumulates at photoreceptors synapses. It forms a complex with a key receptor involved in processing neurotransmitter output of photoreceptors. They show that this protein plays an essential role in synaptic communication of cone photoreceptors and impacts daylight vision.

Sarria et al., 2018, Cell Reports *22*, 3562–3573
March 27, 2018 © 2018 The Author(s).
https://doi.org/10.1016/j.celrep.2018.03.008

CellPress

OPEN ACCESS
CellPress

Cell Reports
Article

LRIT1 Modulates Adaptive Changes in Synaptic Communication of Cone Photoreceptors

Ignacio Sarria,[1,5] Yan Cao,[1,5] Yuchen Wang,[1,5] Norianne T. Ingram,[2] Cesare Orlandi,[1] Naomi Kamasawa,[3] Alexander V. Kolesnikov,[4] Johan Pahlberg,[2] Vladimir J. Kefalov,[4] Alapakkam P. Sampath,[2] and Kirill A. Martemyanov[1,6,*]
[1]Department of Neuroscience, The Scripps Research Institute, Jupiter, FL 33458, USA
[2]Department of Ophthalmology, Stein Eye Institute, UCLA School of Medicine, Los Angeles, CA 90095, USA
[3]Electron Microscopy Core Facility, Max Planck Florida Institute, Jupiter, FL 33458, USA
[4]Department of Ophthalmology and Visual Sciences, Washington University School of Medicine, St. Louis, MO 63110, USA
[5]These authors contributed equally
[6]Lead Contact
*Correspondence: kirill@scripps.edu
https://doi.org/10.1016/j.celrep.2018.03.008

SUMMARY

Cone photoreceptors scale dynamically the sensitivity of responses to maintain responsiveness across wide range of changes in luminance. Synaptic changes contribute to this adaptation, but how this process is coordinated at the molecular level is poorly understood. Here, we report that a cell adhesion-like molecule, LRIT1, is enriched selectively at cone photoreceptor synapses where it engages in a *trans*-synaptic interaction with mGluR6, the principal receptor in postsynaptic ON-bipolar cells. The levels of LRIT1 are regulated by the neurotransmitter release apparatus that controls photoreceptor output. Knockout of LRIT1 in mice increases the sensitivity of cone synaptic signaling while impairing its ability to adapt to background light without overtly influencing the morphology or molecular composition of photoreceptor synapses. Accordingly, mice lacking LRIT1 show visual deficits under conditions requiring temporally challenging discrimination of visual signals in steady background light. These observations reveal molecular mechanisms involved in scaling synaptic communication in the retina.

INTRODUCTION

In the vertebrate retina, the rod and cone photoreceptors respond to incident light by modulating their membrane potential. This signal is transmitted to their bipolar cells and eventually to higher visual centers that enable our complex visual experience. Rods and cones subdivide the range of the visual system by mediating light reception in different regimes of intensity (Ingram et al., 2016; Pugh and Lamb, 2000; Yau and Hardie, 2009). Rods are exquisitely sensitive and are capable of detecting single photon absorptions, yet their responses are relatively slow and their dynamic range is limited. In contrast, cones are less sensitive, but faster, cover a wider range of light intensities, and are more resistant to saturation than rods (Burkhardt, 1994; Matthews et al., 1990; Nikonov et al., 2006). Furthermore, cones normally operate under daylight conditions where light intensities vary over a wide range, requiring them to adjust dynamically the sensitivity of their responses (Korenbrot, 2012; Soo et al., 2008; Stockman et al., 2006).

Photoreceptors use a variety of mechanisms to modulate their gain to maintain responsiveness as light intensities vary (Govardovskii et al., 2000; Morshedian and Fain, 2017; Pugh et al., 1999); one critical control point is at their synapse with bipolar cells (Wu, 1994). Light hyperpolarizes the photoreceptor membrane potential, which biases voltage-gated $Ca_V1.4$ Ca^{2+} channels in their axonal terminals toward the closed state, thereby reducing Ca^{2+} influx and glutamate release (Heidelberger et al., 2005; Joiner and Lee, 2015). Modulating neurotransmitter release through a combination of intrinsic mechanisms that control the release machinery (Thoreson et al., 2004; Yang and Wu, 1997) as well as through the negative feedback from the downstream neurons (Kramer and Davenport, 2015; Vroman et al., 2013) has been established as a powerful means for adjusting the gain of the photoreceptor synaptic output. However, the molecular mechanisms underlying this process, particularly as it varies between rods and cones, remain controversial and poorly understood. Even less clear is how photoreceptors coordinate the gain of the synaptic transmission with their dedicated postsynaptic bipolar cells, which are responsible for decoding photoreceptor signals.

In postsynaptic bipolar cell dendrites, the reduction in glutamate release is detected by two classes of bipolar cells; the OFF type neurons (OFF-BCs) that predominantly contact cones in the mammalian retina and utilize ionotropic glutamate receptors to preserve the hyperpolarizing photoreceptor light response, and the ON type (ON-BCs) that use metabotropic mGluR6 receptors to generate a depolarizing response that inverts the sign of the photoreceptor response. We now appreciate that there are at least 8 subtypes of ON-BCs showing functional specialization and selectivity in establishing contacts with either rods or cones (Euler et al., 2014; Hoon et al., 2014).

 © 2018 The Author(s).
This is an open access article under the CC BY license (http://creativecommons.org/licenses/by/4.0/).

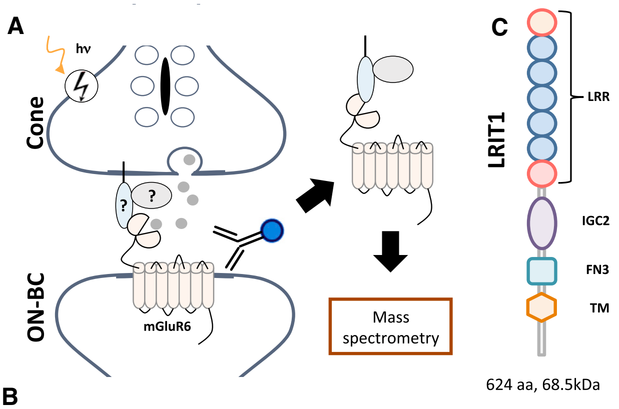

B

Peptide sequence	Precursor m/z	Spectrum charge	ΔPPM	Peptide identification probability	Mascot Ion score	Mascot Delta Ion Score
DTPQLQLLDLQANR	812.9359	2	-0.6782	95%	90.26	90.26
SLAGVAFSQLELR	695.8891	2	1.422	95%	44.35	44.35
TGEGAEAAAYNNK	648.2964	2	-0.5727	95%	76.62	76.62
TGGSAEASGAYVNLER	791.3812	2	3.059	95%	79.3	79.3

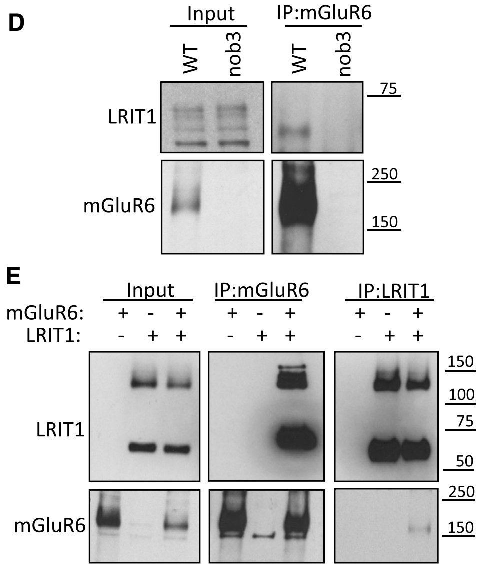

Figure 1. Identification of LRIT1 as mGluR6 Binding Partner

(A) Schematic of the affinity purification strategy for the identification of the mGluR6 binding partners of mGluR6 at photoreceptor synapses. Specific anti-mGluR6 antibodies were used for the immunoprecipitation from membrane fractions of the retina and the eluates were subjected to mass-spectrometry.

(B) Peptides matching to LRIT1 sequences identified in the mass-spectrometric experiments. Characteristics and parameters used for defining the sequences are shown.

(C) Domain organization of LRIT1. LRR, leucine reach repeat; IGC2, type 2 IgG-like domain; FN3, fibronectin type 3 domain; TM, transmembrane segment.

(D) Verification of mGluR6 interaction with LRIT1 by co-immunoprecipitation from retina lysates. Anti-mGluR6 antibodies were used for the immunoprecipitation (IP) and the presence of LRTI1 and mGluR6 in the IP eluates was detected by western blotting. Retinas lacking mGluR6 (nob3) were used as a specificity control.

(E) Characterization of mGluR6-LRIT1 interaction in transfected HEK293T cells. Both forward and reverse immunoprecipitation experiments using anti-LRIT1 and mGluR6 antibodies were conducted following expression of the indicated constructs and the proteins were detected by western blotting.

The core of the postsynaptic mGluR6 pathway that activates ON-BCs includes the heterotrimeric G protein $G\alpha_o\beta3\gamma13$, which in turn gates the effector ion channel TRPM1 (Martemyanov and Sampath, 2017; Morgans et al., 2010; Vardi et al., 2002). This signaling cascade is coordinated additionally by a host of proteins with critical roles in enabling synaptic transmission including regulator of G protein signaling (RGS) proteins, the orphan receptor GPR179, and leucine-rich repeat (LRR) proteins NYX and LRIT3, scaffolded together in a macromolecular complex (Gregg et al., 2014; Martemyanov and Sampath, 2017; Zeitz et al., 2015).

Recent findings suggest that the postsynaptic cascade of ON-BCs is further engaged in contacts with the photoreceptor presynaptic release apparatus (Cao et al., 2015; Tummala et al., 2016; Wang et al., 2017). For example, the mGluR6 is directly recruited by the rod-specific molecule ELFN1 to the $Ca_V1.4$ channel complex, an interaction that is crucial for the physical assembly of rod synapses and the transmission of rod signals to rod ON-BCs (Cao et al., 2015; Wang et al., 2017). No analogous interactions have yet been reported for the cone synapses. In addition, how these interactions influence the functional properties of synapses is not understood at any metabotropic synapse. Here, we report the identification of a leucine-rich repeat (LRR) protein, LRIT1, at photoreceptor synapses that binds *trans*-synaptically to mGluR6 and facilitates synaptic adaptations of cone photoreceptors upon changes in luminance.

RESULTS

Identification of LRIT1 As a Component of mGluR6 Complex

We have previously reported a screen for the mGluR6 binding partners by immunoprecipitation with specific anti-mGluR6 antibodies followed by mass-spectrometric identification of co-purified proteins present in the eluates. In this study, we focused on candidate cell-surface molecules with potential roles in cone synaptic function (Figure 1A). In this screen, we found 4 peptides with high identification confidence that map to the sequence of the transmembrane protein, LRIT1 (Figure 1B). This protein features multiple extracellular modules including leucine-rich repeats (LRR) and IgG-like and fibronectin type III domains (Figure 1C) and belongs to the extended family of cell-adhesion like proteins (de Wit and Ghosh, 2016). To validate the specificity of the interaction, we conducted mGluR6 immunoprecipitation from wild-type mouse retinas while in parallel using retinas from nob3 mice, lacking mGluR6. When probing blots with our anti-LRIT1 antibodies, we found a single band corresponding to the predicted size of LRIT1 protein in the eluates of wild-type but not *nob3* retinas, confirming the specificity of LRIT1-mGluR6 interaction in native retinas (Figure 1D). We then probed the binding in the reconstituted system. For this, HEK293 cells were co-transfected with various combinations of mGluR6 and LRIT1 followed by reciprocal immunoprecipitation experiments. Again, we detected the robust and specific

OPEN
ACCESS
CellPress

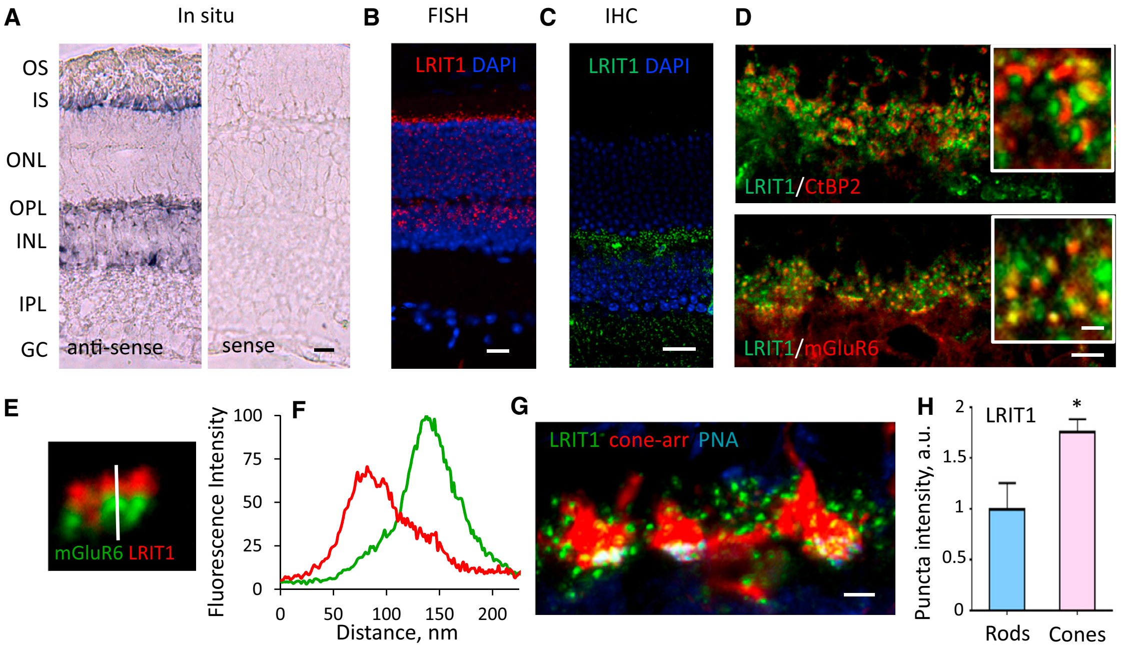

Figure 2. Characterization of LRIT1 Expression and Localization in the Retina

(A) Traditional *in situ* hybridization for LRIT1 detection. Anti-sense or sense (negative control) probes derived from the LRIT1 sequence were used to probe retina cross-sections. Specific signal is revealed in both photoreceptor and bipolar cell layers. Scale bar, 25 μm.

(B) High-resolution fluorescence *in situ* hybridization for *Lrit1* expression. Specific signal is detected in individual photoreceptors and bipolar cells. Scale bar, 20 μm.

(C) Immunohistochemical detection of LRIT1 protein expression by staining retina cross-sections with anti-LRIT1 antibodies. The signal is confined to the outer plexiform layer (OPL). Scale bar, 20 μm.

(D) Localization of LRIT1 at photoreceptor synapses revealed by co-immunostaining with pre-synaptic marker CtBP2 and postsynaptic marker mGluR6. Scale bar, 5 μm. Insets show higher magnification, scale bar, 1 μm.

(E) High magnification for LRIT1 localization in synaptic puncta relative to mGluR6 by co-immunostaining of retina cross-section with the indicated antibodies. Vertical bar shows the scan line.

(F) Quantification of LRIT1 distribution across synaptic puncta relative to mGluR6 determined by scanning fluorescence intensity along the line in (E).

(G) Localization of LRIT1 in synapses of rod and cone photoreceptors. Cone pedicles were labeled by cone arrestin staining (red) along with the cone-specific active zone marker PNA used to determine selective localization of LRIT1 in cone synapses. Puncta outside of PNA/b-arrestin mask were considered to be rod synapses. Scale bar, 2.5 μm.

(H) Quantification of LRIT1 content in rod and cone synapses by determining fluorescence intensities in respective puncta identified as in (G). Two sections from each retina, two retinas per genotype; $^*p < 0.05$; t test.

pull down of LRIT1 when mGluR6 was immunoprecipitated and reciprocally mGluR6 upon LRIT1 immunoprecipitation (Figure 1E). Together, these findings establish LRIT1 as a binding partner of mGluR6.

LRIT1 Is a Synaptic Protein Expressed in Both Photoreceptors and ON-BCs

We studied further LRIT1 expression and localization in the mouse retina. First, we performed *in situ* hybridization with anti-sense probes complementary to *Lrit1* mRNA and detected signals in layers occupied by photoreceptors and bipolar cells (Figure 2A). The signal was absent when the sense probe was used, demonstrating the specificity of hybridization. This result was confirmed by using a higher resolution and sensitivity *in situ* hybridization approach with fluorescence probes, where again we found *Lrit1* mRNA to be present in both photoreceptors and bipolar cells (Figure 2B). Next, we probed the localization of LRIT1 in retinal cross-sections by immunohistochemistry using anti-LRIT1 antibodies. These studies revealed the nearly exclusive presence of LRIT1 in the outer plexiform layer (OPL) that contains photoreceptor-to-bipolar cell synapses (Figure 2C). Detailed examination of the OPL showed that LRIT1 immunoreactivity is confined to characteristic puncta in close apposition to both the photoreceptor synaptic ribbons, as judged by co-staining with CtBP2 (Ribeye), and dendritic tips of ON-BCs, identified by co-staining with mGluR6 (Figure 2D). Higher power analysis followed by fluorescence line-scan intensity showed only partial overlap of LRIT1 with mGluR6, consistent with the presence of the LRIT1 in the synaptic cleft (Figures 2E and 2F). We further examined the cell-type specificity of LRIT1 expression and found it to be present at synapses of both rod and cone photoreceptors (Figure 2G). Quantitative analysis revealed its enrichment in the active zones of cone axonal terminals compared to rods suggesting that it might play a more prominent role in cone synaptic connectivity and/or function (Figure 2H). Overall, these data indicate that LRIT1 is produced by both rod and cone

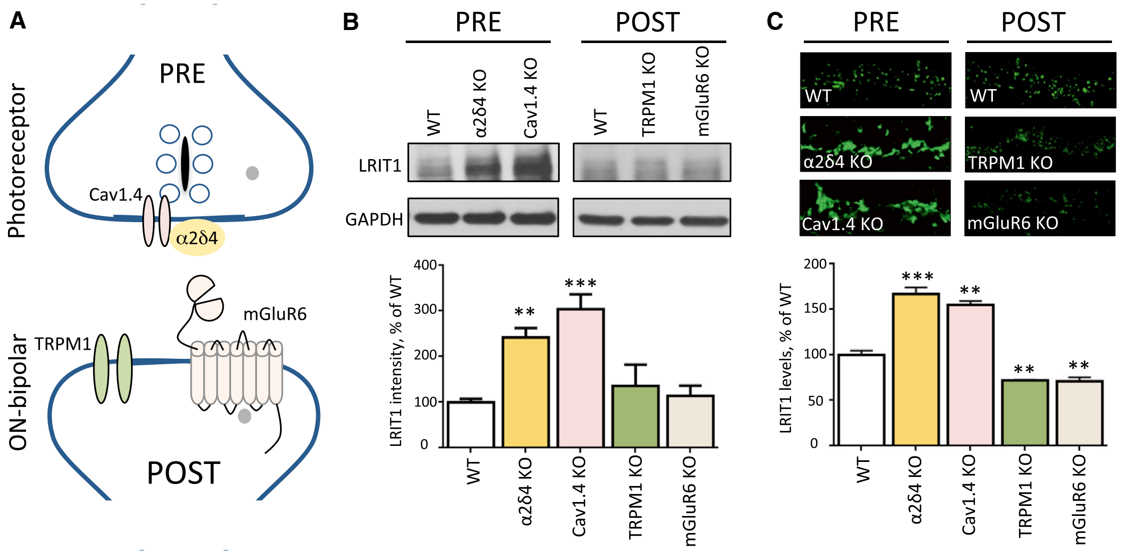

Figure 3. LRIT1 Synaptic Content Is Regulated by Changes in Presynaptic Release Apparatus

(A) Scheme of the molecular organization of the photoreceptor synapse. Knockout mice lacking pre- and post-synaptic players depicted on the scheme were analyzed in the experiments.

(B) Analysis of LRIT1 protein expression by western blotting in total lysates prepared from retinas of the respective mouse strains. Retinas from 3–5 mice for each genotype were used for the quantification of the LRIT1 band intensities and the values were normalized to WT. **$p < 0.01$, ***$p < 0.001$; t test.

(C) Analysis of LRIT1 synaptic targeting by immunohistochemical staining of retina cross-sections of knockout mouse retinas as indicated. OPL regions are shown. Scale bar, 10 μm. The intensity of LRIT1 signal in the OPL was quantified and normalized to WT values. Two sections from each retina, two retinas per genotype; **$p < 0.01$, ***$p < 0.001$; t test.

photoreceptors and ON-bipolar cells and is transported to the synapse where it is prominently present in the cone synaptic cleft.

Synaptic LRIT1 Content Is Modulated by Changes in Photoreceptor Activity

To assess the contribution of pre- and post-synaptic compartments to LRIT1 expression and localization, we examined several mouse models with deletions in key components of the photoreceptor pre-synaptic release machinery or the post-synaptic signaling complex in ON-BCs (Figure 3A). Analysis of LRIT1 expression in the retinas by western blotting revealed that the elimination of either the neurotransmitter receptor mGluR6, or the ON-BC effector channel TRPM1 has no effect on LRIT1 expression. In contrast, the knockout of $Ca_V1.4$ or α2δ4, which mediates the coupling of light-induced changes in membrane potential to glutamate release and synapse morphogenesis, respectively, results in a dramatic elevation of LRIT1 expression (Figure 3B). We further examined LRIT1 modulation at synapses by immunohistochemical staining of retinal cross-sections with anti-LRIT1 antibodies (Figure 3C). The results revealed a massive induction of LRIT1 content that occurs specifically at synapses where it is accumulated following deletion of pre-synaptic components: $Ca_V1.4$ and α2δ4. In contrast, the deletion of the postsynaptic mGluR6 or TRPM1 resulted only in a minor downregulation of LRIT1 in the OPL. These findings suggest that LRIT1 expression and synaptic accumulation is inversely dependent on the neurotransmitter release orchestrated by the $Ca_V1.4$ complex.

Elimination of LRIT1 Does Not Affect Structural or Molecular Architecture of Photoreceptor Synapses

To determine the role of LRIT1 in the retina, we obtained *Lrit1* knockout mice (*Lrit1*$^{-/-}$). In this line, the *Lrit1* allele is disrupted by placing a LacZ-Stop trap cassette immediately downstream of the first coding exon, thereby preventing translation of most of the *Lrit1* sequence (Figure 4A). Indeed, western blotting of whole retina lysates showed elimination of a specific band corresponding to LRIT1, indicating a complete ablation of LRIT1 protein (Figure 4B). Consistent with its transmembrane nature, the LRIT1 band was eliminated in knockout retinas and was concentrated in the membrane fraction while not present in the cytosol. In contrast, the major contaminating band was extracted in the soluble fraction, further confirming the specificity of assigning LRIT1 immunoreactivity (Figure S3). Immunohistochemical analysis showed elimination of immunostaining in the outer plexiform layer (OPL), additionally confirming the specificity of the antibodies (Figure 4C).

We found that deletion of LRIT1 did not affect overall morphology of the retina, its viability, or its laminar organization up to 3 months of age (Figures 4C and S1). Western blotting of *Lrit1*$^{-/-}$ retina lysates revealed no significant changes in the expression of key components of synaptic signaling between photoreceptors and ON-BCs. Furthermore, we found no changes in synaptic targeting of molecules involved in synaptic transmission (mGluR6, GPR179, $Ca_V1.4$, α2δ4) or the formation/maintenance of rod and cone synapses (ELFN1, LRIT3, connexin 36) (Figures 4E and S4). Detailed quantitative examination showed no changes in levels of mGluR6 in apposition to

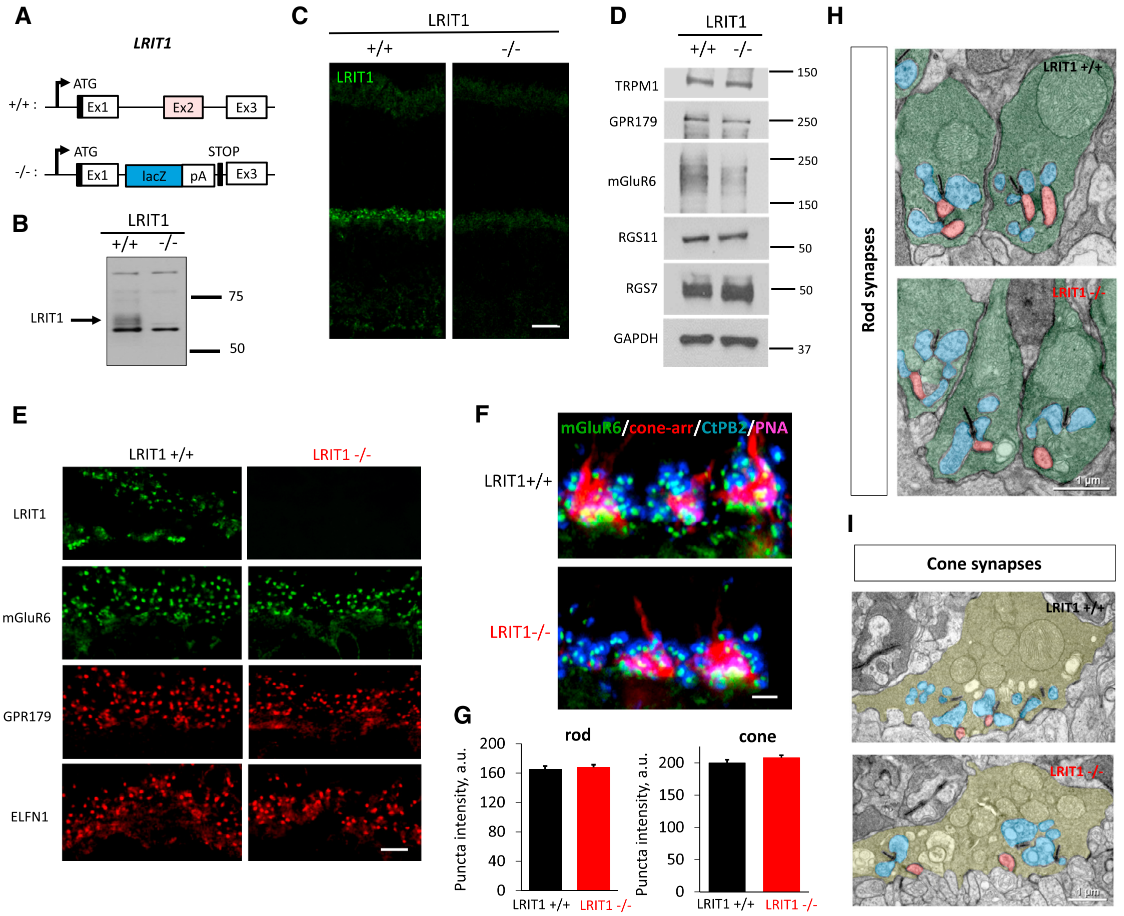

Figure 4. Generation and Characterization of Lrit1 Knockout Mice

(A) Scheme for targeting *Lrit1* gene. The deletion strategy included elimination of the critical coding exon 2 and introduction of the premature stop-codon preceding exon 3.

(B) Analysis of LRIT1 expression in wild-type and *Lrit1* knockout (–/–) mouse retinas by western blotting.

(C) Analysis of LRIT1 localization in wild-type and *Lrit1* knockout (–/–) mouse retinas by immunohistochemical staining of retina cross-sections, scale bar, 20 µm.

(D) Analysis of expression of proteins present in photoreceptor synapses by western blotting comparing wild-type and *Lrit1* knockout (–/–) mouse retinas.

(E) Analysis of distribution of proteins present in photoreceptor synapses by immunohistochemical staining of retina cross-sections of wild-type and *Lrit1* knockout (–/–) mouse retinas. OPL regions are shown, scale bar, 5 µm.

(F) Analysis of mGluR6 content in rod and cone synapses by immunohistochemistry. Staining with cone arrestin was used to define cone terminals and with PNA to identify active zones in the cone axons. Scale bar, 2.5 µm.

(G) Quantification of changes in mGluR6 staining in rod and cone synapses in the retinas of wild-type and *Lrit1* knockout (–/–) mice.

(H) Analysis of rod synapse morphology by electron microscopy. Rod terminals are labeled in green, horizontal cell processes in blue and ON-bipolar dendrites in red.

(I) Analysis of cone synapse morphology by electron microscopy. Cone terminals are labeled in pale green, horizontal cell processes in blue and bipolar dendrites in red.

active zones in rod or cone synaptic terminals (Figures 4F and 4G).

We further studied the fine synaptic morphology by transmission electron microscopy and found no obvious evidence for structural abnormalities. Rod spherules in *Lrit1*$^{-/-}$ retinas displayed normal shape and contained the expected elements, including the synaptic ribbon and the invaginating processes of horizontal cells and rod ON-BCs in direct apposition to synaptic ribbon (Figure 4H). Similarly, cone pedicles contained multiple ribbons and displayed clearly identifiable contacts with both horizontal cells and ON-BCs (Figure 4I). We thus conclude that deletion of LRIT1 had no major effect on the structural or molecular architecture of photoreceptor synapses.

Ablation of LRIT1 Causes Selective Deficits In Background Adaptation Of Cone Synaptic Signaling

We sought to determine a functional role for LRIT1 in light reception. Probing light-evoked responses of dark-adapted mice by electroretinography (ERG) revealed that *Lrit1*$^{-/-}$ mice displayed a normal ERG waveform; a-wave and b-wave components were indistinguishable from wild-type littermates under both scotopic (Figure 5A) and photopic (Figure 5B) light regimes that activate

OPEN
ACCESS
CellPress

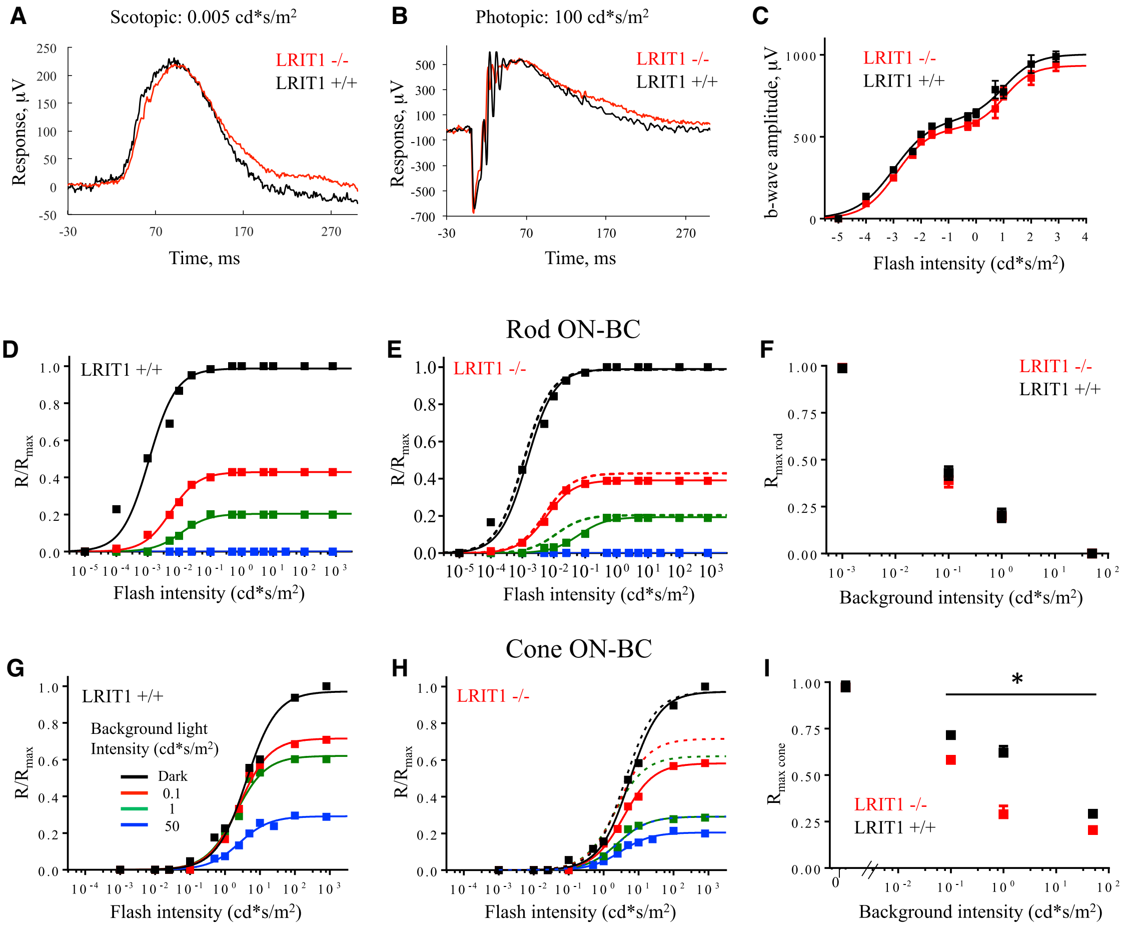

Figure 5. Analysis of LRIT1 Knockout by Electroretinography

(A) Representative electroretinography (ERG) waveform recorded from dark-adapted mice in response to scotopic flash of light.

(B) Representative ERG waveform recorded from dark-adapted mice in response to photopic flash of light.

(C) Light-dependence profile of b-wave amplitudes. 4–6 mice were used for each genotype.

(D) Rod-driven component of the b-wave across light intensities recorded in wild-type mice under dark-adapted conditions and various levels of background light. The first phase of the response at scotopic light intensities in (C) is shown.

(E) Rod-driven component of the b-wave across light intensities recorded in *Lrit1* knockout mice under dark-adapted conditions and various levels of background light. Dashed lines represent superimposed fits from WT in (D).

(F) Normalized changes in maximal amplitude of the rod-driven b-wave as a function of background light intensity recorded in both genotypes.

(G) Cone-driven component of the b-wave across light intensities recorded in wild-type mice under dark-adapted conditions and various levels of background light. The second phase of the response at photopic light intensities in (C) is shown.

(H) Cone-driven component of the b-wave across light intensities recorded in *Lrit1* knockout mice under dark-adapted conditions and various levels of background light. Dashed lines represent superimposed fits from wild-type mice in (G).

(I) Normalized changes in maximal amplitude of the cone-driven b-wave as a function of background light intensity recorded in both genotypes.

rods and cones, respectively. Quantitative analysis revealed no changes in the maximal ERG b-wave amplitude in either the rod or cone-driven components (Figure 5C), indicating no gross abnormality in synaptic transmission to ON-BCs in dark-adapted mice. Single-cell recordings from rod ON-BCs and cone photoreceptor corroborated this observation. Voltage clamp (V_m = −60 mV) recordings from *Lrit1*$^{-/-}$ and wild-type rod ON-BCs confirm the normal sensitivity of rod phototransduction and synaptic processing (Figure S2; Table S1). Furthermore, voltage clamp (V_m = −40 mV) recordings directly from cone photoreceptors revealed a robust maximum photocurrent with similar sensitivities and time courses in *Lrit1*$^{-/-}$ and *Lrit1*$^{+/+}$ retinas (Figure S2). We also observed no significant differences in the waveform or amplitudes of oscillatory potentials between the genotypes across the range of photopic light flashes, suggesting that LRIT1 ablation does not grossly affect processing of the visual signal by the inner retinal circuitry (Figure S5).

Next, we evaluated the role of LRIT1 in modulating the sensitivity of photoreceptor to ON-BC signaling during light adaptation. Consistent with previous reports, we found that increasing background light intensity reduced b-wave amplitudes elicited by both scotopic and photopic flashes. In the scotopic light intensity range, we observed no differences between genotypes over the range of background intensities (Figures 5D–5F),

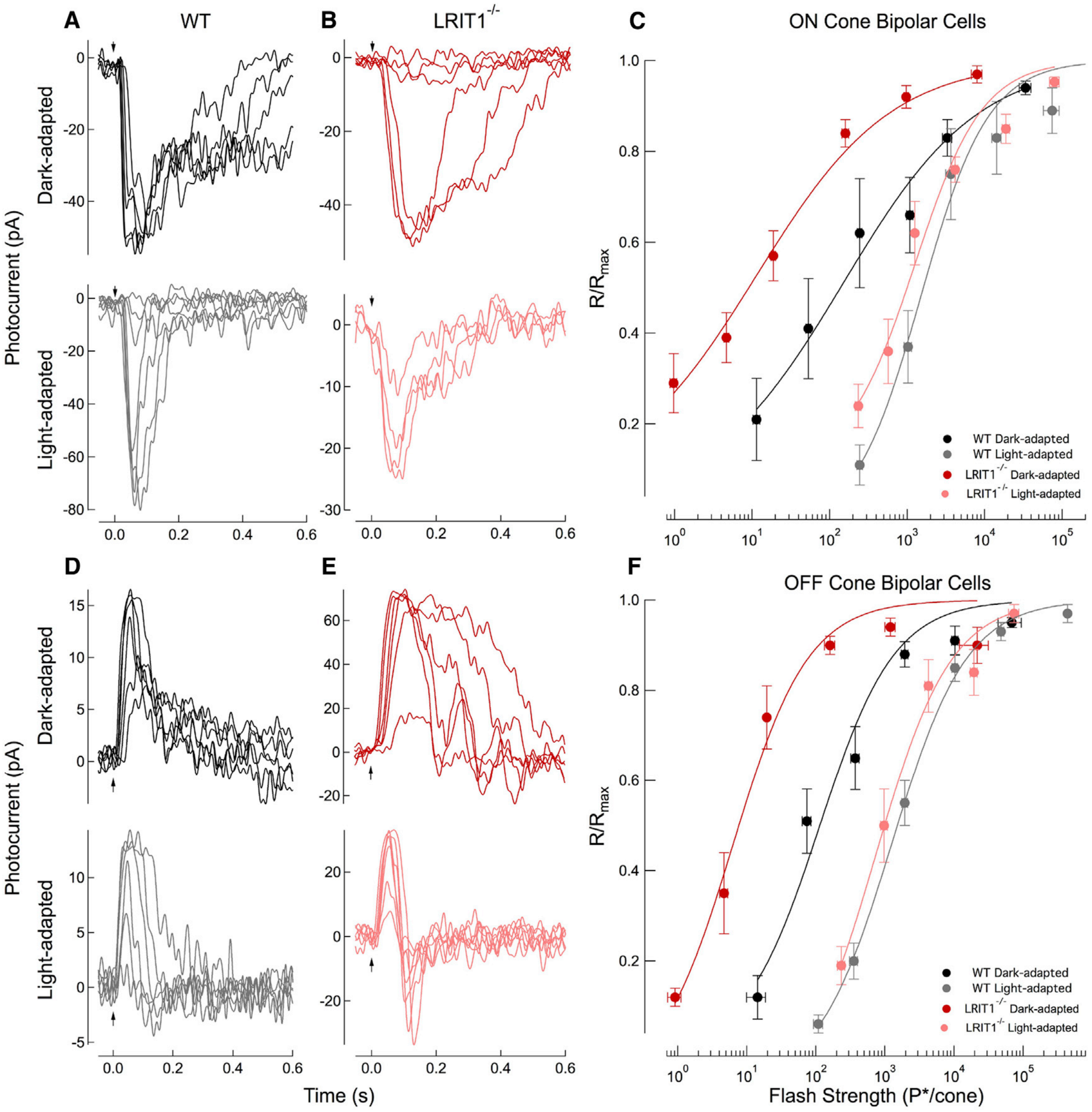

Figure 6. Analysis of Cone-to-BC Synaptic Transmission

(A) Whole-cell voltage clamp recordings ($V_m = -60$ mV) were made from wild-type ON-BCs in retinal slices. Flash families were collected in the dark-adapted state and following the presentation of a background light that generated 2,100 P*/cone/s, where P* is an estimated number of activated cone pigments. The dark-adapted flash family was collected during the presentation of 10-ms flashes delivering 38, 69, 160, 560, and 2,000 P*/cone, whereas the family collected during the presentation of background light delivered 160, 560, 2,000, 7,700, 33,000, 160,000, and 560,000 P*/cone. Top and bottom panels are recordings made in sequence from the same cell. In light adaptation experiments, retinas were exposed to light for 2 min before recording.

(B) Whole-cell voltage clamp recordings ($V_m = -60$ mV) from *Lrit1*$^{-/-}$ ON-BCs. Flash families again were collected in the dark-adapted state and following the presentation of a background light that generated 2,100 P*/cone/s. The dark-adapted flash family was collected for 10-ms flashes delivering 0.7, 2.5, 9.5, 38, 130, and 560 P*/flash, whereas families collected during the presentation of background light generated 270, 1,100, 2,000, and 3,000 P*/flash. Top and bottom panels are recordings made in sequence from the same cell.

(C) Response-intensity relationships for the dark-adapted and light-adapted WT and *Lrit1*$^{-/-}$ ON-BC flash families. Population data were averaged across flash strengths and fit with a Hill Curve with an exponent of 0.5 for the dark-adapted families and 1.0 for the light-adapted families. The half-maximal flash strength ($I_{1/2}$) for the fit was 150 P*/cone and 1,500 P*/cone for WT ON-BCs in the dark- and light-adapted states, respectively, a 10-fold shift. The half-maximal flash strength ($I_{1/2}$) for the fit was 11 P*/cone and 1,300 P*/cone for *Lrit1*$^{-/-}$ ON-BCs in the dark- and light-adapted states, respectively, a 120-fold shift.

(D) Whole-cell voltage clamp recordings ($V_m = -60$ mV) were made from WT OFF-BCs in retinal slices. Flash families were collected in the dark-adapted state and following the presentation of a background light that generated 2,100 P*/cone/s. The dark-adapted flash family was collected during the presentation of 10-ms flashes delivering 37, 69, 560, 2,000, 7,700, and 33,000 P*/cone, whereas the family collected during the presentation of background light delivered 160, 560, 2,000, 33,000, 160,000, and 560,000 P*/cone. Top and bottom panels are recordings made in sequence from the same cell.

(E) Whole-cell voltage clamp recordings ($V_m = -60$ mV) from *Lrit1*$^{-/-}$ OFF-BCs. Flash families again were collected in the dark-adapted state and following the presentation of a background light that generated 2,100 P*/cone/s. The dark-adapted flash family was collected for 10-ms flashes delivering 7.0, 24, 40, 78, 640,

(legend continued on next page)

thought to drive solely rod-mediated responses. In contrast, when a rod-suppressing background was delivered, the cone-mediated b-waves displayed reduced amplitudes in *Lrit1*$^{-/-}$ mice compared to their *Lrit1*$^{+/+}$ littermates (Figures 5G–5I). Together, these observations suggest that elimination of LRIT1 impairs light adaptation for cone-driven signals in ON-BCs, resulting in a more pronounced suppression of the ON-BC response amplitude by background light.

LRIT1 Controls the Sensitivity of Synaptic Transmission to Cone Bipolar Cells

To determine the mechanistic basis for the selectively impaired photopic ERG b-wave in background light, we measured the light-evoked responses of cone BCs in retinal slices. Recordings were made without a consideration of cone bipolar cell subtype, but appeared consistent across subtypes. Surprisingly, dark-adapted responses from *Lrit1*$^{-/-}$ cone ON-BCs were ~10-fold more sensitive than their wild-type (WT) counterparts (Figures 6A and 6B), as determined based on the flash strength that yields a half-maximal response. Interestingly, ~10-fold increased sensitivity was also observed in dark-adapted responses from *Lrit1*$^{-/-}$ cone OFF-BCs (Figures 6D and 6E), suggesting a common presynaptic origin of LRIT1 influence. Because the light intensities used to generate the flash families for *Lrit1*$^{-/-}$ BCs activate rods only, but rod ON-BC responses remain unchanged compared to WT cells (Figure S2; Table S1), these data suggest that the absence of LRIT1 influences specifically synaptic communication between cones and cone BCs.

We probed further the mechanism underlying observed reduction of ERG b-wave amplitude by background light that suggests reduced capacity of *Lrit1*$^{-/-}$ retinas for light adaptation. In the presence of a rod-suppressing background light delivering 2,100 P*/cone/s, flash families for cone ON- and OFF-BCs of both genotypes displayed a similar half-maximal flash strength (Figures 6A–6C; Table S1). Thus, the background light desensitized the *Lrit1*$^{-/-}$ cone BCs to a much greater extent than wild-type cone BCs, effectively diminishing their difference in sensitivity. It should be stressed that response families recorded in background light were from the same cone BCs that the dark-adapted data were collected from, permitting formal analysis of the changes in sensitivity induced by background. When maximum response amplitude in this background light was considered, we additionally found that *Lrit1*$^{-/-}$ ON-BCs displayed reduced amplitudes compared to their wild-type counterparts (Figures 6A and 6B; Table S1).

Thus, in addition to confirming deficits in light adaptation seen by ERG analysis, single-cell recordings further revealed an additional phenotype: increased dark-adapted sensitivity of ON an OFF cone BC light responses. This effect was not evident from the *en masse* analysis of neuronal responses to light by ERG likely due to large contributions by rod ON-BC activity, which effectively mask cone CB differences.

LRIT1 Is Needed for Achieving High Temporal Resolution of Visual Discrimination

To further assess the functional role of diminished light adaptation in *Lrit1*$^{-/-}$ retinas, we measured the capacity for the b-wave to track flickering light stimuli in the photopic light regime. Given the diminished cone-driven responses under background illumination, we tested processing of cone-derived signals in a flicker ERG paradigm that assesses mostly cone-mediated responses upon repeated stimulation, yet not fully eliminating rod contributions (Figure 7A). We found that under the conditions of this continuing light challenge, the *Lrit1* knockouts exhibited substantially reduced b-wave amplitudes consistent with deficits in background adaptation (Figures 7A and 7B). No genotype differences in the a-wave components of the flicker ERG were found, indicating normal rod and cone function (Figure 7C) and suggesting that the reduction in the b-wave is associated with changes in synaptic transmission to ON-BCs.

To understand the contribution of these observed adaptation deficits to vision, we evaluated behavior sensitivity in the optokinetic reflex (OKR) task on a steady light background (Figure 7D). Consistent with the reduction in the b-wave at the high-frequency stimulation, *Lrit1*$^{-/-}$ mice showed reduced visual acuity (i.e., spatial resolution of stimuli presented at high temporal frequency) (Figure 7E). However, we found no significant differences in the ability of *Lrit1*$^{-/-}$ mice to discriminate changes in contrast at any of the speed settings as compared to their wild-type littermates (Figure 7F). These observations suggest that loss of LRIT1 selectively compromises photopic visual acuity in a temporally challenging environment.

DISCUSSION

The diversity of the neuronal cell types is thought to underlie the unique properties of individual circuits that collectively specialize to perform a vast range of computations enabling complex behaviors (Lodato and Arlotta, 2015; Zeng and Sanes, 2017). Such design requires not only specificity in wiring between distinct cell classes, but also that emergent properties are matched for the demands of the circuit (Bargmann and Marder, 2013; de Wit and Ghosh, 2016). Here, we provide support for the functional specialization of synaptic contacts for cone photoreceptor, but not rods. We propose that cones selectively rely on an adhesion molecule, LRIT1, for controlling scaling of their synaptic output (Figure 7G). Specifically, we found that LRIT1 is recruited to the active zones of both rod and cone photoreceptors where it is found in complexes involving the postsynaptic neurotransmitter receptor, mGluR6, on ON-BC dendrites. The

and 2,300 P*/flash, whereas families collected during the presentation of background light generated 310, 640, 1,200, 2,300, 5,400, 12,000, and 55,000 P*/flash. Top and bottom panels are recordings made in sequence from the same cell.

(F) Response-intensity relationships for the dark-adapted and light-adapted WT and *Lrit1*$^{-/-}$ OFF-BC flash families. Population data were averaged across flash strengths and fit with a Hill Curve with an exponent of 0.8 for the dark-adapted and light-adapted families. The half-maximal flash strength ($I_{1/2}$) for the fit was 110 P*/cone and 1,300 P*/cone for WT OFF-BCs in the dark- and light-adapted states, respectively, a 12-fold shift. The half-maximal flash strength ($I_{1/2}$) for the fit was 6.3 P*/cone and 740 P*/cone for *Lrit1*$^{-/-}$ OFF-BCs in the dark- and light-adapted states, respectively, a 120-fold shift.

OPEN
ACCESS
CellPress

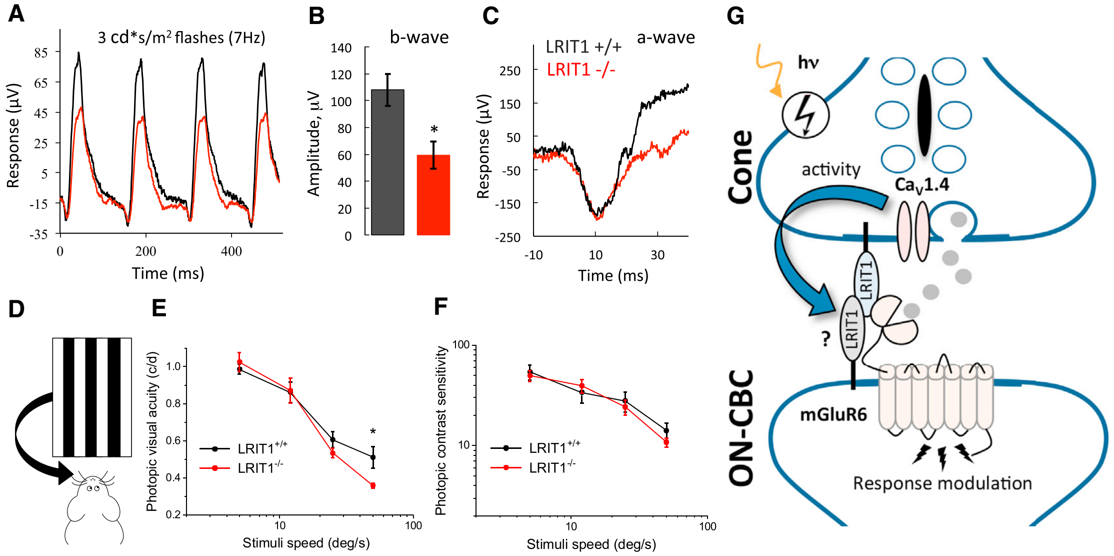

Figure 7. Visual Deficits in Mice Lacking LRIT1

(A) Representative flicker ERG traces in response to trains of stimulation delivered at 7 Hz frequency.

(B) Quantification of b-wave amplitude changes recorded in flicker ERG protocol. *$p < 0.05$, t test, 4–6 mice per genotype.

(C) Analysis of the a-wave recorded under flicker ERG protocol.

(D) Scheme of the optokinetic reflex testing principle to assess visual function in mice. The ability of mice to track virtually moving grids with varying contrast, spatial, and temporal properties is recorded.

(E) Dependence of photopic visual acuity on stimulation speed in the ORK test under 100% contrast of gratings. Deficits in photopic (light background of 1.1 cd s/m^2) contrast sensitivity of *Lrit1* knockout mice are apparent only at highest stimuli speed of 50°/s (error bars are SEM; t test: *$p < 0.05$, n = 5–6).

(F) Dependence of photopic contrast sensitivity on stimulation speed in the ORK test under constant light background of 1.1 cd/m^2 (error bars are SEM; t test: *$p < 0.05$, n = 5–6).

(G) Schematic representation of proposed role of LRIT1 in synaptic communication of cones. LRIT1 is expressed predominantly in cones and could also be present in cone BC with a possibility of forming *trans*-synaptic dimers given its capacity for heteromerization. It further interacts with postsynaptic mGluR6 receptor and presynaptic release apparatus containing Ca_V1.4 complex to adjust neurotransmitter signaling at the synapse in response to light adaptation scaling synaptic transmission of cones. Changes in photoreceptor synaptic activity modulate LRIT1 levels further contributing to adaptation.

deletion of LRIT1 did not affect the physical synapse assembly or molecular composition of synapses, yet it fundamentally changed their properties. Although LRIT1 is expressed in both rods and cones, we only detected effects at cone synapses. Remarkably, we found that knockout of LRIT1 increased the gain of synaptic transmission to ON and OFF bipolar cells, converting the sensitivity of cone ON-BCs to the range of rod ON-BCs. At the behavioral level, loss of LRIT1 compromised temporal aspects of photopic vision, although the exact mechanisms by which LRIT1 contributes to visual acuity remain to be established.

Despite the well documented role played by LRR proteins in synaptic structure (de Wit et al., 2011; Yogev and Shen, 2014), their influence on the functional aspects of signaling at metabotropic synapses have not been observed previously. We found that the augmentation of absolute sensitivity in cone BCs came at a price of reducing the ability of this synapse to adapt to continuous light exposure, thus limiting the operating range for transmitting signals. A key property of the cone system is the ability to operate over a wide range of background light, which they achieve by scaling their responsiveness with an increase in stimulation. Our findings demonstrate that LRIT1 is one of the molecular factors operating in cones that allow such adaptation at the level of controlling synaptic gain.

How can LRIT1 influence light sensitivity and synaptic scaling? Mechanistically, we think LRIT1 may exert its effects by several mechanisms, which are not necessarily mutually exclusive but remain to be established. We believe that the similar effects on the sensitivities of both ON and OFF CBs suggest that LRIT1 may act in photoreceptors to influence the presynaptic machinery that sets the rate of glutamate release. In line with this idea, we documented that the expression of LRIT1 is inversely dependent on the expression of the Ca_V1.4 channel complex. Interestingly, we found that neither targeting nor accumulation levels of Ca_V1.4 and $\alpha 2\delta 4$ was affected by LRIT1 elimination (Figure S4), suggesting that LRIT1 may influence glutamate release through fine-tuning the activity of the pre-synaptic calcium channel or other components of the release apparatus. Although we did not detect by EM any overt morphological changes precipitated by LRIT1 loss, some effects may also involve subtle structural changes in the synaptic cleft, including those related to positioning of active zone elements in relation to glutamate receptors that influence the efficiency of synaptic transmission.

Furthermore, physical interactions of LRIT1 with the principal neurotransmitter receptor mGluR6 may change the receptor signaling properties, perhaps by influencing the range of its responsiveness to glutamate. Such changes might affect the magnitude of the postsynaptic depolarization mediated by the

downstream TRPM1 channel. These mechanisms may be further integrated together via *trans*-synaptic LRIT1 homomerization, suggested by the ability of LRIT1 to form dimers (Gomi et al., 2000). Although our *in situ* hybridization data indicate that LRIT1 messenger RNA is indeed present in both photoreceptors and bipolar neurons, the postsynaptic accumulation of LRIT1 in the dendritic tips of bipolar neurons is less certain at the protein level. Further experimentation will be required to establish the relevance of this dimerization model. LRIT1 can also have an additional role in regulating rod-cone coupling. Although we showed no significant change in connexin 36 content induced by LRIT1 loss, it is still possible that LRIT1 regulates cone ON-BCs sensitivity by suppressing rod-to-cone coupling through changes in gap junction efficiency rather than the connexin36 expression. Ultimately, a combination of these mechanisms is likely shaping LRIT1's function and establishing their relative contributions will be an exciting future direction.

The discovery of LRIT1 as a key player in synaptic function of cones adds to a growing list of cell adhesion-like molecules that shape photoreceptor synapses. Interestingly, the expression of LRIT1 in photoreceptors was noted earlier (Gomi et al., 2000); however, it was reported to be localized to photoreceptor outer segments. We show clearly that LRIT1 is a synapse-specific protein located at both pre- and post-synaptic side of photoreceptor synapses using antibodies that we have validated against the *Lrit1*$^{-/-}$ retina. The closest homolog of LRIT1 is LRIT3, another photoreceptor synaptic protein expressed by ON-bipolar neurons (Zeitz et al., 2013). LRIT3 is indispensable for the synaptic transmission for photoreceptor signals, as its inactivation in mice and humans leads to complete disruption of both rod and cone synaptic signaling (Neuillé et al., 2014, 2015; Qian et al., 2015). Additionally, LRIT3 appears to play a role in the morphogenesis of cone synaptic contacts with ON-BCs (Neuillé et al., 2015). The relationship between LRIT1 and LRIT3 is unclear but based on their similarity in domain composition, ~40% sequence identity and propensity of LRIT1 to dimerize, it seems possible that both molecules may work together to orchestrate molecularly similar processes.

In addition, two other leucine-rich repeat proteins with similar organization are present at the photoreceptor synapses. Rods specifically express the cell adhesion molecule ELFN1, which like LRIT1 forms complexes with mGluR6 and plays an essential role in physical assembly of the rod to rod ON-BC connections (Cao et al., 2015). Both rods and cones also rely on nyctalopin (NYX), which does not appear to be involved in synapse assembly but rather in photoreceptor synaptic signaling. NYX is expressed in both rod and cone ON-BCs where it was shown to play a role for the synaptic localization of the effector channel, TRPM1 (Cao et al., 2011; Pearring et al., 2011). Thus, it appears that photoreceptor synapses utilize a host of LRR molecules, possibly interwoven together, to coordinate synaptic assembly with synaptic function. Intriguingly, the cell adhesion molecules in this organization are further integrated with the components of the GPCR signaling cascade, pointing to higher level scaffolding and integration of morphogenic factors with the synaptic transmission machinery. Deciphering the molecular logic of this synaptic code at photoreceptor synapses will inform more generally how metabotropic synapses are specified and regulated.

EXPERIMENTAL PROCEDURES

Detailed methods including reagents, mice strains, antibodies, procedures for western blotting, *in situ* hybridization, electroretinography (ERG), single-cell recordings, optokinetic testing (OKR) of vision in mice, immunoprecipitation, cell culture, appear in the Supplemental Experimental Procedures. When appropriate, statistical analyses were performed by employing Student's t test with sample size of more than 3 independent biological replicates (mice), and the data are reported together with SEM values.

Mice

Embryonic stem cell line with the *Lrit1*-targeted allele (Lrit1tm1a(EUCOMM) Hmgu) was obtained from EUCOMM (project 115689) and intended modifications described in the Results section were verified by sequencing and long range PCR. All studies involving mice were carried out in accordance with the NIH guidelines and were granted formal approval by the Institutional Animal Care and Use Committees.

Antibodies and Western Blotting

The generation of the most antibodies was described previously. Rabbit anti-LRIT1 antibodies were generated against mouse recombinant LRIT1 (aa 549–624). Rabbit anti-LRIT3 CT antibody was generated against human recombinant LRIT3 (aa 604–679).

Whole retinas were removed from mice and lysed by sonication in ice-cold PBS supplemented with 150 mM NaCl, 1% Triton X-100, and Complete protease inhibitor tablets (Roche). Following sonication, lysates were cleared by centrifugation, subjected to 12.5% SDS/PAGE. Protein bands were transferred onto PVDF membranes and probed with antibodies.

Cell Culture and Transfection

HEK293T cells were obtained from Clontech and cultured at 37°C and 5% CO_2 in DMEM supplemented with antibiotics, 10% FBS. HEK293T cells were transfected at ~70% confluency using Lipofectamine LTX (Invitrogen) according to the protocol of the manufacturer. The cells were harvested processed for co-immunoprecipitation.

Fluorescence *In Situ* Hybridization

The mRNA expression was evaluated with ViewRNATM 2-plex *In Situ* Hybridization Assay (Panomics, Santa Clara, CA) using the following probes: *Lrit1* (NM_146245.2; Cat# VB1-17470). 12-μm sections were post-fixed in 4% paraformaldehyde for 10 min, washed, and incubated with the probes.

Immunohistochemistry

Dissected eyecups were fixed for 15 min in 4% paraformaldehyde, cryoprotected with 30% sucrose in PBS for 2 hr at room temperature, and embedded in optimal cutting temperature medium. 12-μm frozen sections were obtained and blocked in PT1 (PBS with 0.1% Triton X-100 and 10% donkey serum) for 1 hr then incubated with primary antibody in PT2 (PBS with 0.1% Triton X-100 and 2% donkey serum) for at least 1 hr. After four washes with PBS with 0.1% Triton, sections were incubated with fluorophore-conjugated secondary antibodies in PT2 for 1 hr. After four washes, sections were mounted in Fluoromount (Sigma).

Electroretinography

Electroretinograms were recorded by using the UTA system and a Big-Shot Ganzfeld (LKC Technologies). Mice (~4–8 weeks old) were dark-adapted (≥6 hr) and prepared for recordings using a red dim light. ERG signals were sampled at 1 kHz and recorded with 0.3-Hz low-frequency and 300-Hz high-frequency cut-offs.

Single-Cell Recordings

Light-evoked responses from photoreceptors and bipolar cells were recorded retinal slices using methods described previously (Okawa et al., 2010). Briefly,

mice were dark-adapted overnight and euthanized according to protocols approved by the University of California, Los Angeles Animal Research Committee (Protocol 14-005-11). Slices were superfused with bicarbonate-buffered Ames media (equilibrated with 5% CO_2/95% O_2) heated to 35°C–37°C, visualized under infrared illumination, and stimulated with a blue light-emitting diode (λ_{max} ~405 nm). Light-evoked responses were measured using patch electrodes in voltage-clamp mode.

Evaluation of Mouse Vision by Optokinetic Reflex Test

Photopic contrast sensitivity of mice was evaluated from optomotor responses using a two-alternative forced-choice protocol, as previously described (Kolesnikov et al., 2011; Umino et al., 2008).

SUPPLEMENTAL INFORMATION

Supplemental Information includes Supplemental Experimental Procedures, five figures, and one table and can be found with this article online at https://doi.org/10.1016/j.celrep.2018.03.008.

ACKNOWLEDGMENTS

We thank Ms. Natalia Martemyanova for performing genetic crosses needed to obtain mice utilized in these studies. This work was supported by NIH grants EY018139, MH105482, DA026405 (to K.A.M.), EY028033 (to K.A.M. and A.P.S.), EY026675 (to V.J.K.), and EY019312 (to V.J.K.) and unrestricted grants from Research to Prevent Blindness to the Departments of Ophthalmology at UCLA and Washington University.

AUTHOR CONTRIBUTIONS

I.S., Y.C., and Y.W. conducted most of the cell biological and biochemical experiments. I.S. and Y.W. performed ERG and analyzed the data. C.O. performed cell biological experiments and analyzed the data. N.T.I. and J.P. performed single-cell recordings. N.K. performed EM experiments and analyzed the data. A.V.K. and V.J.K. performed OKR evaluation of mouse vision and analyzed the data. A.P.S. designed electrophysiological experiments and analyzed the data. K.A.M. designed the study, analyzed the data, and wrote the paper. All authors contributed to writing and editing the manuscript.

DECLARATION OF INTERESTS

The authors declare no competing interests.

Received: December 20, 2017
Revised: February 10, 2018
Accepted: February 28, 2018
Published: March 27, 2018

REFERENCES

Bargmann, C.I., and Marder, E. (2013). From the connectome to brain function. Nat. Methods *10*, 483–490.

Burkhardt, D.A. (1994). Light adaptation and photopigment bleaching in cone photoreceptors in situ in the retina of the turtle. J. Neurosci. *14*, 1091–1105.

Cao, Y., Posokhova, E., and Martemyanov, K.A. (2011). TRPM1 forms complexes with nyctalopin in vivo and accumulates in postsynaptic compartment of ON-bipolar neurons in mGluR6-dependent manner. J. Neurosci. *31*, 11521–11526.

Cao, Y., Sarria, I., Fehlhaber, K.E., Kamasawa, N., Orlandi, C., James, K.N., Hazen, J.L., Gardner, M.R., Farzan, M., Lee, A., et al. (2015). Mechanism for Selective Synaptic Wiring of Rod Photoreceptors into the Retinal Circuitry and Its Role in Vision. Neuron *87*, 1248–1260.

de Wit, J., and Ghosh, A. (2016). Specification of synaptic connectivity by cell surface interactions. Nat. Rev. Neurosci. *17*, 22–35.

de Wit, J., Hong, W., Luo, L., and Ghosh, A. (2011). Role of leucine-rich repeat proteins in the development and function of neural circuits. Annu. Rev. Cell Dev. Biol. *27*, 697–729.

Euler, T., Haverkamp, S., Schubert, T., and Baden, T. (2014). Retinal bipolar cells: elementary building blocks of vision. Nat. Rev. Neurosci. *15*, 507–519.

Gomi, F., Imaizumi, K., Yoneda, T., Taniguchi, M., Mori, Y., Miyoshi, K., Hitomi, J., Fujikado, T., Tano, Y., and Tohyama, M. (2000). Molecular cloning of a novel membrane glycoprotein, pal, specifically expressed in photoreceptor cells of the retina and containing leucine-rich repeat. J. Neurosci. *20*, 3206–3213.

Govardovskii, V.I., Calvert, P.D., and Arshavsky, V.Y. (2000). Photoreceptor light adaptation. Untangling desensitization and sensitization. J. Gen. Physiol. *116*, 791–794.

Gregg, R.G., Ray, T.A., Hasan, N., McCall, M.A., and Peachey, N.S. (2014). Interdependence among members of the mGluR6 G-protein mediated signalplex of retinal depolarizing bipolar cells. In G Protein Signaling Mechanisms in the Retina, A.P. Sampath and K. Martemyanov, eds. (Springer), pp. 67–79.

Heidelberger, R., Thoreson, W.B., and Witkovsky, P. (2005). Synaptic transmission at retinal ribbon synapses. Prog. Retin. Eye Res. *24*, 682–720.

Hoon, M., Okawa, H., Della Santina, L., and Wong, R.O. (2014). Functional architecture of the retina: development and disease. Prog. Retin. Eye Res. *42*, 44–84.

Ingram, N.T., Sampath, A.P., and Fain, G.L. (2016). Why are rods more sensitive than cones? J. Physiol. *594*, 5415–5426.

Joiner, M.L., and Lee, A. (2015). Voltage-gated Cav1 channels in disorders of vision and hearing. Curr. Mol. Pharmacol. *8*, 143–148.

Kolesnikov, A.V., Rikimaru, L., Hennig, A.K., Lukasiewicz, P.D., Fliesler, S.J., Govardovskii, V.I., Kefalov, V.J., and Kisselev, O.G. (2011). G-protein betagamma-complex is crucial for efficient signal amplification in vision. J. Neurosci. *31*, 8067–8077.

Korenbrot, J.I. (2012). Speed, sensitivity, and stability of the light response in rod and cone photoreceptors: facts and models. Prog. Retin. Eye Res. *31*, 442–466.

Kramer, R.H., and Davenport, C.M. (2015). Lateral inhibition in the vertebrate retina: the case of the missing neurotransmitter. PLoS Biol. *13*, e1002322.

Lodato, S., and Arlotta, P. (2015). Generating neuronal diversity in the mammalian cerebral cortex. Annu. Rev. Cell Dev. Biol. *31*, 699–720.

Martemyanov, K.A., and Sampath, A.P. (2017). The transduction cascade in retinal ON-bipolar cells: signal processing and disease. Annu. Rev. Vis. Sci. *3*, 25–51.

Matthews, H.R., Fain, G.L., Murphy, R.L.W., and Lamb, T.D. (1990). Light adaptation in cone photoreceptors of the salamander: a role for cytoplasmic calcium. J. Physiol. *420*, 447–469.

Morgans, C.W., Brown, R.L., and Duvoisin, R.M. (2010). TRPM1: the endpoint of the mGluR6 signal transduction cascade in retinal ON-bipolar cells. BioEssays *32*, 609–614.

Morshedian, A., and Fain, G.L. (2017). Light adaptation and the evolution of vertebrate photoreceptors. J. Physiol. *595*, 4947–4960.

Neuillé, M., El Shamieh, S., Orhan, E., Michiels, C., Antonio, A., Lancelot, M.E., Condroyer, C., Bujakowska, K., Poch, O., Sahel, J.A., et al. (2014). Lrit3 deficient mouse (nob6): a novel model of complete congenital stationary night blindness (cCSNB). PLoS ONE *9*, e90342.

Neuillé, M., Morgans, C.W., Cao, Y., Orhan, E., Michiels, C., Sahel, J.A., Audo, I., Duvoisin, R.M., Martemyanov, K.A., and Zeitz, C. (2015). LRIT3 is essential to localize TRPM1 to the dendritic tips of depolarizing bipolar cells and may play a role in cone synapse formation. Eur. J. Neurosci. *42*, 1966–1975.

Nikonov, S.S., Kholodenko, R., Lem, J., and Pugh, E.N., Jr. (2006). Physiological features of the S- and M-cone photoreceptors of wild-type mice from single-cell recordings. J. Gen. Physiol. *127*, 359–374.

Okawa, H., Miyagishima, K.J., Arman, A.C., Hurley, J.B., Field, G.D., and Sampath, A.P. (2010). Optimal processing of photoreceptor signals is required to maximize behavioural sensitivity. J. Physiol. *588*, 1947–1960.

Pearring, J.N., Bojang, P., Jr., Shen, Y., Koike, C., Furukawa, T., Nawy, S., and Gregg, R.G. (2011). A role for nyctalopin, a small leucine-rich repeat protein, in localizing the TRP melastatin 1 channel to retinal depolarizing bipolar cell dendrites. J. Neurosci. *31*, 10060–10066.

Pugh, E.N., Jr., Nikonov, S., and Lamb, T.D. (1999). Molecular mechanisms of vertebrate photoreceptor light adaptation. Curr. Opin. Neurobiol. *9*, 410–418.

Pugh, E.N., Jr., and Lamb, T.D. (2000). Phototransduction in Vertebrate Rods and Cones: Molecular Mechanisms of Amplification, Recovery and Light Adaptation (Amsterdam: Elsevier).

Qian, H., Ji, R., Gregg, R.G., and Peachey, N.S. (2015). Identification of a new mutant allele, Grm6(nob7), for complete congenital stationary night blindness. Vis. Neurosci. *32*, E004.

Soo, F.S., Detwiler, P.B., and Rieke, F. (2008). Light adaptation in salamander L-cone photoreceptors. J. Neurosci. *28*, 1331–1342.

Stockman, A., Langendörfer, M., Smithson, H.E., and Sharpe, L.T. (2006). Human cone light adaptation: from behavioral measurements to molecular mechanisms. J. Vis. *6*, 1194–1213.

Thoreson, W.B., Rabl, K., Townes-Anderson, E., and Heidelberger, R. (2004). A highly Ca2+-sensitive pool of vesicles contributes to linearity at the rod photoreceptor ribbon synapse. Neuron *42*, 595–605.

Tummala, S.R., Dhingra, A., Fina, M.E., Li, J.J., Ramakrishnan, H., and Vardi, N. (2016). Lack of mGluR6-related cascade elements leads to retrograde trans-synaptic effects on rod photoreceptor synapses via matrix-associated proteins. Eur. J. Neurosci. *43*, 1509–1522.

Umino, Y., Solessio, E., and Barlow, R.B. (2008). Speed, spatial, and temporal tuning of rod and cone vision in mouse. J. Neurosci. *28*, 189–198.

Vardi, N., Dhingra, A., Zhang, L., Lyubarsky, A., Wang, T.L., and Morigiwa, K. (2002). Neurochemical organization of the first visual synapse. Keio J. Med. *51*, 154–164.

Vroman, R., Klaassen, L.J., and Kamermans, M. (2013). Ephaptic communication in the vertebrate retina. Front. Hum. Neurosci. *7*, 612.

Wang, Y., Fehlhaber, K.E., Sarria, I., Cao, Y., Ingram, N.T., Guerrero-Given, D., Throesch, B., Baldwin, K., Kamasawa, N., Ohtsuka, T., et al. (2017). The auxiliary calcium channel subunit alpha2delta4 is required for axonal elaboration, synaptic transmission, and wiring of rod photoreceptors. Neuron *93*, 1359–1374.

Wu, S.M. (1994). Synaptic transmission in the outer retina. Annu. Rev. Physiol. *56*, 141–168.

Yang, X.L., and Wu, S.M. (1997). Response sensitivity and voltage gain of the rod- and cone-bipolar cell synapses in dark-adapted tiger salamander retina. J. Neurophysiol. *78*, 2662–2673.

Yau, K.W., and Hardie, R.C. (2009). Phototransduction motifs and variations. Cell *139*, 246–264.

Yogev, S., and Shen, K. (2014). Cellular and molecular mechanisms of synaptic specificity. Annu. Rev. Cell Dev. Biol. *30*, 417–437.

Zeitz, C., Jacobson, S.G., Hamel, C.P., Bujakowska, K., Neuillé, M., Orhan, E., Zanlonghi, X., Lancelot, M.E., Michiels, C., Schwartz, S.B., et al.; Congenital Stationary Night Blindness Consortium (2013). Whole-exome sequencing identifies LRIT3 mutations as a cause of autosomal-recessive complete congenital stationary night blindness. Am. J. Hum. Genet. *92*, 67–75.

Zeitz, C., Robson, A.G., and Audo, I. (2015). Congenital stationary night blindness: an analysis and update of genotype-phenotype correlations and pathogenic mechanisms. Prog. Retin. Eye Res. *45*, 58–110.

Zeng, H., and Sanes, J.R. (2017). Neuronal cell-type classification: challenges, opportunities and the path forward. Nat. Rev. Neurosci. *18*, 530–546.

Cell Reports, Volume *22*

Supplemental Information

LRIT1 Modulates Adaptive Changes in Synaptic Communication of Cone Photoreceptors

Ignacio Sarria, Yan Cao, Yuchen Wang, Norianne T. Ingram, Cesare Orlandi, Naomi Kamasawa, Alexander V. Kolesnikov, Johan Pahlberg, Vladimir J. Kefalov, Alapakkam P. Sampath, and Kirill A. Martemyanov

SUPPLEMENTAL INFORMATION

Supplemental Figures and Tables

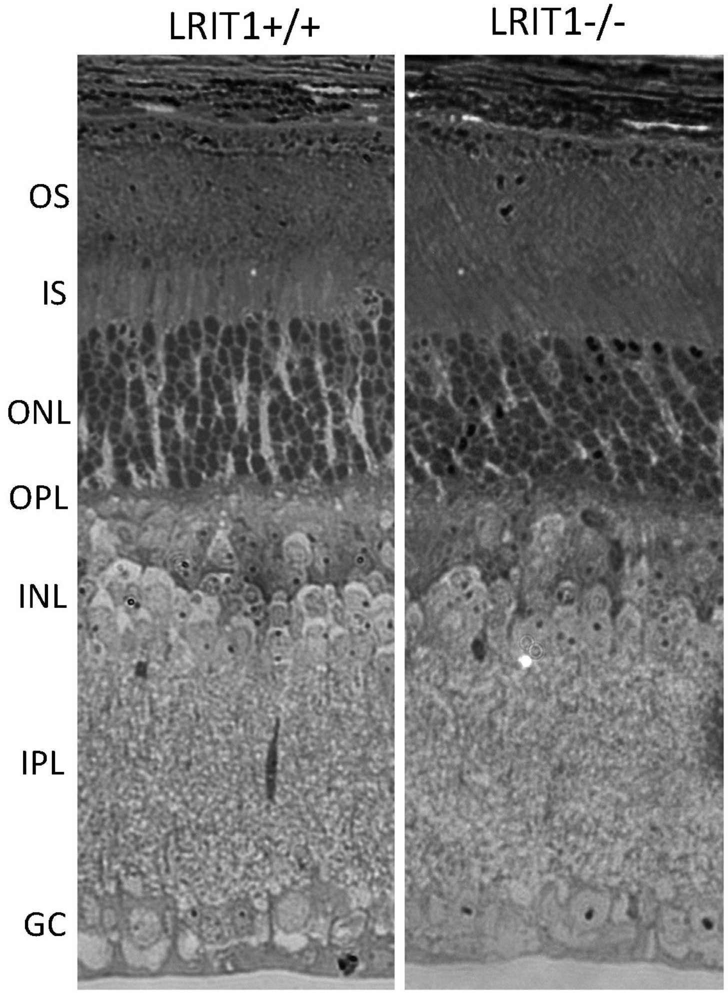

Figure S1. Related to Figure 4. Normal retina morphology in *Lrit1*$^{-/-}$ mice.
Analysis of the retina morphology by toluidine blue staining of ultra-thin (0.2 μm) retina cross-sections

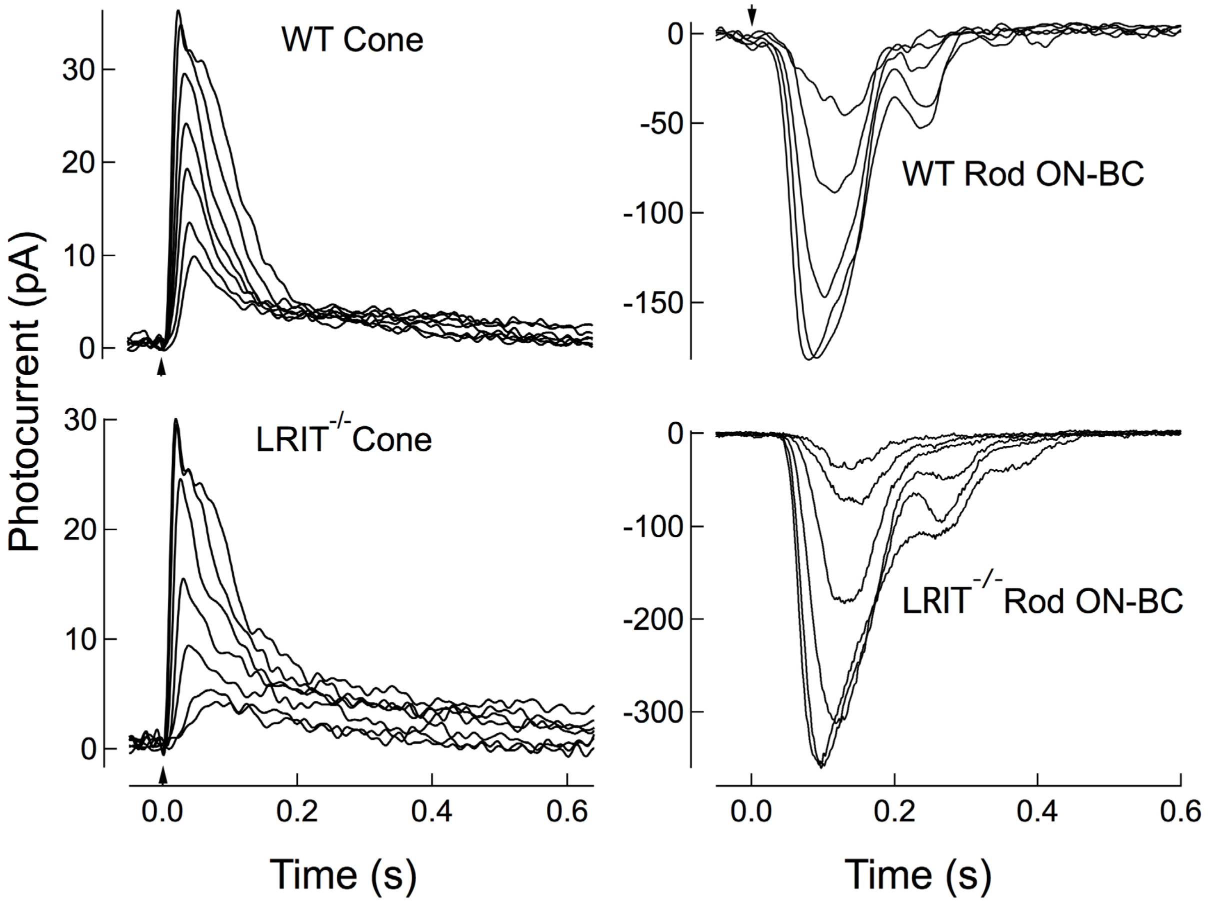

Figure S2. Related to Figure 6. Normal cone photoreceptor and rod ON-BC responses in *Lrit1*$^{-/-}$ mice.

Voltage-clamp (V_m = -40 mV) recordings from WT and *Lrit1*$^{-/-}$ cones. WT responses were evoked by 10ms flashes generating 350, 720, 1,400, 2,600, 4,500, 14,000, and 31,000 P*. *Lrit1*$^{-/-}$ responses were evoked by 10 ms flashes generating 70, 160, 560, 2,000, 7,700, 33,000, and 76,000 P*. Voltage-clamp (V_m = -60 mV) recordings from WT and *Lrit1*$^{-/-}$ rod ON-BCs. WT response were evoked by 10 ms flashes generating 1.5, 2.8, 6.6, 11, and 23 activated rhodopsins (R*). *Lrit1*$^{-/-}$ response were evoked by 10 ms flashes generating 1.2, 3.5, 5.9, 8.3, 13, and 20 R*.

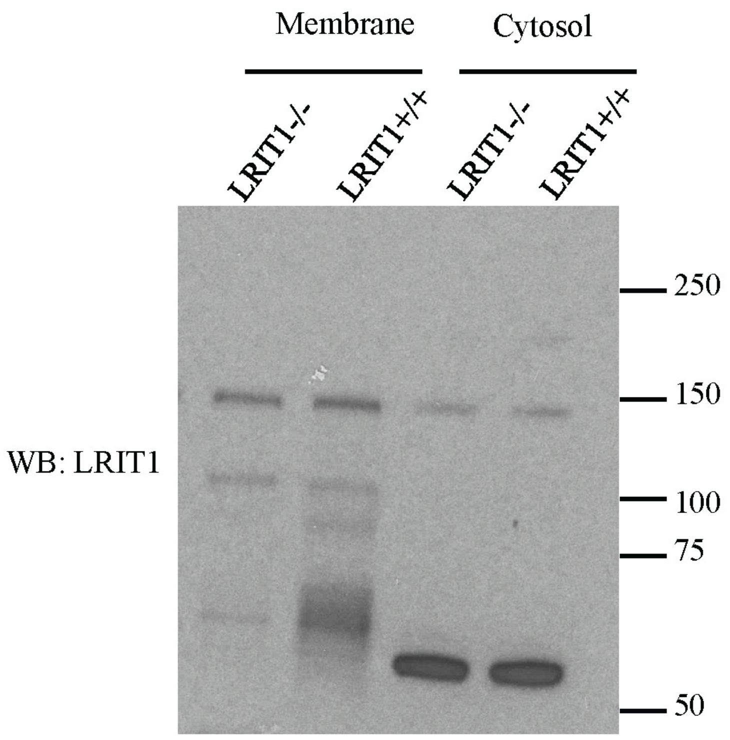

Figure S3. Related to Figure 4. Specific immunoreactive band for LRIT1 is associated with membranes

Western blotting analysis of LRIT1 using whole retina lysate from both wild-type (*Lrit1*$^{+/+}$) and *Lrit1*$^{-/-}$ mice after membrane fractionation. Equal amount of total protein from both cytosolic and membrane portion of each genotype was loaded and analyzed by western blot using specific LRIT1 antibody. Note that the intense band around 60kD detected by this antibody showed in cytosolic portion but not in membrane portion in both genotypes confirming its non-specificity.

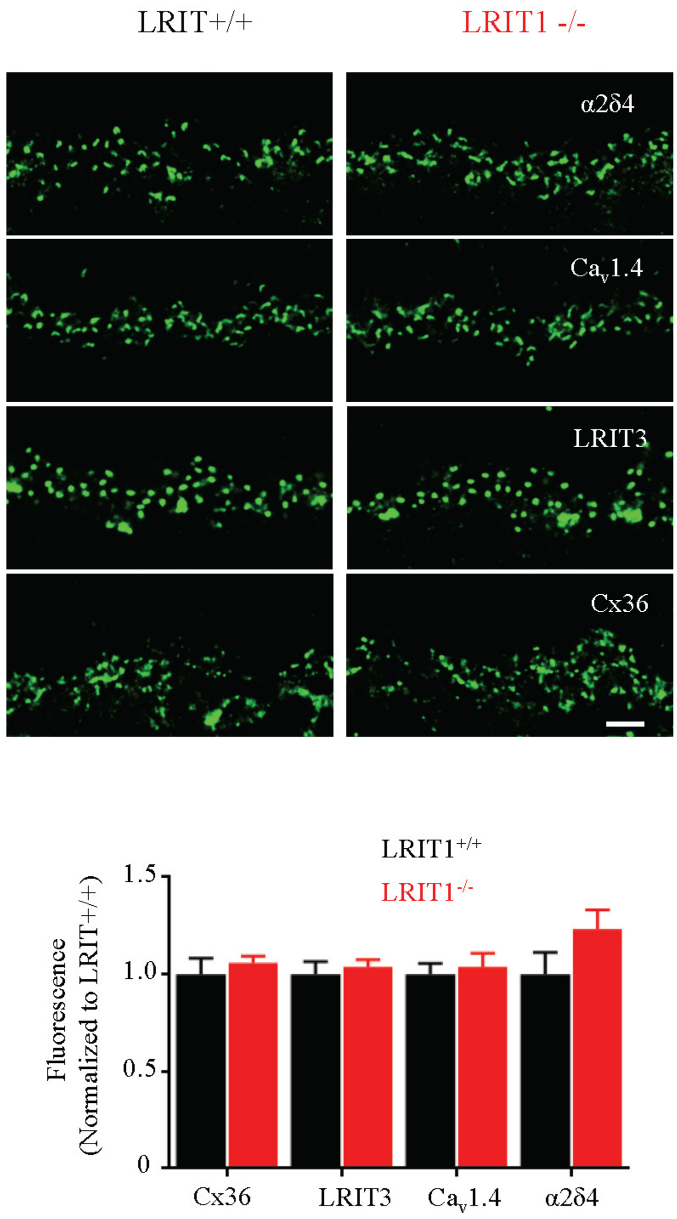

Figure S4. Related to Figure 4. The effect of *Lrit1* knockout on content of key synaptic proteins.
A, Representative confocal pictures of retina cross-sections from wild type (*Lrit1*$^{+/+}$) and LRIT1 knockout (*Lrit1*$^{-/-}$) mice stained with specific antibodies against different synaptic molecules as indicated. Scale bar: 20µm). OPL regions are shown. **B,** Quantification of the immunofluorescence intensities of synaptic molecules examined in panel A. Mean values were normalized to *Lrit1*$^{+/+}$ controls and plotted with corresponding SEMs.

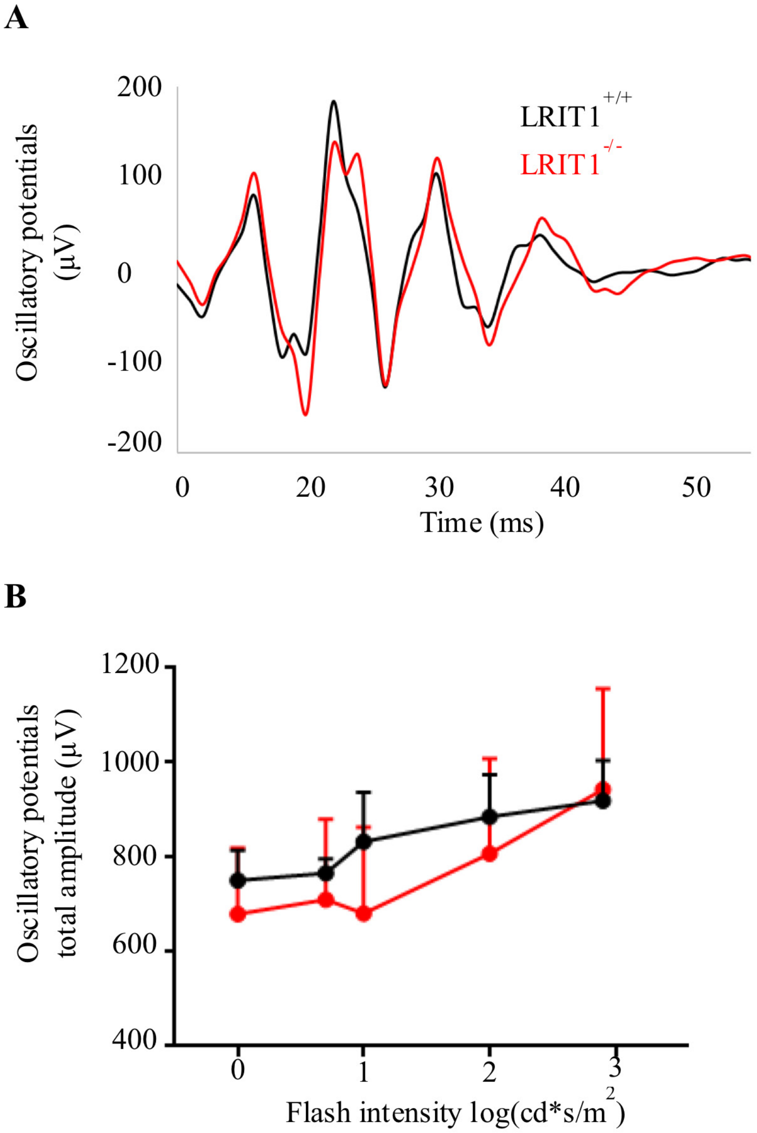

Figure S5. Related to Figure 5. Analysis of the oscillatory potentials of ERG recording.
A, Representative ERG traces of oscillatory potential components measured from dark-adapted mice stimulated with 100 cd/m^2 light. **B,** Statistical analysis of the oscillatory potential amplitudes at different intensities of light stimuli in *Lrit1*$^{+/+}$ and *Lrit1*$^{-/-}$ mice (Mean values and SEM were shown, multiple t-test, N=3 for *Lrit1*$^{+/+}$ and N=5 for *Lrit1*$^{-/-}$).

Table S1: Related to Figure 6. Response Characteristics of Photoreceptors and Bipolar Cells.

**Cell Type**		**V_m (mV)**	**$I_{1/2}$ (P*/cone)**	**R_{max} (pA)**
Cone Photoreceptor	WT	-37 ± 2.1 (13)	1800 ± 270 (20)	22 ± 1.9 (20)
	Lrit1$^{-/-}$	-33 ± 1.5 (7)	2600 ± 340 (8)	29 ± 1.4 (8)
Rod ON-BC** Dark-adapted	WT	-	4.8 ± 2.7 (2)	220 ± 41 (2)
	Lrit1$^{-/-}$	-	5.0 + 0.3 (5)	220 ± 51 (5)
Cone ON-BC Dark-adapted	WT	-	480 ± 92 (7)	70 ± 7.3 (7)
	Lrit1$^{-/-}$	-	28 ± 2.0 (22)	31 ± 1.0 (22)
Cone ON-BC 210 P*/cone/sec	WT	-	4700 ± 1600 (5)	48 ± 4.5 (5)*
	Lrit1$^{-/-}$	-	5000 ± 530 (14)	14 ± 0.88 (14)*
Cone OFF-BC Dark-adapted	WT	-	220 ± 110 (7)	34 ± 11 (7)
	Lrit1$^{-/-}$	-	9.5 ± 3.0 (7)	45 ± 10 (7)
Cone OFF-BC 210 P*/cone/sec	WT	-	1100 ± 320 (7)	26 ± 5.7 (7)
	Lrit1$^{-/-}$	-	1500 ± 560 (7)	27 ± 6 (7)

Data from (n) individual cells were first fit, then averaged. Response characteristics are documented as mean ± SEM (n)

* Denotes $p<0.05$ based on a paired student's t-test

** Note that rod ON-BC values for sensitivity are calculated in R*/rod

Supplemental Experimental Procedures

Mice

ES cell line with the *Lrit1* targeted allele (Lrit1tm1a(EUCOMM)Hmgu was obtained from EUCOMM (project 115689) and intended modifications described in the Results section were verified by sequencing and long range PCR. The ES cell line was used to generate chimeric mice by blastocyst injection at the Mouse Genetics core at the Scripps Research Institute. Resulting progeny was crossed with C57BL6 strain to establish germline transmission and the F1 mice were further crossed with the germline Cre-expressor strain B6.FVB-TgN(EIIa-Cre)C5379Lmgd (Jackson) to achieve elimination of exon 2 by LoxP recombination. The resulting constitutive *Lrit1* heterozygous knockout mice were inbred to produce -/- and +/+ littermates used in the study. Mice of both sexes were used in the experiments during daytime. Mice used in the study were 1–3 months old, and were maintained on a diurnal 12 h light/dark cycle. Procedures involving mice strictly followed NIH guidelines and were approved by the Institutional Animal Care and Use Committees at Scripps Florida, Washington University and the University of California, Los Angeles.

DNA constructs

Full-length cDNAs encoding human mGluR6 was purchased from Missouri S&T cDNA Resource Center (Cat# GRM6000000). Full-length cDNA encoding mouse *Lrit1* was purchased from Open Biosystems (Clone ID: 5401567). The C-terminal c-myc tagged mouse full length *Lrit1*, NT-LRIT1 (aa 1-527) were amplified from mouse *Lrit1* cDNA clone then sub-cloned into a pcDNA3.1/V5-His-TOPO (Invitrogen) expression vector according to manufacturer's specifications.

Antibodies and Western Blotting

The generation of the following antibodies was described previously: sheep anti-RGS11 (Cao et al., 2008), sheep anti-TRPM1 (Cao et al., 2011). Rabbit anti-RGS7 (7RC1), was a generous gift from William Simonds (NINDDK/NIH), and the guinea pig anti-mGluR6 antibody was a gift from Dr. Takahisa Furukawa (Osaka University). Rabbit anti-Cav1.4 antibody was a generous gift from Dr. Amy Lee (University of Iowa). Rabbit anti-LRIT1 antibodies were generated against mouse recombinant LRIT1 (aa 549-624). Rabbit anti-LRIT3 CT antibody was generated against human recombinant LRIT3 (aa 604-679). Rabbit anti-ELFN1 (NTR) and rabbit anti-ELFN1 (CTR) antibodies were generated against synthetic peptides of mouse ELFN1 (aa 305-320 and aa 530-547, respectively). Mouse anti-PKCα (ab11723; Abcam), mouse anti-CtBP2 (612044; BD Biosciences), mouse anti-Connexin36 (Clone 8F6.2, Millipore Bioscience Research Reagents, MAB3045), rabbit anti-α2δ4 (Aviva, OAAF04451) and mouse anti-GAPDH (Millipore; MAB374) were purchased.

Whole retinas were removed from mice and lysed by sonication in ice-cold PBS supplemented with 150 mM NaCl, 1% Triton X-100, and Complete protease inhibitor tablets (Roche). Lysates were cleared by centrifugation at 20,800 × *g* for 15 min at 4 °C. Total protein concentration in the supernatant was measured by using BCA Protein Assay Kit (Pierce). Supernatants were added with SDS sample buffer (pH 6.8) containing 8 M urea and were subjected to 12.5% SDS/PAGE. Protein bands were transferred onto PVDF membranes, subjected to Western blot analysis by using HRP-conjugated secondary antibodies, and detected by using ECL West Pico system (Pierce). Signals were captured on film and scanned by densitometer. For quantification, band intensities were determined by using NIH ImageJ software. Integrated intensity of GAPDH was used for data normalization.

Membrane fractionation was done as described in “Preparative immunoprecipitation of mGluR6 complexes” section.

Preparative immunoprecipitation of mGluR6 complexes from mouse retina and mass-spectrometry

Retinas were removed from mice and lysed by sonication in ice-cold PBS supplemented with 150 mM NaCl and Complete protease inhibitor tablets (Roche). After 30-minute centrifugation at 100,000 x g, 4 °C, the pellet was resuspended in ice-cold PBS IP buffer supplemented with 150 mM NaCl, 1% Triton X-100, and Complete protease inhibitor tablets (Roche). The membrane fraction was obtained by 30-minute incubation at 4 °C, and cleared by 30-minute centrifugation at 100,000 x g, 4 ° C, then subjected to immunoprecipitation as previously described (Cao et al., 2015). The beads were washed three times with ice-cold IP buffer. Proteins were eluted with 50 μL SDS sample buffer (62 mM Tris, 10% glycerol, 2% SDS, and 5% β-mercaptoethanol), entered SDS-PAGE by applying ~150 mV for 15-20 minutes. Gels were fixed with using 5% acetic acid in 50% methanol, stained by NOVEX colloidal blue (Invitrogen). Stained areas were cut out, digested with trypsin (Promega), and alkylated as described previously (Shevchenko et al., 2006). The resulting peptide mixtures were desalted, resolved by high-pressure liquid chromatography, and analyzed using LTQ-Orbitrap XL mass spectrometer, as described previously (Posokhova et al., 2011).

Cell culture and transfection

HEK293T cells were obtained from Clontech and cultured at 37°C and 5% CO_2 in DMEM supplemented with antibiotics, 10% FBS. HEK293T cells were transfected at ~70%

confluency using Lipofectamine LTX (Invitrogen) according to the protocol of the manufacturer. The cells were harvested and preceded to co-immunoprecipitation.

Immunoprecipitation

Cells or retina were lysed in ice-cold PBS IP buffer by sonication followed by centrifugation at 14,000 x g for 15 minutes. The supernatant was incubated with 20 µl of 50% protein G slurry (GE Healthcare) and 5 µg antibodies on a rocker at 4°C for 1 hour. After three washes with IP buffer, proteins were eluted from beads with 50 µl of SDS sample buffer. Proteins retained by the beads were analyzed with SDS-PAGE, followed by Western blotting using HRP conjugated secondary antibodies and an ECL West Pico (Thermo Scientific) detection system. Signals were captured on film and scanned by densitometer.

In situ hybridization

Eyeballs were dissected out and put into 4 % paraformaldehyde for 15 minutes. After incubated in 30% sucrose overnight, 12-µm retina sections were moved to OCT and cut using a Leica CM3050 S cryostat, rinsed in PBS and incubated for 90 minutes in hybridization solution (50% deionized formamide, 5X SSC, 5X Denhardt's solution, 500 µg/ml yeast tRNA, 500µg/ml sonicated salmon sperm DNA) at 50 °C. Each section was incubated overnight with hybridization solution at 55 °C containing the Dig-labeled riboprobes. On the second day, each section was washed three times with 0.5X SSC and 30% formamide at 55 °C for 10 minutes, followed by additional three-time washes with PBS at room temperature. The sections were incubated with blocking buffer (10% goat serum, 0.15M NaCl, 0.1M Tris-Cl pH7.5) for 1 hour at room temperature, followed by incubation with anti-dig-AP conjugate (1:500 in blocking buffer)

overnight at 4 °C. After three 10-minute washes with washing buffer (0.15M NaCl, 0.1M Tris-Cl pH7.5), the endogenous peroxidase was inactivated with buffer Developer Buffer (0.5 mg/mL levamisole, 100mM Tris-HCl pH 10, 50mM MgCl2, 100mM NaCl) for 5 minutes. The color was detected by incubation with Developer Buffer with 0.45 µl/mL NBT and 3.5 µl/mL BCIP for 30 minutes. Then the reaction was terminated by TE (pH8). The sections were mounted and images were acquired using an optical microscope (Leica DM IL LED).

Fluorescence *in situ* hybridization

The mRNA expression was evaluated with ViewRNATM 2-plex In Situ Hybridization Assay (Panomics, Santa Clara, CA) using the following probes: *Lrit1* (NM_146245.2; Cat# VB1-17470). The whole eye bulb was extracted, embedded in OCT and flash frozen in liquid nitrogen. 12 µm sections were cut using a Leica CM3050 S cryostat, rapidly post-fixed in 4% paraformaldehyde for 10 minutes, washed twice in phosphate buffered saline (PBS) and incubated for 2h in pre-hybridization mix (50% deionized formamide, 5X SSC, 5X Denhardt's solution, 250 µg/ml yeast tRNA, 500µg/ml sonicated salmon sperm DNA) at room temperature. Each section was incubated overnight with Panomics hybridization solution (using an incubator set to 40ºC, no CO_2 and humidity higher than 85%) containing the QuantiGene ViewRNA probe set diluted 1:50 in Probe Set Diluent QT. On the second day, the retina sections were processed according to manufacturer's instructions provided with the ViewRNA ISH Tissue Assay Kit (QVT0012). Briefly, sections were successively incubated with PreAmplifier Mix QT, Amplifier Mix QT, Label Probe 1-AP (1:1000), AP-Enhancer Solution and Fast Red Substrate. Finally, the nuclei were counterstained with DAPI and mounted using Fluoromont-G (SouthernBiotech). Confocal images were generated at The Light Microscopy Facility, the Max Planck Florida

Institute, using a LSM 780 Zeiss confocal microscope. Image acquisition and processing were accomplished using ZEN 2011 (64 bit) software (Carl Zeiss) with only minor manipulations of the images setting the fluorescence intensity in non-saturating conditions and maintaining similar parameters for each acquired image

Immunohistochemistry

Dissected eyecups were fixed for 15 min in 4% paraformaldehyde, cryoprotected with 30% sucrose in PBS for 2 h at room temperature, and embedded in optimal cutting temperature medium. Twelve-micrometer frozen sections were obtained and blocked in PT1 (PBS with 0.1% Triton X-100 and 10% donkey serum) for 1 h, then incubated with primary antibody in PT2 (PBS with 0.1% Triton X-100 and 2% donkey serum) for at least 1 h. After four washes with PBS with 0.1% Triton, sections were incubated with fluorophore-conjugated secondary antibodies in PT2 for 1 h. After four washes, sections were mounted in Fluoromount (Sigma).
For LRIT3 and α2δ4 staining, antigen retrieval was done by incubating slides in basic antigen retrieval reagent (R&D system) preheated to ~80 degree for 5 min before blocking. Images were taken with a Leica SP800 or Zeiss LSM 880 confocal microscope. Quantitative analysis of LRIT1, mGluR6, GPR179, and ELFN1 immunofluorescence from confocal images was performed using Leica software or Zen Blue 2 analysis software. Sections were double stained with for marker protein mGluR6, which localizes at the synaptic puncta of ON-BC and was used as a mask to define synapses. The fluorescence intensity within synaptic puncta was analyzed using constant puncta-encircling area, which tightly surrounded the contours of each puncta. A line of 1-1.5 μm (white bar) was drawn through the center of the distinct mGluR6-positive synapses and the distribution of the fluorescence intensity along this line was scanned to generate

the traces. Mean fluorescent intensity (measured in pixels) was averaged from ~10-20 individual and randomly selected mGluR6-positive puncta per imaged section. For LRIT1 immunofluorescence in different animal models and different synaptic molecules staining the entire OPL was first selected using hand drawn tool in Zen Blue and the mean immunofluorescent intensity within OPL was calculated by the software. The mean fluorescent intensity of two to three sections per retina, and two to three retinas per genotype were used for final quantification and comparison. Imaging parameters were the same for all sections and retinas.

Electroretinography (ERG)

Electroretinograms were recorded by using the UTA system and a Big-Shot Ganzfeld (LKC Technologies). Mice (~ 4-8 weeks old) were dark-adapted (≥6 h) and prepared for recordings using a red dim light. Mice were anesthetized with an i.p. injection of ketamine and xylazine mixture containing 100 and 10 mg/kg, respectively. All procedures were approved by the Institutional Animal Care and Use committee at the Scripps Florida Research Institute. Recordings were obtained from the right eye only, and the pupil was dilated with 2.5% phenylephrine hydrochloride (Bausch & Lomb), followed by the application of 0.5% methylcellulose. Recordings were performed with a gold loop electrode supplemented with contact lenses to keep the eyes immersed in solution. The reference electrode was a stainless steel needle electrode placed subcutaneously in the neck area. The mouse body temperature was maintained at 37 °C by using a heating pad controlled by ATC 1000 temperature controller (World Precision Instruments). ERG signals were sampled at 1 kHz and recorded with 0.3-Hz low-frequency and 300-Hz high-frequency cut-offs.

Full field white flashes were produced by a set of LEDs (duration < 5 ms) for flash strengths ≤ 2.5 cd*s/m^2 or by a Xenon light source for flashes > 2.5 cd*s/m^2 (flash duration < 5 ms). ERG responses were elicited by a series of flashes ranging from $1\times 10^{-5}$to 800 cd*s/m^2 in 10-fold increments. Ten trials were averaged for responses evoked by flashes up to 0.1 cd*s/m^2, and three trials were averaged for responses evoked by 0.5 and 1 cd*s/m^2 flashes. Single flash responses were recorded for brighter stimuli. To allow for recovery, interval times between single flashes were as follows: 5 s for 1×10^{-5} to 0.1 cd*s/m^2, 30 s for 0.5 and 1 cd*s/m^2, 60 s for 5 and 10 cd*s/m^2, and 180 s for 100 and 800 cd*s/m^2 flashes. Light backgrounds of 50, 1, and 0.1 cd/m^2 were administered for 5 minutes for recording partially saturated rod- and cone-only ERGs. At rod saturating (cone-only) backgrounds, ten trials were averaged at an interval recovery time of 1 second between flashes.

ERG traces were analyzed using the EM LKC Technologies software and Microsoft Excel. The b-wave amplitude was calculated from the bottom of the a-wave response to the peak of the b-wave. The data points from the b-wave stimulus–response curves were fitted by Equation 1 using the least-square fitting method in GraphPad Prism6.

(1) $\mathbf{R=R_{max,r}*I/(I + I_{0.5, r}) + R_{max,c}*I/(I + I_{0.5,c})}$

The first term of this equation describes rod-mediated responses (r), and the second term accounts primarily for responses that were cone mediated (usually at flash intensities ≥1 cd*s/m^2 for dark-adapted mice; index c). $R_{max,r}$ and $R_{max,c}$ are maximal response amplitudes, and $I_{0.5,r}$ and $I_{0.5,c}$ are the half-maximal flash intensities. Stimulus responses of retina cells increase in proportion to stimulus strength and then saturate, this is appropriately described by the hyperbolic curves of this function.

The oscillatory potential traces were generated by transforming the original ERG traces using the built-in Oscillatory Potential Analysis function in EM LKC Technologies software which also calculated the amplitude of oscillatory potential.

For the flicker ERG response test, 10 trials were averaged from 3cd*s/m^2 flashes at a delivery rate of 7 Hz.

Electron Microscopy

Eyes were enucleated, cleaned of extra-ocular tissue, and pre-fixed for 15 min in cacodylate-buffered half-Karnovsky's fixative containing 2mM calcium chloride. Then the eyecups were hemisected along the vertical meridian and fixed overnight in the same fixative. The specimens were rinsed with cacodylate buffer and postfixed in 2% osmium tetroxide in buffer for 1 hour, then gradually dehydrated in an increasing ethanol and acetone series (30–100%), and embedded in Durcupan ACM resin (Electron Microscopy Sciences, PA). Blocks were cut with 70-nm-thickness, and were stained with 3% lead citrate. Sections were examined in a Tecnai G2 spirit BioTwin (FEI) transmission electron microscope at 80 or 100 kV accelerating voltage. Images were captured with a Veleta CCD camera (Olympus) operated by TIA software (FEI).

Single cell recordings from cones and bipolar cells, and light calibrations

Light-evoked responses from photoreceptors and bipolar cells were recorded retinal slices using methods described previously (Okawa et al., 2010). Briefly, mice were dark-adapted overnight and euthanized according to protocols approved by the University of California, Los Angeles Animal Research Committee (Protocol 14-005-11). Eyes were enucleated under infrared

light, retinas were isolated, and 200-μm thick slices were cut with a vibrating microtome. Slices were superfused with bicarbonate-buffered Ames' media (equilibrated with 5% CO_2/95% O_2) heated to 35-37°C, visualized under infrared illumination, and were stimulated with a blue light-emitting diode ($\lambda_{max} \sim$ 405nm).

Light-evoked responses were measured using patch electrodes in voltage-clamp mode (V_m = -40 mV for photoreceptor cells, V_m = -60 mV for bipolar cells), using an electrode internal solution consisting of (in mM): 125 K-aspartate, 10 KCl, 10 HEPES, 5 N-methyl-glucamine/HEDTA, 0.5 CaCl2, 1 ATP-Mg, and 0.2 GTP-Mg; pH was adjusted to 7.3 with N-methyl-glucamine hydroxide, and osmolarity was adjusted to 280 mOsm. Patch clamp recordings from cones additionally included 1 mM NADPH in the internal solution, which prevented response rundown. Light-evoked responses were sampled 10 kHz and filtered at 300 Hz with an 8-pole Bessel filter (Frequency Devices, Ottawa, IL). Data was further decimated and filtered offline at 50 Hz in Matlab (Mathworks, Natick, MA).

Recordings were made during experiments on WT and *Lrit1*$^{-/-}$ from cone photoreceptors, rod ON-BCs, and cone ON-BCs in retinal slices. The experimenter was blinded to the genotype of the animal until the recorded data was analyzed. We distinguished between bipolar cell types based on the polarity and time course of the response in conjunction with the cell's morphology. Recorded cells were visualized following their patch dialysis with a fluorophore (Alexa-750; Life Technologies) added to the electrode internal solution, allowing visualization in the far red without significant visual pigment bleaching. The responses of cones, and rod and cone ON-BCs were recorded from the same slices, and were typically adjacent to one another.

Light stimulation consisted of 10ms flashes of light that varied in strength from those yielding a just discernable response to those that generate a maximal response. Flash strengths are reported in activated cone pigment molecules (or P*/cone) at 405 nm, near the isosbestic point for S-cone and M-cone spectral sensitivities. To derive P*/cone, collecting areas were calculated from quantal responses at 405 nm recorded from control rods to derive the R*/rod. To calculate P*/cone, we adjusted this value for the difference in the volume of the cone vs. rod outer segment (cone/rod: $14\mu m^3/38\mu m^3$), which were then used to scale for the cone collecting area.

Evaluation of mouse vision by optokinetic reflex (OKR) test

Photopic contrast sensitivity of mice was evaluated from optomotor responses using a two-alternative forced-choice protocol, as previously described (Kolesnikov et al., 2011; Umino et al., 2008). Briefly, a mouse was placed on a pedestal surrounded by four computer monitors and observed from above using a camera. Mice responded to visual stimuli (sine-wave vertical gratings presented on the computer monitors using staircase paradigm and invisible to the experimenter), by reflexively rotating their head in either clockwise or counterclockwise direction. By looking at the very tip of animal's nose on a zoomed video view (140%), the observer registered the direction of optomotor responses and the computer determined the correctness of the choice (Prusky et al., 2004). In contrast to previous work (Kolesnikov et al., 2011; Umino et al., 2008), the duration of each trial was not strictly limited to 5 s, and the trial started only when the mouse was in a stable position on the pedestal, which could take up to several minutes. Photopic visual acuity was estimated as the threshold for spatial frequency of the stimuli at 100% contrast. Photopic contrast sensitivity was defined as the inverse of contrast threshold values which were obtained at fixed background luminance of monitors (1.1 cd m^{-2} at

the mouse eye level, as attenuated by neutral density film filters that formed a cylinder around the animal), over a range of various stimuli speeds (Sp), from 5 to 50 deg/s. Spatial frequency (Fs) of stimuli was kept constant at its optimal value of 0.128 cyc/deg, for a range of corresponding temporal frequencies (Ft = Sp*Fs) from 0.64 to 6.4 Hz (Umino et al., 2008). All data were analyzed using independent two-tailed Student *t*-test, with accepted significance level of $p < 0.05$.

Supplemental References

Cao, Y., Posokhova, E., and Martemyanov, K.A. (2011). TRPM1 forms complexes with nyctalopin in vivo and accumulates in postsynaptic compartment of ON-bipolar neurons in mGluR6-dependent manner. J Neurosci *31*, 11521-11526.

Cao, Y., Sarria, I., Fehlhaber, K.E., Kamasawa, N., Orlandi, C., James, K.N., Hazen, J.L., Gardner, M.R., Farzan, M., Lee, A.*, et al.* (2015). Mechanism for Selective Synaptic Wiring of Rod Photoreceptors into the Retinal Circuitry and Its Role in Vision. Neuron *87*, 1248-1260.

Cao, Y., Song, H., Okawa, H., Sampath, A.P., Sokolov, M., and Martemyanov, K.A. (2008). Targeting of RGS7/Gbeta5 to the dendritic tips of ON-bipolar cells is independent of its association with membrane anchor R7BP. J Neurosci *28*, 10443-10449.

Kolesnikov, A.V., Rikimaru, L., Hennig, A.K., Lukasiewicz, P.D., Fliesler, S.J., Govardovskii, V.I., Kefalov, V.J., and Kisselev, O.G. (2011). G-protein betagamma-complex is crucial for efficient signal amplification in vision. J Neurosci *31*, 8067-8077.

Okawa, H., Miyagishima, K.J., Arman, A.C., Hurley, J.B., Field, G.D., and Sampath, A.P. (2010). Optimal processing of photoreceptor signals is required to maximize behavioural sensitivity. J Physiol *588*, 1947-1960.

Posokhova, E., Song, H., Belcastro, M., Higgins, L., Bigley, L.R., Michaud, N.A., Martemyanov, K.A., and Sokolov, M. (2011). Disruption of the Chaperonin containing TCP-1 function affects protein networks essential for rod outer segment morphogenesis and survival. Mol Cell Proteomics *10*, M110 000570.

Prusky, G.T., Alam, N.M., Beekman, S., and Douglas, R.M. (2004). Rapid quantification of adult and developing mouse spatial vision using a virtual optomotor system. Invest Ophthalmol Vis Sci *45*, 4611-4616.

Shevchenko, A., Tomas, H., Havlis, J., Olsen, J.V., and Mann, M. (2006). In-gel digestion for mass spectrometric characterization of proteins and proteomes. Nature protocols *1*, 2856-2860.

Umino, Y., Solessio, E., and Barlow, R.B. (2008). Speed, spatial, and temporal tuning of rod and cone vision in mouse. J Neurosci *28*, 189-198.
